# Characterizing genetic intra-tumor heterogeneity across 2,658 human cancer genomes

**DOI:** 10.1016/j.cell.2021.03.009

**Published:** 2021-04-15

**Authors:** Stefan C. Dentro, Ignaty Leshchiner, Kerstin Haase, Maxime Tarabichi, Jeff Wintersinger, Amit G. Deshwar, Kaixian Yu, Yulia Rubanova, Geoff Macintyre, Jonas Demeulemeester, Ignacio Vázquez-García, Kortine Kleinheinz, Dimitri G. Livitz, Salem Malikic, Nilgun Donmez, Subhajit Sengupta, Pavana Anur, Clemency Jolly, Marek Cmero, Daniel Rosebrock, Steven E. Schumacher, Yu Fan, Matthew Fittall, Ruben M. Drews, Xiaotong Yao, Thomas B.K. Watkins, Juhee Lee, Matthias Schlesner, Hongtu Zhu, David J. Adams, Nicholas McGranahan, Charles Swanton, Gad Getz, Paul C. Boutros, Marcin Imielinski, Rameen Beroukhim, S. Cenk Sahinalp, Yuan Ji, Martin Peifer, Inigo Martincorena, Florian Markowetz, Ville Mustonen, Ke Yuan, Moritz Gerstung, Paul T. Spellman, Wenyi Wang, Quaid D. Morris, David C. Wedge, Peter Van Loo, on behalf of the PCAWG Evolution and Heterogeneity Working Group and the PCAWG Consortium, Stefan C. Dentro, Ignaty Leshchiner, Moritz Gerstung, Clemency Jolly, Kerstin Haase, Maxime Tarabichi, Jeff Wintersinger, Amit G. Deshwar, Kaixian Yu, Santiago Gonzalez, Yulia Rubanova, Geoff Macintyre, Jonas Demeulemeester, David J. Adams, Pavana Anur, Rameen Beroukhim, Paul C. Boutros, David D. Bowtell, Peter J. Campbell, Shaolong Cao, Elizabeth L. Christie, Marek Cmero, Yupeng Cun, Kevin J. Dawson, Nilgun Donmez, Ruben M. Drews, Roland Eils, Yu Fan, Matthew Fittall, Dale W. Garsed, Gad Getz, Gavin Ha, Marcin Imielinski, Lara Jerman, Yuan Ji, Kortine Kleinheinz, Juhee Lee, Henry Lee-Six, Dimitri G. Livitz, Salem Malikic, Florian Markowetz, Inigo Martincorena, Thomas J. Mitchell, Ville Mustonen, Layla Oesper, Martin Peifer, Myron Peto, Benjamin J. Raphael, Daniel Rosebrock, S. Cenk Sahinalp, Adriana Salcedo, Matthias Schlesner, Steven E. Schumacher, Subhajit Sengupta, Ruian Shi, Seung Jun Shin, Lincoln D. Stein, Oliver Spiro, Ignacio Vázquez-García, Shankar Vembu, David A. Wheeler, Tsun-Po Yang, Xiaotong Yao, Ke Yuan, Hongtu Zhu, Wenyi Wang, Quaid D. Morris, Paul T. Spellman, David C. Wedge, Peter Van Loo

**Affiliations:** 1 Cancer Genomics Laboratory, The Francis Crick Institute, London NW1 1AT, UK; 2 Wellcome Trust Sanger Institute, Cambridge CB10 1SA, UK; 3 Big Data Institute, University of Oxford, Oxford OX3 7LF, UK; 4 Broad Institute of MIT and Harvard, Cambridge, MA 02142, USA; 5 University of Toronto, Toronto, ON M5S 3E1, Canada; 6 Vector Institute, Toronto, ON M5G 1L7, Canada; 7 The University of Texas MD Anderson Cancer Center, Houston, TX 77030, USA; 8 Cancer Research UK Cambridge Institute, University of Cambridge, Cambridge CB2 0RE, UK; 9 Department of Human Genetics, University of Leuven, 3000 Leuven, Belgium; 10 University of Cambridge, Cambridge CB2 0QQ, UK; 11 Computational Oncology, Memorial Sloan Kettering Cancer Center, New York, NY 10065, USA; 12 Irving Institute for Cancer Dynamics, Columbia University, New York, NY 10027, USA; 13 German Cancer Research Center (DKFZ), 69120 Heidelberg, Germany; 14 Heidelberg University, 69120 Heidelberg, Germany; 15 Cancer Data Science Laboratory, National Cancer Institute, NIH, Bethesda, MD 20892, USA; 16 Simon Fraser University, Burnaby, BC V5A 1S6, Canada; 17 Vancouver Prostate Centre, Vancouver, BC V6H 3Z6, Canada; 18 NorthShore University HealthSystem, Evanston, IL 60201, USA; 19 Molecular and Medical Genetics, Oregon Health & Science University, Portland, OR 97231, USA; 20 University of Melbourne, Melbourne, VIC 3010, Australia; 21 Walter + Eliza Hall Institute, Melbourne, VIC 3000, Australia; 22 Weill Cornell Medicine, New York, NY 10065, USA; 23 New York Genome Center, New York, NY 10013, USA; 24 Cancer Evolution and Genome Instability Laboratory, The Francis Crick Institute, London NW1 1AT, UK; 25 University of California, Santa Cruz, Santa Cruz, CA 95064, USA; 26 Cancer Research UK Lung Cancer Centre of Excellence, University College London Cancer Institute, London WC1E 6BT, UK; 27 Cancer Genome Evolution Research Group, University College London Cancer Institute, London WC1E 6DD, UK; 28 Department of Medical Oncology, University College London Hospitals, London NW1 2BU, UK; 29 Massachusetts General Hospital Center for Cancer Research, Charlestown, MA 02129, USA; 30 Massachusetts General Hospital, Department of Pathology, Boston, MA 02114, USA; 31 Harvard Medical School, Boston, MA 02215, USA; 32 Ontario Institute for Cancer Research, Toronto, ON M5G 0A3, Canada; 33 University of California, Los Angeles, Los Angeles, CA 90095, USA; 34 Dana-Farber Cancer Institute, Boston, MA 02215, USA; 35 The University of Chicago, Chicago, IL 60637, USA; 36 Department of Translational Genomics, Center for Integrated Oncology Cologne-Bonn, Medical Faculty, University of Cologne, 50931 Cologne, Germany; 37 Organismal and Evolutionary Biology Research Programme, Department of Computer Science, Institute of Biotechnology, University of Helsinki, 00014 Helsinki, Finland; 38 School of Computing Science, University of Glasgow, Glasgow G12 8RZ, UK; 39 European Molecular Biology Laboratory, European Bioinformatics Institute (EMBL-EBI), Cambridge CB10 1SD, UK; 40 European Molecular Biology Laboratory, Genome Biology Unit, 69117 Heidelberg, Germany; 41 Computational and Systems Biology, Memorial Sloan Kettering Cancer Center, New York, NY 10065, USA; 42 Oxford NIHR Biomedical Research Centre, Oxford OX4 2PG, UK; 43 Manchester Cancer Research Centre, University of Manchester, Manchester M20 4GJ, UK

**Keywords:** whole-genome sequencing, pan-cancer genomics, intra-tumor heterogeneity, cancer driver genes, cancer evolution, tumor phylogeny, subclonal reconstruction, branching evolution

## Abstract

Intra-tumor heterogeneity (ITH) is a mechanism of therapeutic resistance and therefore an important clinical challenge. However, the extent, origin, and drivers of ITH across cancer types are poorly understood. To address this, we extensively characterize ITH across whole-genome sequences of 2,658 cancer samples spanning 38 cancer types. Nearly all informative samples (95.1%) contain evidence of distinct subclonal expansions with frequent branching relationships between subclones. We observe positive selection of subclonal driver mutations across most cancer types and identify cancer type-specific subclonal patterns of driver gene mutations, fusions, structural variants, and copy number alterations as well as dynamic changes in mutational processes between subclonal expansions. Our results underline the importance of ITH and its drivers in tumor evolution and provide a pan-cancer resource of comprehensively annotated subclonal events from whole-genome sequencing data.

## Highlights

• Pan-cancer resource of comprehensively annotated intra-tumor heterogeneity (ITH)
• ITH is pervasive across cancers and shows cancer type-specific patterns
• Branching phylogenies are common
• Dynamic changes in mutational processes between subclonal expansions

## Graphical abstract

**Figure:**
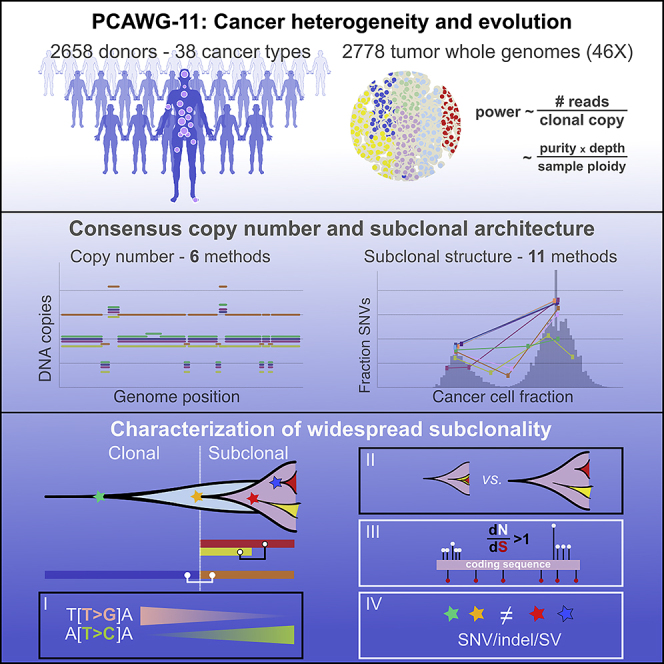

## Teaser

Dentro et al. provide a comprehensive annotation of intra-tumor heterogeneity and its drivers in cancer evolution.

## Introduction

Cancers accumulate somatic mutations as they evolve (Nowell, 1976; Tabin et al., 1982). Some of these mutations are drivers that confer fitness advantages to their host cells and can lead to clonal expansions (Garraway and Lander, 2013; Greaves and Maley, 2012; Stratton et al., 2009; Vogelstein et al., 2013). Late clonal expansions, spatial segregation, and incomplete selective sweeps result in genetically distinct cellular populations that manifest as intra-tumor heterogeneity (ITH) (Nowell, 1976). Clonal mutations are shared by all cancer cells, whereas subclonal mutations are present only in a subset.

ITH is an important clinical challenge because it provides genetic variation that may drive cancer progression and lead to emergence of drug resistance (Maley et al., 2006; McGranahan and Swanton, 2017; Mroz et al., 2013). Subclonal drug resistance and associated driver mutations are common (Gerlinger et al., 2012; Gundem et al., 2015; Landau et al., 2013; McGranahan et al., 2015; Shaw et al., 2016; Yates et al., 2015). ITH can affect clinical trial design (Hiley et al., 2014) and predict progression (Maley et al., 2004) and prognosis (Espiritu et al., 2018). For example, ITH at the level of copy number alterations (CNAs) is associated with increased risk of relapse in non-small cell lung cancer (Jamal-Hanjani et al., 2017), head and neck cancer (Mroz and Rocco, 2013; Rocco, 2015), and glioblastoma multiforme (Brastianos et al., 2017).

ITH can be characterized from massively parallel sequencing data (Campbell et al., 2008; Landau et al., 2013; McGranahan et al., 2015; Nik-Zainal et al., 2012) because the cells comprising a clonal expansion share the unique set of mutations of the initiating cell. Each of those mutations is present in the same cancer cell fraction (CCF), which may be estimated by adjusting mutation allele frequencies for local copy number and sample purity. Subsequent clustering of mutations based on their CCF yields the “subclonal architecture” of a sample (Dentro et al., 2017): estimates of the number of distinct tumor cell populations (subclones), their CCF, and assigned mutations.

ITH remains poorly characterized across cancer types, and there is substantial uncertainty concerning the selective pressures operating on subclonal populations. Previous pan-cancer efforts have relied on exomes, restricting somatic mutation calling and subclonal resolution (Andor et al., 2016). Recent studies have relied on multi-region whole-genome, exome, or targeted sequencing to explore ITH in specific cancer types (Jamal-Hanjani et al., 2017; McPherson et al., 2016; Turajlic et al., 2018; Yates et al., 2015). Single-sample analyses can underestimate ITH because variants found to be clonal in one region may be subclonal in another (the “illusion of clonality”) (de Bruin et al., 2014). Conversely, any mutations detected as subclonal will remain so, no matter how many regions are assayed. Therefore, single-sample analysis establishes a conservative lower limit of ITH.

Here we develop a robust consensus strategy to call copy number and cluster mutations to assess ITH and its origin, drivers, and role in tumor development. We apply these approaches to 2,658 tumors from 38 histologically distinct cancer types from the Pan-Cancer Analysis of Whole Genomes (PCAWG) initiative (ICGC/TCGA Pan-Cancer Analysis of Whole Genomes Consortium, 2020). Whole-genome sequencing provides orders of magnitude more point mutations than exomes, greater resolution to detect CNAs, and the ability to call structural variants (SVs). These increase the breadth and depth of our analyses and reveal pervasive ITH across cancer types. We observe branching patterns of evolution and positive selection in subclones. We identify subclonal driver mutations in cancer genes and recurrent changes in mutation signature activity. Our analyses provide detailed insight into tumor evolutionary dynamics.

## Results

### Consensus-based characterization of ITH in 2,658 cancers

We leveraged the PCAWG dataset to characterize ITH across cancer types, including single-nucleotide variants (SNVs), insertions or deletions (indels), SVs, and CNAs as well as subclonal drivers, subclonal selection, and mutation signatures (Alexandrov et al., 2020; Gerstung et al., 2020; Rheinbay et al., 2020; ICGC/TCGA Pan-Cancer Analysis of Whole Genomes Consortium, 2020).

**Figure 1:**
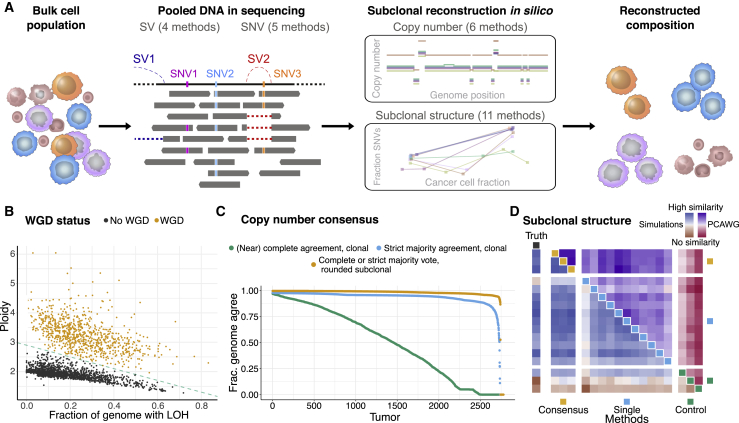
Consensus-based characterization of ITH (A) Schematic representation of our consensus-based ITH reconstruction. (B) Samples with and without WGD separate according to their consensus ploidy and the fraction of the genome showing loss of heterozygosity. (C) Agreement between the six copy number callers using a multi-tier consensus copy number calling approach. The three lines denote the fraction of the genome at which agreement is reached at different levels of confidence (STAR Methods). (D) Heatmap of the normalized average pairwise similarities of subclonal architectures identified by 11 individual, 3 consensus, and 3 control reconstruction methods. Each method is represented by one colored square on the diagonal. In rows and columns, each method is compared with all other methods. The upper triangle shows the similarities on the 2,658 PCAWG samples and the lower triangle on a validation set of 965 simulated samples. In the leftmost column, similarities are computed against the truth of the simulated set. Color intensities scale with the similarities and were normalized separately for PCAWG, simulations, and truth.

First, to generate high-confidence calls, we developed ensemble approaches for variant calling, copy number calling, and subclonal reconstruction (Figure 1A; STAR Methods). Specifically, to maximize the sensitivity and specificity of calling clonal and subclonal mutations, a consensus approach was adopted, integrating the output of five SNV calling algorithms (ICGC/TCGA Pan-Cancer Analysis of Whole Genomes Consortium, 2020). Similar approaches were employed for indels and SVs.

Because the quality of copy number calls has a large effect on subclonal reconstruction (Salcedo et al., 2020), we systematically integrated results from six copy number callers (Figure 1A; STAR Methods). A first run of each algorithm was used to identify all copy number breakpoints and construct a consensus segmentation. To improve sensitivity and obtain base-pair-resolution segments, SV breakpoints were also inserted (STAR Methods). In a second run of all CNA callers, this consensus segmentation was enforced, resulting in copy number calls with near-identical breakpoints across algorithms.

**Figure S1:**
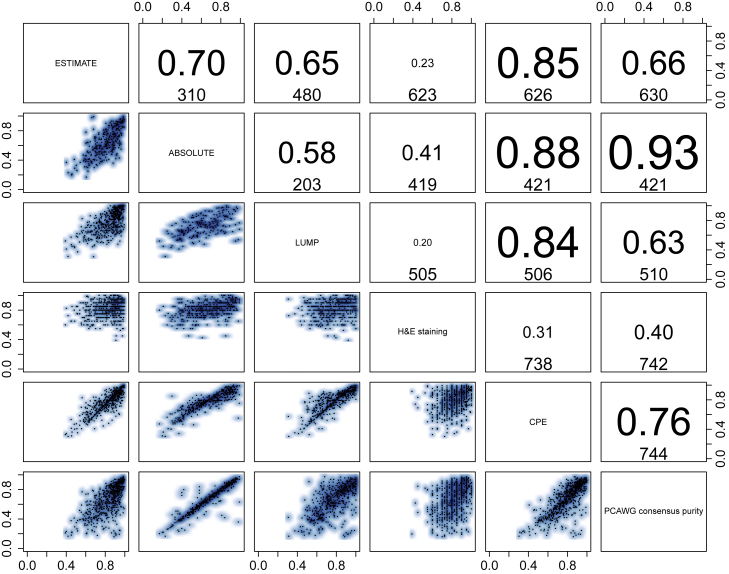
Validation of consensus purity values, related to Figure 1 The lower triangle shows pairwise scatterplots of the purities obtained through expression profiles of a panel of immune and stromal genes (ESTIMATE), somatic copy number data (ABSOLUTE), leukocyte unmethylation (LUMP), image analysis by hematoxylin and eosin staining (H&E staining), and consensus purity as derived by Aran et al., 2015 (CPE). The top triangle shows the respective Pearson correlation coefficients and the number of samples that have both purity estimates available.

Purity and ploidy assessment can be challenging because multiple combinations are theoretically possible for some cancer samples and may be difficult to distinguish (Carter et al., 2012; Van Loo et al., 2010). Consensus was obtained by establishing agreement between the six CNA callers (STAR Methods). An expert panel reviewed and resolved cases where the callers disagreed. Our purity values correlate strongly with a recent cross-omics analysis of tumor purity (Aran et al., 2015; Figure S1). We next defined samples that had undergone whole-genome duplication (WGD) in an objective and automated way, based on tumor ploidy and the extent of loss of heterozygosity (Figure 1B; STAR Methods). Samples with WGD evidenced synchronous chromosomal gains (Gerstung et al., 2020), further validating our approach. Last, CNAs were assigned “tiers” for the confidence in the consensus copy number, based on caller agreement. We reached high consensus confidence on a median 95% of the genome (Figure 1C; STAR Methods).

Consensus copy number, SNVs, and purity estimates served as input to 11 subclonal reconstructing methods, and the results of these methods were combined into a single consensus for each tumor (Figure 1A; STAR Methods). Addressing the probabilistic nature of subclonal reconstruction, we developed three consensus approaches using different summary outputs of individual methods. We validated consensus results on two independent simulated datasets and assessed their robustness on the real data. The consensus methods performed comparably with the best individual methods on both simulated datasets, with the top-performing individual methods also displaying high similarity scores (Figure 1D; STAR Methods). On the real data, the highest similarities were also observed between the individual and consensus approaches but not among individual methods (Figure 1D), confirming that a consensus yields the most robust subclonal reconstruction. Furthermore, the most robust performance, when the best methods are not known *a priori*, was achieved when all methods were included (STAR Methods). Hence, we used the output of one of our consensus approaches, combining all individual methods, as the basis for our global assignment strategy (STAR Methods). We thus obtained, for each tumor, the number of detectable subclones; the fraction of subclonal SNVs, indels, SVs, and CNAs; as well as assignments of SNVs, indels, and SVs to subclones.

**Figure 2:**
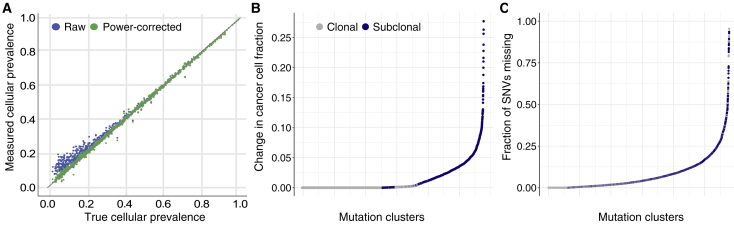
CCF and subclonal mutation number correction (A) Validation of our approach to adjust for the overestimation effect at the lower bound of CCF. (B and C) The estimated cluster CCF (B) and SNV count (C) adjustment in all identified mutation clusters (ranked on the x axis according to effect size on the y axis). Subclonal clusters show a shift to smaller CCF values after correction (B), and the majority of clusters are estimated to contain additional missed SNVs (C).

To obtain unbiased estimates of the number of mutations in subclones and their CCF, we accounted for a detection bias introduced by somatic variant calling. Specifically, as the CCF of a subclone decreases, so does the power to detect its unique SNVs. This leads to overestimation of the subclone’s CCF (Nik-Zainal et al., 2012) and underestimation of its mutation burden. The large number of SNVs revealed by whole-genome sequencing (WGS) facilitates quantitation and correction of these biases. We developed two methods to this end, validated them on simulated data (STAR Methods; Figure 2A), and combined them to correct the estimated number of SNVs and CCF of each subclone. On average, 14% of SNVs in observable subclones fall below the detection limit (Figures 2B and 2C). In subclones with a CCF of less than 30%, an average 21% of SNVs are missed. These values reflect SNVs missed in detected subclones, not in subclones that remain undetected because of limited sequencing depth. Because of the complexity in modeling sensitivity of indel and SV calling as a function of the number of variant reads, methods were not extended to these mutation types. We anticipate that higher fractions of SVs and indels are missed because of the lower sensitivity of existing algorithms (ICGC/TCGA Pan-Cancer Analysis of Whole Genomes Consortium, 2020).

### Pervasive ITH across cancer types

**Figure S2:**
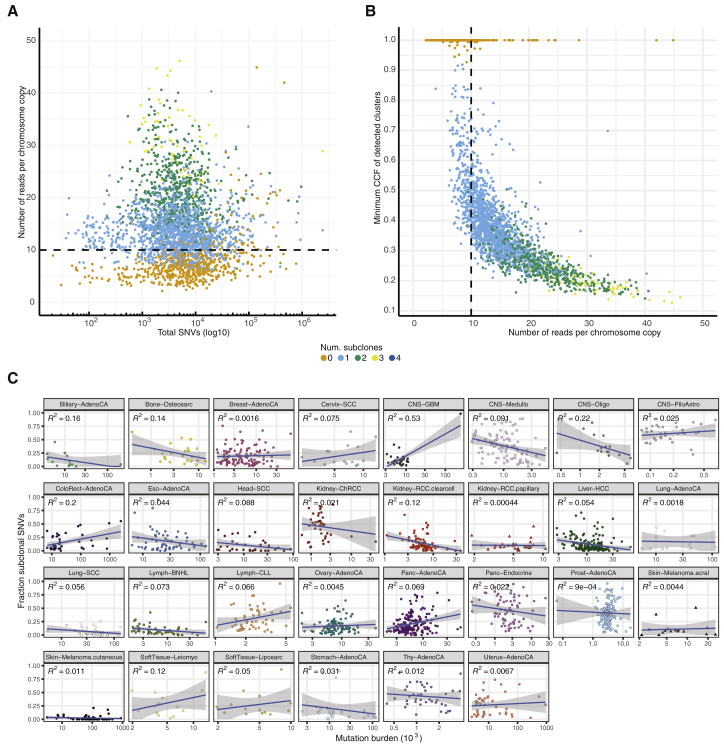
Power analysis of the consensus subclonal architecture approach, related to Figure 3 (A) Our ability to detect subclones depends, not on the number of detected SNVs, but on the number of reads per tumor chromosomal copy (nrpcc) available. This metric takes tumor purity, ploidy and sequencing coverage into account (see STAR Methods). We control for this effect by including only tumors with nrpcc ≥ 10. In these tumors, we should be sufficiently powered to detect a subclone at a CCF ≥ 30% (see STAR Methods), as evidenced (B) which shows the minimum CCF of the detected clusters in each tumor against the number of reads per chromosome copy. (C) The fraction of subclonal mutations per sample does not show significant correlation with mutation burden across cancer types.

**Figure 3:**
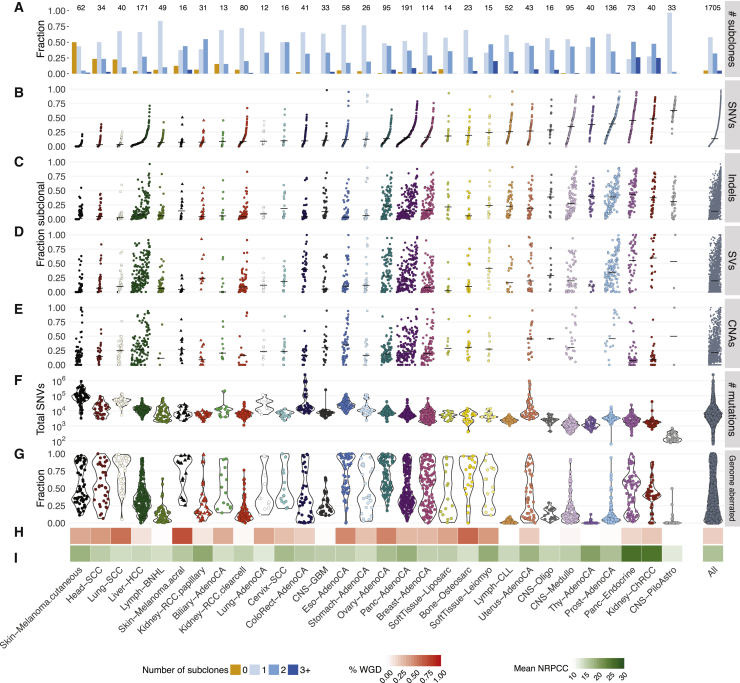
Overview and characterization of ITH across cancer types Evidence of ITH is shown for 1,705 samples with sufficient power to detect subclones at a CCF of more than 30% (STAR Methods). (A) Bar plot showing the fraction of samples with a given number of distinctive subclonal expansions. (B–E) Scatterplots showing the fractions of SNVs (B), indels (C), SVs (D), and arm-level CNAs (E) that were classified as subclonal. For SVs and CNAs, only samples with 5 or more events are plotted. Samples are ordered by increasing fraction of subclonal SNVs. (F and G) Violin plots showing total mutation burden (F) and fraction of the genome with CNAs (G). (H and I) Heatmaps showing the fraction of tumor samples with whole genome duplications (H) and the mean power to identify subclones per cancer type (nrpcc; STAR Methods) (I).

**Figure S3:**
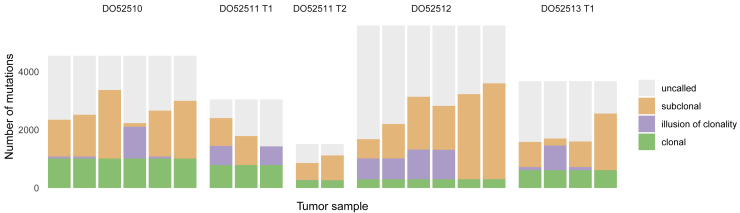
Illusion of clonality in single-sample versus multi-sample analysis, related to Figure 3 Comparison of clonality assignments and missed variants based on multi-region versus single-sample analyses for the five multi-region sequenced primary tumors in PCAWG. Subclonal mutations appearing to be clonal in the indicated sample display the illusion of clonality. Mutations detected in some, but not in other samples of the same tumor, are classified as uncalled in the latter samples.

Evaluating evidence for subclonal expansions across cancers in our consensus, we noted that the power to identify subclones depends on sample purity, tumor ploidy, and sequencing depth. We summarized these into one metric, the number of reads per tumor chromosomal copy (nrpcc; representing the sequencing coverage per haploid genome; Figure S2; STAR Methods), and focused on 1,705 genomes where our approach is powered to detect subclones encompassing more than 30% of tumor cells (STAR Methods). One or more subclonal expansions were evident in 1,621 tumors (95.1%) at the resolution of our methods (Figure 3A). Importantly, these estimates, based on single-sample reconstruction and a median ∼46× read coverage, provide a conservative lower bound because we are not powered to detect rare populations. In addition, because typically only part of the tumor is sequenced, some subclones may not be sampled, and some mutations may be subject to the illusion of clonality. Analysis of five distinct multi-region sequenced primary prostate tumors in PCAWG suggests that, on average, in our single-sample analysis, 44% of variants are missed, and 26% of subclonal mutations appear to be clonal (Figure S3), in concordance with published literature (Werner et al., 2017).

Our approach finds more than 75% of samples showing evidence of at least one subclonal expansion in all cancer types (Figure 3A), except cutaneous melanoma, where subclones were detectable by our approach in only 31 of 62 samples. Twenty-five of 30 cancer types with 10 cases or more comprised more than 90% of samples with at least one detectable subclone.

The fraction of subclonal SNVs identified (after correction for missed SNVs) varies widely across cancer types (Figure 3B). Squamous cell carcinomas typically show low fractions of subclonal SNVs (head and neck, 9.7% ± 11.9%; lung, 6.1% ± 6.7%; cervix, 20.6% ± 19.6%; mean ± standard deviation), whereas chromophobe renal cell carcinomas (45.2% ± 22.7%), pancreatic neuroendocrine tumors (46% ± 24.7%), and pilocytic astrocytomas (61.3% ± 14.8%) showed the highest.

**Figure S4:**
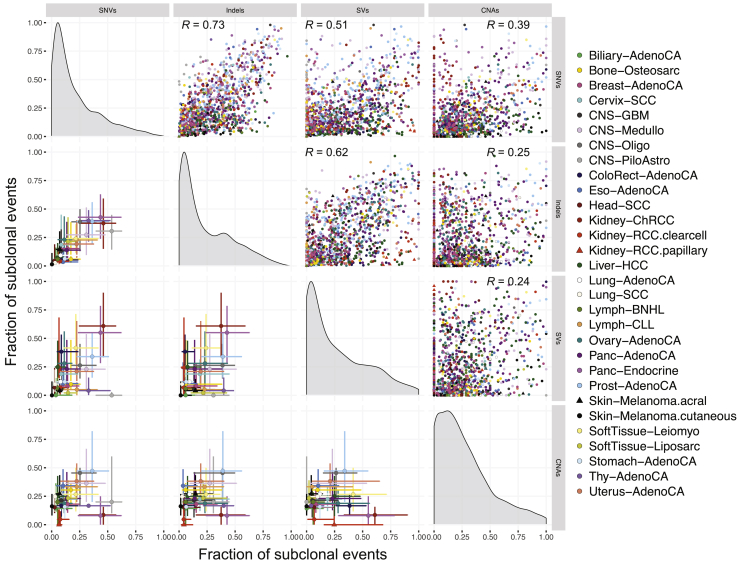
Correlation in ITH between SNVs, indels, CNAs, and SVs by cancer type, related to Figure 3 Evidence of ITH is shown for 1,705 samples with sufficient power to detect subclones above 30% CCF (see STAR Methods), as in Figure 3. Pairwise scatterplots in the upper triangle show the fractions of subclonal SNVs, indels, CNAs and SVs per tumor sample. Pearson’s correlation coefficient (*R*) is separately computed for each panel across all samples. Panels on the diagonal show the kernel density estimate of the distribution of subclonal fractions. In the lower triangle, each point shows the median subclonal fraction per cancer type and intervals indicate the interquartile range. Panels only include samples with at least 5 arm-level CNAs (1,238 / 1,705) and at least 5 SVs (1,609 / 1,705).

Indels, SVs, and CNAs also revealed similarly large differences between cancer types. For indels, the subclonal fraction ranged from 6.2% ± 11.7% in lung squamous carcinomas to 43.4% ± 23.7% in pancreatic neuroendocrine tumors (Figure 3C). For SVs, liposarcomas and cutaneous melanomas showed the lowest subclonal fraction (8.0% ± 14.1% and 8.9% ± 15.5%, respectively) and chromophobe renal cell carcinomas the highest (56.8% ± 31.6%) (Figure 3D). The fraction of subclonal copy number changes was lowest in chromophobe renal cell cancers (13.3% ± 16.8%) and highest in prostate adenocarcinoma (53.8% ± 32.0%) (Figure 3E). Comparing these values with the SNV burden, the fraction of the genome affected by CNAs, the frequency of WGD per cancer type, and our nrpcc power measure showed that none of these metrics explain this wide variation (Figures 3F–3I). Although we observed that cancer types with a higher mutation burden showed lower fractions of subclonal SNVs (Figures 3B and 3F), we did not see a similar relationship when evaluating individual tumors (STAR Methods). The proportions of subclonal indels and SNVs are strongly correlated (Pearson's *R*^*2*^ = 0.73), and SVs follow a similar trend (*R*^*2*^ = 0.62 with indels, *R*^*2*^ = 0.51 with SNVs) (Figures 3B–3E and S4). In contrast, the average proportions of subclonal large-scale CNAs and SNVs are only weakly correlated (*R*^2^ = 0.24), indicating that these could be driven by independent mutational processes or subject to differential selective pressures over time (Gerstung et al., 2020).

Some cancer types had limited ITH across all mutation types (e.g., squamous cell carcinomas), whereas others showed an abundance of ITH in specific categories. For example, pancreatic neuroendocrine tumors have few subclonal CNAs but a high subclonal burden across all other variant categories (Figures 3B–3E). Finally, the fraction of subclonal variants varies substantially among the tumors of each cancer type (Figures 3B–3E).

These findings highlight pervasive ITH across cancer types. Nearly all tumors display subclonal expansions, even at limited read depth. In addition, the average proportions of subclonal SNVs, indels, SVs, and CNAs are highly variable across cancer types. These analyses paint characteristic portraits of the nature of ITH, suggesting distinct evolutionary narratives for each cancer type.

### Complex phylogenies among subclones revealed by whole-genome sequencing

**Figure 4:**
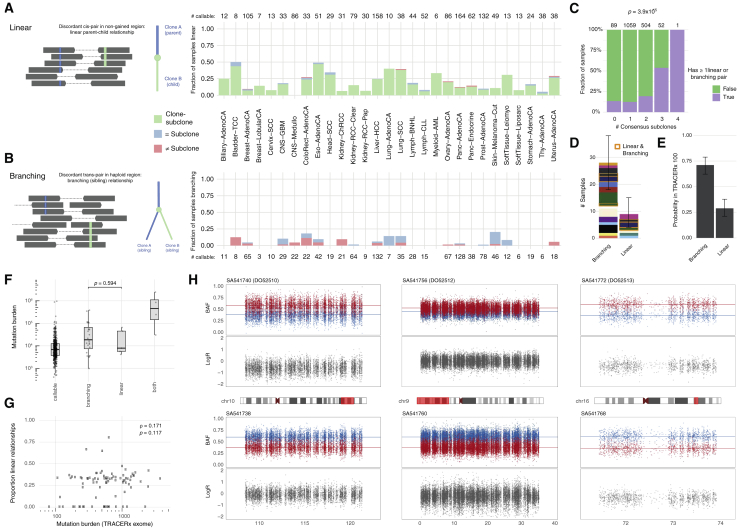
Further characterization of ITH using mutation phasing (A and B) Proportion of powered tumors with evidence of linear and branching phylogenies through analysis of phased reads of variants in *cis* (A) or in *trans* (B) among tumors with at least one phaseable pair in the appropriate context. (C) Fraction of powered samples, stratified by number of consensus subclones, with at least one linear or branching pair ($\chi^{2}$ test for independence). (D) Number of samples with linear or branching pairs when sets are filtered to be comparable. Error bars indicate the 95% bootstrap interval. Samples are colored by tumor type and boxed (orange) when they present with pairs of both types. (E) Probabilities of observing a linear versus branching relationship when picking two random subclones from the TRAcking non-small cell lung Cancer Evolution through therapy [Rx] TRACERx 100 trees (Jamal-Hanjani et al., 2017). Error bars indicate the 95% bootstrap interval. (F) Mutation burden distribution for comparable samples reporting only non-informative phased SNV pairs and linear and/or branching pairs (Wilcoxon rank-sum test). Horizontal lines indicate the 25th, 50th, and 75th percentiles while whiskers extend to the most extreme observation no further than 1.5 times the interquartile range. (G) Proportion of linear subclone-subclone relationships versus exonic mutation burden in the TRACERx100 cohort (Spearman’s rank correlation and test for deviation from zero). (H) B allele frequency (BAF) and LogR at germline heterozygous SNPs across example loci exhibiting a mirrored subclonal allelic imbalance between sample pairs from three multi-region sequenced prostate tumors. Parental alleles are colored consistently (red and blue) within each sample pair (top and bottom), highlighting parallel gains or losses of alternate alleles.

Two subclones can be linearly related to each other (parent-child relationship) or develop on branching lineages (sibling subclones). Establishing evolutionary relationships between subclones is challenging on single-sample sequencing data because of the limited resolution to separate subclones and the uncertainties regarding their CCF estimates. We can, however, examine pairs of SNVs that are covered by the same read pairs (i.e., phaseable SNV pairs). Specifically, evidence of a parent-child relationship is given by a pattern where, in a region without copy number gains, the SNV attributed to the child is only found on a subset of the reads that carry the SNV of the parent (discordant in *cis*; Figure 4A). Similarly, evidence of a sibling relationship is given by a pattern where, in a haploid region, overlapping read pairs carry the SNV attributed to one clone or the other but not both (discordant in *trans*; Figure 4B). The number of informative variant pairs is generally extremely low and depends on mutation burden and copy number. The PCAWG dataset, however, enables us to identify a sizeable total and explore their phylogenetic information content (STAR Methods).

Of 1,537 tumors with sufficient power and at least one phaseable pair in the correct context, 245 show discordant in *cis* SNVs pairs, indicating parent-child relationships (Figure 4A). Annotating SNVs with their consensus clone or subclone assignment, the vast majority of these samples (233, 95.1%) show pairs corroborating the expected clone-subclone relationship. In addition, there are 8 samples with pairs assigned to different subclones and 16 samples with pairs assigned to the same subclone, indicating linear evolution among subclones. Of 995 powered tumors, 51 carry discordant in *trans* SNVs pairs, with 32 and 27 of these having pairs assigned to the same or different subclones, respectively (8 have both), supporting the occurrence of sibling subclones (Figure 4B).

Identification of subclones from phaseable SNV pairs is largely independent of the consensus subclonal reconstruction. One can therefore use phasing results to assess the performance of the reconstruction and vice versa. Indeed, tumors identified to contain higher numbers of subclones are enriched for linear and branching pairs (p = 3.9 × 10^−15^; Figure 4C). Nevertheless, our identification of 16 and 32 samples with SNV pairs assigned to the same subclone but showing in *cis* and in *trans* discordance, respectively, confirms that our consensus approach identifies only a lower limit on the number of subclonal expansions. Interestingly, 8 cutaneous melanomas deemed clonal by the consensus reconstruction had phasing evidence for 1–2 subclones, suggesting that the large majority of cutaneous melanomas also contain subclonal expansions. However, these might be obscured by large numbers of clonal mutations in these tumors.

The frequency of branching versus linear evolution can be assessed directly by restricting the phasing analysis to haploid regions and to pairs where both SNVs have been assigned to subclones. In this subset, linear and branching relationships may be detected with similar power. We find that, in the pan-cancer setting, two subclones are 3.11-fold more likely to have sibling than parent-child relationships (bootstrapped 95% confidence interval [1.71; 7.50]; Figure 4D). This is consistent with phylogenies obtained from multi-region studies such as the TRACERx lung cancer cohort (Jamal-Hanjani et al., 2017), where the odds are 2.86 (bootstrapped 95% confidence interval [1.93; 5.07]; Figure 4E; STAR Methods), and with observations of mutual exclusivity of subclonal drivers and extensive parallel evolution (Turajlic et al., 2018).

This branching-to-linear ratio appears to be independent of mutation rate. Although the statistical power is limited, we do not observe a difference in mutation burden between callable samples reporting linear versus branching read pairs (Figure 4F; Wilcoxon rank sum test, p = 0.594). Similarly, the TRACERx cohort shows no correlation between mutation burden and the proportion of linear versus branching subclone relations (Figure 4G; Spearman’s rho = 0.171, p = 0.117). We could also detect branching evolution in low-mutation-burden, multi-region-sequenced primary tumors from individuals with early-onset prostate cancer in PCAWG (21 regions, 5 independent tumors, 2–6 regions each). Haplotype phasing and tracking alleles across regions using the Battenberg algorithm (Nik-Zainal et al., 2012) reveals loci with mirrored subclonal allelic imbalance in three of five tumors (Figure 4H; STAR Methods). These patterns of gains or losses of alternate alleles typically arise through parallel evolution of sibling subclones (Watkins et al., 2020), in line with previous efforts on a subset of these cases (Gerhauser et al., 2018). Our results suggest that branching evolution is the norm across tumor types, even in those with low mutation rates.

### Patterns of subclonal mutation signature activity changes across cancers

Mutation processes can differ in activity between clonal and subclonal lineages (McGranahan et al., 2015). To explore their dynamics in detail, we examined subclonal mutations for changes in signature activity. We reasoned that if a mutation process is activated during a subclonal expansion, then only the post-expansion mutations will carry the corresponding signature. Signature activity change points can therefore be identified in SNVs rank ordered and binned by their copy number and CCF to reflect pseudo-time (Rubanova et al., 2020; STAR Methods). This approach is complementary to the one in our recent study (Gerstung et al., 2020), which averages signatures across subclones. Of the 2,552 samples with sufficient SNVs, 1,944 (76%) have an activity change of more than 6% in 1 or more signatures (a conservative threshold established via permutation and bootstrapping; STAR Methods). We detect an average of 1.77 mutation signature activity changes per sample.

**Figure 5:**
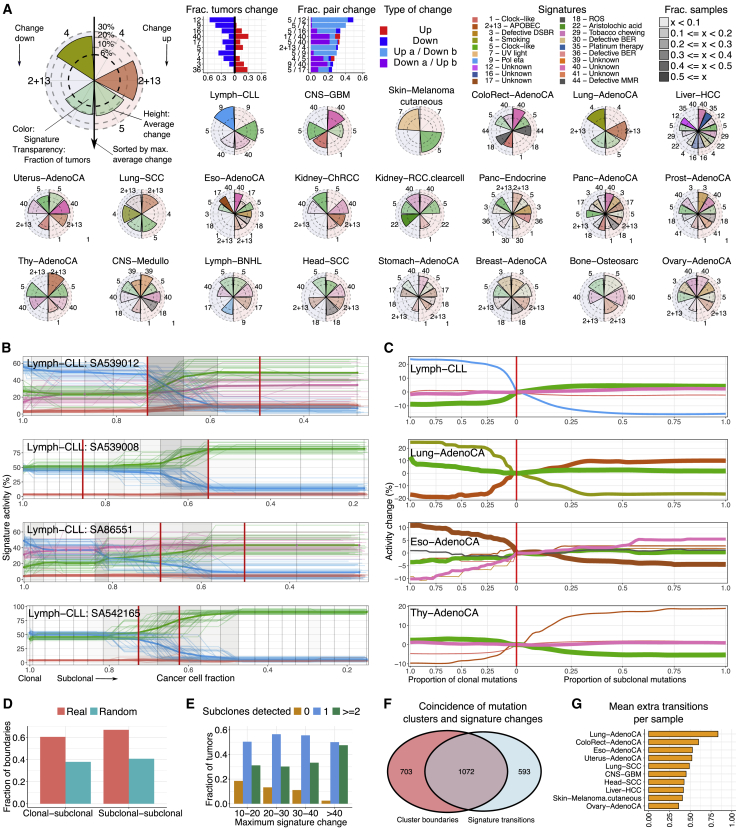
Subclonal boundaries are associated with changes in mutation signature activity (A) Mutation signature changes across cancer types. Bar graphs show the proportion of tumors in which signature (pairs) change, and radial plots provide a view per cancer type. Each radial plot contains the signatures that are active in 5 or more tumors and change (>6%) in at least 3. The left and right sides of radial plots represent signatures that become less and more active, respectively. The height of a wedge represents the average activity change (log scale), the color denotes the signature, and the transparency shows the fraction of tumors in which the signature changes (as a proportion of tumors with that signature). Signatures are sorted from top to bottom by their average activity change. (B) Signature activity trajectories in four CLLs. Each horizontal line, colored by signature, is an inferred signature activity trajectory across pseudotime, as defined by the SNVs rank ordered by CCF and binned. Thin and bold lines reflect the individual bootstrapped replicates and their average, respectively. Vertical lines indicate time points placed at the average CCF of the binned SNVs, whereas the shading in between denotes the frequency of significant activity changes. Red vertical lines mark boundaries between consensus subclonal mutation clusters. (C) Average signature trajectories for selected cancer types. Each line, colored by signature, corresponds to the average activity across tumors of this cancer type in which the signature is active. Line width reflects the number of contributing tumors. Trajectories are centered around the activity at the boundary between clonal and subclonal SNVs (vertical red line) to highlight relative changes. (D) Fractions of observed and randomly placed signature change points that coincide with boundaries between mutation clusters. (E) Number of subclones detected in tumors grouped by the maximum signature activity change. (F) Venn diagram of coinciding SNV cluster boundaries and signature activity change points. (G) Mean number of additional signature change points detected per tumor.

Overall, in accordance with our recent findings (Gerstung et al., 2020), mutation signature activity is remarkably stable between the clone and subclones. The most frequently changing signature (signature SBS12, etiology unknown [Alexandrov et al., 2020], active in 198 of 326 [61%] liver cancers), is variable in ∼60% of cases in which it is active (Figure 5A). Signature SBS9 activity (DNA polymerase Pol η associated with activation-induced deaminase [AID]) decreases along with CCF in over half of the tumors in which it is active (Chronic Lymphocytic Leukemia (CLL) and and B cell non-Hodgkin lymphoma).

We next evaluated signature trajectories per cancer type (Figure 5A). In CLL, SBS9 always decreases, and SBS5 (hypothesized to reflect low-fidelity DNA repair; Kim et al., 2016) nearly always increases. In contrast, in ovarian cancers, changes go up and down in similar, low proportions of tumors. Signature activity changes are generally modest in size, with the largest recorded in CLL (33%, SBS9). Some are observed across many cancer types (e.g., SBS5 and SBS40, of unknown etiology), whereas others are found in only one or a few cancer types. For example, in esophageal adenocarcinomas, SBS3 (double-strand break repair) increases, and SBS17 (etiology unknown) decreases.

**Figure S5:**
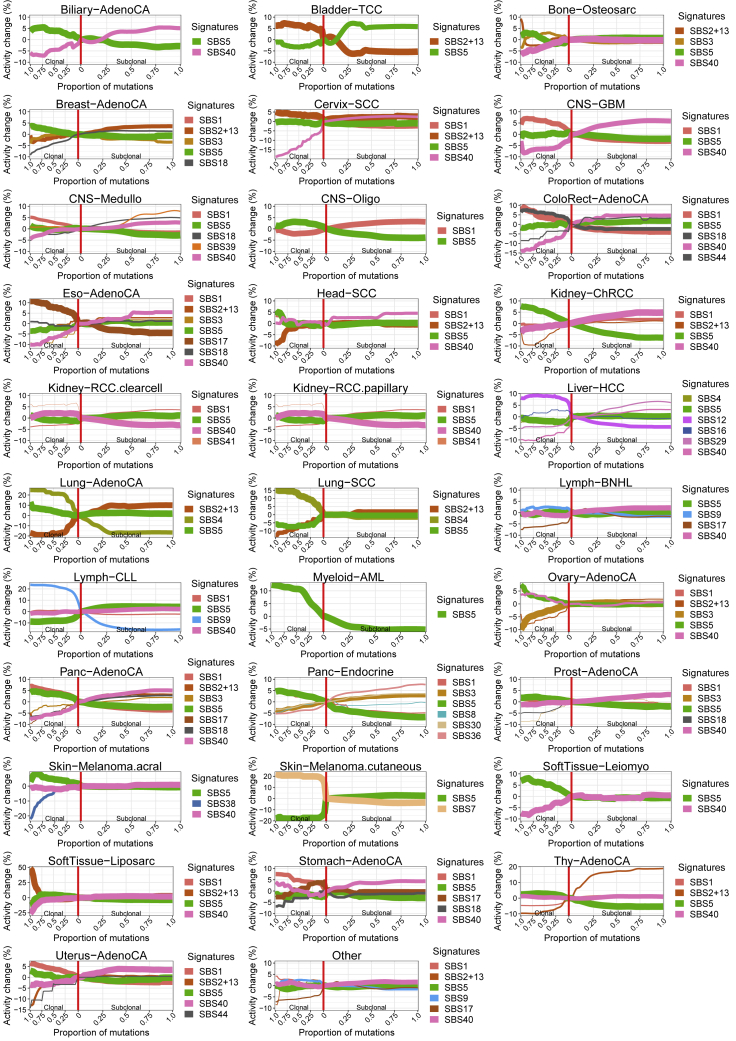
Summary signature trajectories per cancer type, related to Figure 5 The average trajectories for mutation signatures were calculated across tumors of the same cancer type. The color of the line denotes the signature and its width reflects the number of contributing tumors. The trajectories have been centered around the activity at the boundary between clonal and subclonal mutations in order to highlight relative changes in signature activity.

Signature activity changes are often monotonic as a function of CCF in individual samples (e.g., in CLL; Figure 5B) and, on average, across cancers of the same type. In other words, mutation process activities decrease or increase consistently (Figures 5C and S5). CLLs and lung adenocarcinomas exhibit a sharp change in signature activity from clonal to subclonal mutations, but activities appear stable across subclonal expansions (Figure 5C). In contrast, esophageal adenocarcinomas show a steady decrease in SBS17 activity, whereas thyroid adenocarcinomas often display a continuing increase in SBS2/13 (apolipoprotein B mRNA editing enzyme, catalytic polypeptide-like or APOBEC).

Interestingly, SBS9 activity changes in CLL and B cell non-Hodgkin lymphoma reflect the anatomical journey of B cells in their evolution to cancers. Tumors with SBS9 originate from post-germinal center B cells (Seifert et al., 2012). In these cases, SBS9 contributes to clonal but not subclonal mutations because only the tumor-founding cell was exposed to somatic hypermutation in the germinal center. Later cells in this lineage have left the germinal center and are no longer exposed to AID. Similarly, the decrease of SBS7 (UV light) activity in cutaneous melanoma suggests that these tumors have progressed to invade inner layers of the skin (Breslow, 1970), out of reach of UVB (Dupont et al., 2013). Finally, a co-occurring decrease of SBS4 (smoking) and increase of SBS2/13 (APOBEC) activity suggests that, in lung cancers, cell-intrinsic processes take over after early tumor evolution is fueled by external mutagens (Jamal-Hanjani et al., 2017).

### Mutation signature activity changes mark subclonal boundaries

If the emergence of subclones is associated with changes in mutation process activity, we expect signature change points to coincide with CCF boundaries between (sub)clones. In accordance with previous studies that highlight changes in signature activity between clonal and subclonal mutations (Gerstung et al., 2020; Jamal-Hanjani et al., 2017; McGranahan et al., 2015), we find that 34.5%–54.7% of clone-subclone boundaries and 34.5%–57.3% of subclone-subclone boundaries coincide with a signature change point (Figure 5D; STAR Methods). This not only validates our consensus clustering approach, but also demonstrates that subclonal expansions are often associated with changes in signature activity. It further suggests that increased ITH would correspond to greater activity change. Indeed, samples with the largest changes tend to be the most heterogeneous (Figure 5E). Conversely, an average 0.49 changes per sample are not within a window of subclonal boundaries (Figures 5F and 5G; STAR Methods). This suggests that some CCF clusters represent multiple subclonal lineages, consistent with our mutation phasing results above.

### The landscape of subclonal driver mutations

**Figure 6:**
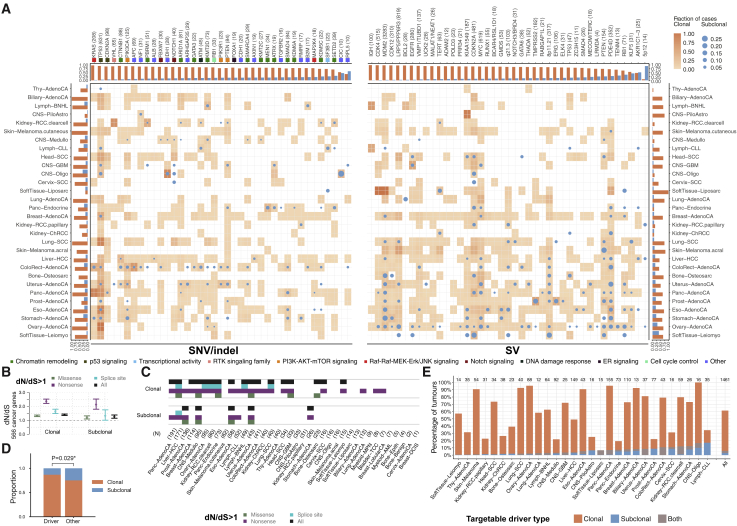
Driver mutations and subclonal selection (A) Heatmaps showing the fraction of samples of each cancer type with clonal (orange squares, transparency) and subclonal (blue circles, size) driver SNVs and indels (left) and SVs (right). Marginal bar plots represent the total fraction of clonal and subclonal driver mutations in each cancer type (side) and each driver gene or candidate region (top). Genes with 4 or more subclonal driver mutations are shown. Gene color indicates gene set and pathway annotations for SNVs and indel drivers. (B) The ratios between the fraction of mutated nonsynonymous (dN) and synonymous (dS) sites, i.e. dN/dS for clonal and subclonal SNVs in 566 established cancer genes across all primary tumors. Values for missense, nonsense, splice site, and all mutations are shown, along with the 95% confidence intervals. (C) Cancer and mutation types for which dN/dS is greater than 1 (95% confidence intervals > 1) for clonal and subclonal mutations. Cancer types are ordered by the total number of samples. (D) Proportions of (sub)clonal driver gene fusions versus non-driver fusions. (E) Survey of actionable driver mutations across cancer types, stratified by clonal status.

We leveraged the PCAWG driver annotation (Rheinbay et al., 2020) to gain insight into clonal versus subclonal driver SNVs, indels, and SVs. Of 3,933 SNV and indel driver mutations in 355 genes, we found 357 (9.0%) subclonal events in 144 distinct genes (Figure 6A). In total, 85% of samples with at least one subclone (1,381 of 1,621) contain no identified subclonal driver SNVs or indels, and only 11% of subclones (265 of 2,329) were annotated with a driver SNV or indel. In contrast, clonal driver SNVs or indels were detected in 76% of samples (1,295 of 1,705).

We next sought to examine the clonality of SV drivers (Cmero et al., 2020). We considered an SV a driver when it was associated with a region of significantly recurrent breakpoints (Rheinbay et al., 2020) at a non-fragile site (STAR Methods). By this analysis, 56.9% of analyzed samples (825 of 1,450) have a clonal SV driver, 14.7% (213 of 1,450) have at least one subclonal SV driver (Figure 6A), and 6.1% (89 of 1,450) have exclusively subclonal SV drivers.

One explanation for the dearth of subclonal driver mutations is that they have a lower population prevalence than clonal ones. Therefore, we adopted a strategy from population genetics to assess subclonal selection in the absence of driver annotations. Selective pressures acting on coding regions can be quantified using the dN/dS ratio, which compares the rates of non-synonymous and synonymous mutations (Martincorena et al., 2017). A dN/dS ratio of more than 1 indicates positive selection, whereas smaller ratios characterize negative selection, and dN/dS≈1 points toward neutral evolutionary dynamics. Analyzing clonal mutations, we confirm positive selection in a set of 566 independently established cancer genes (STAR Methods). Specifically assaying our consensus subclonal mutations for the same set of drivers, we observe a dN/dS ratio of more than 1 for nonsense, missense, and splice-site SNVs (Figure 6B). This indicates that frequent selection for driver mutations, rather than neutral evolutionary dynamics (Williams et al., 2016), shapes subclonal expansions, in agreement with our earlier study (Tarabichi et al., 2018). When considering dN/dS ratios per cancer type, only a subset shows positive selection (Figure 6C). However, these cancer types had a higher number of samples (p = 1.6 × 10^−3^, Mann-Whitney *U* test), suggesting that the absence of signal may be due to statistical power limitations.

Specific genes harbor recurrent subclonal driver SNV and indels across cancer types (Figure 6A). For example, the *SETD2* tumor suppressor is frequently mutated subclonally in clear cell renal cell carcinomas, as observed previously in multi-region sequencing experiments (Gerlinger et al., 2012), and in pancreatic neuroendocrine cancers. Interestingly, mutations in some driver genes that are exclusively clonal in most cancer types, are observed subclonally in others. For example, we find subclonal driver mutations in *MEN1* in pancreatic neuroendocrine tumors (6 of 30), *TP53* in prostate and breast cancers (4 of 12 and 5 of 59, respectively), and *CDKN2A* in pancreatic adenocarcinomas (5 of 42). Gene set analysis (STAR Methods) revealed enrichment of subclonal mutations in chromatin-remodeling genes (*e.g. ARID1A*, *PBRM1*, *KMT2C/D*, and *SETD2*), suggesting a role in subclonal variegation. Other genes often mutated in subclones are the splicing factor *SF3B1* and, in breast and pancreatic adenocarcinomas, the tumor suppressor *SMAD4*.

We similarly observed variation in SV driver clonality, implying cancer type-specific roles during tumor evolution (Figure 6A). When matched for power, 10 cancer types have a bias for clonal SV drivers (Figure 6A), suggesting that these cancers are driven by early SV events. These include SVs around *KIAA1549* in pilocytic astrocytomas, likely resulting in *BRAF-KIAA1549* fusions (Faulkner et al., 2015). Ovarian adenocarcinoma and leiomyosarcoma show the highest rates of SV driver subclonality (33.7% and 40.0%, respectively).

**Figure S6:**
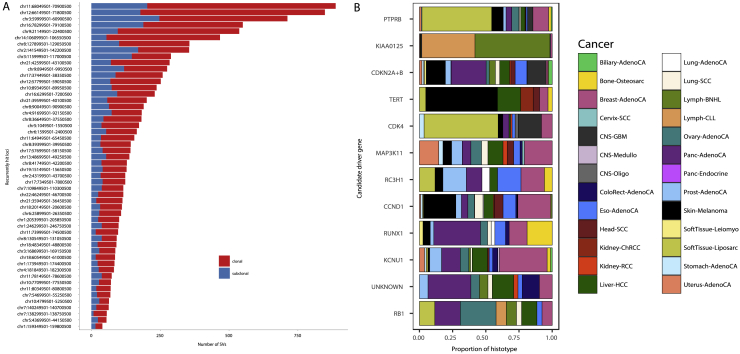
Clonality analysis of significantly recurrent breakpoints, related to Figure 6 (A) Number and clonality of SVs observed at 52 loci with significantly recurrent breakpoints (SRBs) (Rheinbay et al., 2020). SVs with a subclonal probability larger than 50% were considered subclonal and clonal otherwise. (B) Proportion of cancer types contributing to the enrichment of clonal or subclonal SVs in a locus (see Figure 6A). The genes on the y axis represent the most likely driver gene for each locus (Rheinbay et al., 2020).

Enrichment was observed for specific SV drivers across, but not within, cancer types (Figures 6A and S6). Clonally enriched SV drivers (Figure 6A; *q-value* < 0.05, rank-based permutation test) include those involving *CDK12*, *TERT*, *MDM2*, *CDKN2A*, *LRP5/PPP6R3*, *MYC*, *EGFR*, and the IGH locus. In contrast, SV drivers targeting *RB1*, *AKR1C1/2/3*, *KLF5*, and *PTEN* are enriched subclonally. Interestingly, previous studies have linked *RB1* loss to tumor progression (Bollard et al., 2017; Schneider-Stock et al., 2002; Takahira et al., 2005).

To further understand the clonality of gain-of-function driver SVs, we specifically focused on curated oncogenic fusion SVs (STAR Methods). In line with work in lung adenocarcinoma (Lee et al., 2019), we found that, across cancers, known driver fusions are more likely to be clonal than other SVs (p = 0.0284, Fisher’s exact test; Figure 6D). Some recurrent fusions appear to be almost exclusively clonal (*CCDC6*-*RET*, *BRAF*-*KIAA1549*, and *TMPRSS2-ERG*), suggesting that gain-of-function SVs occur early during tumor development.

Finally, to assess the potential effect of ITH on clinical decisions, we evaluated the clonality of actionable driver mutations (STAR Methods), reasoning that targeting subclonal mutations will likely result in treatment failure (Schmitt et al., 2016). We find that 60.1% of tumors have at least one clinically actionable event (Figure 6E). Of these, 9.7% contain at least one subclonal actionable driver, and 4.7% show only subclonal actionable events. Representing conservative lower bound estimates of tumor subclonality, our results reinforce the clinical importance of assessing the clonality of mutations.

## Discussion

We have developed consensus approaches to characterize genome-wide ITH for 38 cancer types, building on high-quality SNVs, indels, SVs, CNAs, and curated driver mutations and mutation signatures from PCAWG. Although our single-region-based results are a conservative lower-bound estimate of ITH, we detect subclonal populations in 95.1% of 1,705 tumors. Individual subclones in the same tumor frequently exhibit differential activity of mutation signatures, implying that subclonal expansions can act as witnesses of temporally and spatially changing mutation processes. Our results show rich subclonal architectures with linear and branching evolution.

Notable exceptions aside—somatic hypermutation in ancestral B cells, UVB exposure in cutaneous melanomas, and an extrinsic-to-intrinsic shift in lung cancer—we confirm that mutational processes are remarkably stable between the clone and subclones (Gerstung et al., 2020). Multi-sample efforts indeed revealed that only a subset of mutational processes are active in a given tumor, contributing genomic variation on an ongoing basis, but that shifts in their relative activities can occur over time (Bolli et al., 2018; Yates et al., 2017). Subclonal expansions, however, are often associated with changes in signature activity. In part, this may reflect the enhanced time resolution of the CCF-based subclone-subclone comparison, episodic activation of cell-intrinsic mutational processes (e.g., APOBEC; Petljak et al., 2019), or bursts of exposure to DNA-damaging mutagens (Aitken et al., 2020). Application of our methods to longitudinal and multi-sample data will yield further insights into the fine-grained temporal and spatial dynamics of mutational processes.

Analysis of dN/dS ratios revealed positive selection across subclones and cancer types. Although our analyses do not exclude the possibility that a fraction of tumors evolve under weak or no subclonal selection, they support widespread selection. Recent methodological advances using explicit growth models to quantify selection in individual tumors could shed further light on the evolutionary dynamics of tumors through single (Williams et al., 2018) and multiple (Sun et al., 2017) biopsies. A complete understanding of tumor progression will, however, need to account for evolutionary and ecological dynamics (Zahir et al., 2020). Nevertheless, our findings extend Peter Nowell’s model of clonal evolution (Nowell, 1976): as neoplastic cells proliferate under chromosomal and genetic instability, some of their daughter cells acquire mutations that convey further selective advantages, allowing them to become precursors of new subclonal lineages. Our results are consistent with a recent TRACERx study showing that selection is active late in tumor evolution, that subclonal drivers include many chromatin modifiers, and that subclonal CNAs affect large portions of the genome, including driver gene amplification (Jamal-Hanjani et al., 2017), which could explain the absence of SNV-induced drivers in those subclones.

Our observations highlight a gap in knowledge about drivers of subclonal expansions; only 11% of all subclones carry a known SNV or indel driver mutation. Late tumor development could be driven largely by different mechanisms (such as CNAs and SVs [Jamal-Hanjani et al., 2017; Mamlouk et al., 2017] or epigenetic alterations), or the pool of late driver mutations is too large to have been sampled extensively enough so far. In support of the latter, our recent study (Gerstung et al., 2020) found that late driver mutations occur in a more diverse set of genes than early drivers. The landscape of subclonal drivers in localized cancer remains largely unexplored, in part because of the limited sensitivity to call mutations at low allelic frequencies and the probabilistic nature of mutation assignment. Nonetheless, each tumor type exhibits its own characteristic patterns of subclonal SNVs, indels, SVs, and CNAs, revealing distinct evolutionary narratives. Tumor evolution does not end with the last complete clonal expansion; therefore, it is important to account for ITH and its drivers in clinical studies. It is not yet clear how drivers, selected in primary tumors, shape their metastatic potential. A few studies sequencing patient-matched primary and metastatic samples have shown evidence of selection of specific subclones harboring private driver mutations (Armenia et al., 2018; Bertucci et al., 2019; Birkbak and McGranahan, 2020; Turajlic et al., 2018; Yates et al., 2017). Additional studies are needed, assaying enough metastatic sites, taking into account the origin and the metastatic niche, as well as interrogating non-genetic modifications (Birkbak and McGranahan, 2020).

Despite a correlation in total mutation burden among different classes of somatic variation (ICGC/TCGA Pan-Cancer Analysis of Whole Genomes Consortium, 2020), tumor types deviate in their total and subclonal load of SNVs, indels, SVs, and CNAs. In addition, the processes underpinning each of these mutation types can fluctuate during tumor evolution (Gerstung et al., 2020) and induce distinct RNA alterations (Calabrese et al., 2020). CNAs are the main driver of RNA inter-tumor heterogeneity and ITH (Biswas et al., 2019; Calabrese et al., 2020) and show hallmarks of positive and negative selection (Cheng et al., 2017; Watkins et al., 2020), whereas small variants, on average, contribute most known driver mutations (ICGC/TCGA Pan-Cancer Analysis of Whole Genomes Consortium, 2020). Different tumor clones and subclones may hence walk genetic space (and its accompanying fitness landscape) in a distinct manner, to be acted upon by the evolutionary forces of selection and drift. Large-scale single-cell multi-omics efforts will, however, be required to capture the full extent and phenotypic effects of somatic variation.

Our study builds on a wealth of cancer WGS data generated under the auspices of the International Cancer Genome Consortium and The Cancer Genome Atlas, allowing detailed characterization of ITH from single tumor samples. It builds consensus CNAs from 6 methods and subclonal reconstruction from 11 methods. We found that each individual method makes errors that are corrected by the consensus. Our tools and techniques thus provide a set of best practices for future analyses of tumor WGS data. In addition, our curated subclonal reconstructions on 2,658 tumor whole genomes spanning 38 cancer types are a rich resource for future studies.

## STAR★Methods

### Key resources table

**Table:** 

REAGENT or RESOURCE	SOURCE	IDENTIFIER
**Deposited data**		

PCAWG data	ICGC/PCAWG	https://dcc.icgc.org/pcawg

**Software and algorithms**		

Consensus-bp	This paper	https://github.com/morrislab/consensus-bp
icgc_consensus_copynumber	This paper	https://github.com/sdentro/icgc_consensus_copynumber
icgc_consensus_purity	This paper	https://github.com/sdentro/icgc_consensus_purity
CICC	This paper	https://github.com/galder-max/CICC
CSR	This paper	https://github.com/wwylab/CSR
weme	This paper	https://github.com/morrislab/weme
MutationTimeR	Gerstung et al., 2020	https://github.com/gerstung-lab/MutationTimeR
icgc_consensus_clustering_assignment	This paper	https://github.com/sdentro/icgc_consensus_clustering_assignment
SimClone1000	This paper	https://github.com/galder-max/SimClone1000
SpoilSport	This paper	https://github.com/morrislab/SpoilSport
ABSOLUTE	Carter et al., 2012	https://software.broadinstitute.org/cancer/cga/absolute
Aceseq	Kleinheinz et al., 2017	https://github.com/DKFZ-ODCF/ACEseqWorkflow
Battenberg	Dentro et al., 2017; Nik-Zainal et al., 2012	https://github.com/Wedge-lab/battenberg
CloneHD	Fischer et al., 2014	https://github.com/andrej-fischer/cloneHD https://github.com/ivazquez/cloneHD-PCAWG
JabBa	Hadi et al., 2019	https://github.com/mskilab/JaBbA
Sclust	Cun et al., 2018	http://www.uni-koeln.de/med-fak/sclust/Sclust.tgz
BayClone	Sengupta et al., 2015	https://github.com/compgenome365/bayclonec/tree/compgenome365-pcawg
Ccube	Yuan et al., 2018	https://github.com/keyuan/ccube
CliP	This paper	https://github.com/wwylab/CliP
CTPsingle	Donmez et al., 2017	https://github.com/nlgndnmz/CTPsingle
DPClust	Nik-Zainal et al., 2012	https://github.com/Wedge-lab/dpclust
PhylogicNDT	Leshchiner et al., 2019	https://github.com/broadinstitute/PhylogicNDT
PhyloWGS	Deshwar et al., 2015	https://github.com/morrislab/phylowgs
PyClone	Roth et al., 2014	https://github.com/Roth-Lab/pyclone
Sclust	Cun et al., 2018	http://www.uni-koeln.de/med-fak/sclust/Sclust.tgz
SVclone	Cmero et al., 2020	https://github.com/mcmero/SVclone
RandomClone	This paper	https://github.com/sdentro/randomclone
TrackSig	Rubanova et al., 2020	https://github.com/morrislab/TrackSig

### Resource availability

#### Lead contact

Further information and requests for resources and reagents should be directed to and will be fulfilled by the Lead Contact, Peter Van Loo (peter.vanloo@crick.ac.uk).

#### Materials availability

This study did not generate new unique reagents.

#### Data and code availability

The PCAWG data are available through the ICGC portal: https://dcc.icgc.org/pcawg (ICGC/TCGA Pan-Cancer Analysis of Whole Genomes Consortium, 2020) an extensive list of resources used in this paper is provided in the key resource table. A list of all the software and code used in this paper has been put on github (https://github.com/PCAWG-11/Heterogeneity) and is provided in the key resource table. TRACERx sequencing data used in this study are described in Jamal-Hanjani et al. (2017) and Abbosh et al. (2017). Original data have been deposited to Mendeley Data: doi:https://doi.org/10.17632/by4gbgr9gd.1.

### Method details

Figures and tables referenced here have been collected in the document Methods S1.

#### Dataset

This manuscript describes analyses based on the ICGC-TCGA Pan-Cancer Analysis of Whole Genomes (PCAWG) dataset, which make use of the output from various PCAWG working groups (Alexandrov et al., 2020; Gerstung et al., 2020; Rheinbay et al., 2020; ICGC/TCGA Pan-Cancer Analysis of Whole Genomes Consortium, 2020). To aid the reading of this manuscript, we briefly describe the dataset here.

The ICGC-PCAWG dataset comprises samples selected from individual ICGC and TCGA projects for which completion was imminent in 2015, as detailed in ICGC/TCGA Pan-Cancer Analysis of Whole Genomes Consortium, 2020. Donors were included if a tumor and matched normal were sequenced to a minimum per-base coverage of 30x and 25x respectively, on the Illumina HiSeq platform with 100-150 bp paired-end reads. 2,834 donors passed these criteria, which were reduced to 2,658 after an extensive quality control procedure (also described in IICGC/TCGA Pan-Cancer Analysis of Whole Genomes Consortium, 2020). In total, 2,778 cancer samples from these 2,658 distinct donors were included in the final dataset, comprising 2,605 primary tumors and 173 metastases or recurrences. Purity and ploidy values (Figure 1 in Methods S1) as well as whole genome duplication status (Figure 2 in Methods S1), were estimated via a consensus approach based on 6 CNA callers, as described further below.

All sequencing data was collected and analyzed through a series of standardized primary analysis pipelines to first realign reads to the same reference genome and subsequently call SNVs, indels and SVs, using a homogenized procedure, as is detailed in ICGC/TCGA Pan-Cancer Analysis of Whole Genomes Consortium, 2020). High quality somatic SNV and indel calls were established via a robust consensus strategy based on multiple methods to achieve greater accuracy. Eighteen different callers were considered and asked to produce calls on 63 tumors selected across 23 cancer types and 26 contributing projects. For 50 tumors, there was sufficient DNA to perform deep sequencing via DNA hybridization capture. Around 250,000 SNVs and indels were selected, stratified by the number of methods by which they were called, followed by uniform sampling across the overlaps. A consensus approach was then defined to maximize precision and sensitivity, based on the three core pipelines supplied by the Broad, DKFZ-EMBL and Sanger. Two additional callers (one SNV caller from MD-Anderson and an additional indel caller from the Barcelona Supercomputing Center) were added to improve the ability to detect low-allele-frequency variants. Variant allele frequency was considered when sampling variants for the validation; the precision obtained on SNVs at different allele frequencies is shown in Figure 3 in Methods S1.

Driver calls have been made available throughout the PCAWG project; the full findings and methods are described in Rheinbay et al. (2020). Somatic driver SNVs and indels were discovered by combining the outputs from 16 different discovery methods, including factors such as mutational burden, functional impact and mutation hotspots. To combine calls, their approach integrated p values assigned to each event by the 16 callers, considering autocorrelation between methods based on similar principles, and applying multiple-testing correction. This was applied to protein-coding genes, promoters, untranslated regions (UTRs), distal enhancers and non-coding RNAs.

Mutational signatures, and estimates of their activity in each sample, have been produced by the PCAWG signatures group, with findings and methods detailed in Alexandrov et al. (2020). They applied two different computational approaches based on non-negative matrix factorization to 4,645 genome and 19,184 exome sequences, to extract 49 single base substitution, 11 doublet base substitution and 17 indel signatures. The doublet base and indel signatures are completely novel, while the single base signatures are broadly concordant with the published signatures in COSMIC, with some additional signatures, and some signatures now being split. The authors report a high concordance between the two approaches on all PCAWG samples, except in the case of hyper-mutators (5.6% of samples). Both reference sets of signatures and the per-sample quantifications of activity have been made available to the PCAWG project. In this manuscript, for simplicity we make use of the signatures output from one of the approaches only (SigProfiler).

#### Copy number consensus

##### Overview

The copy number consensus procedure builds on top of the output from six different copy number callers. Upon inspection of profiles produced by the individual callers, we observed that profiles differ depending on their segmentation and that disagreement on copy number states of large proportions of the genome is the result of disagreement on whether a whole genome duplication had occurred. We therefore first constructed a complete set of breakpoints from an initial pass of five of the six methods (the JaBbA output was not yet available). The consensus breakpoints were then fed back into the methods to produce copy number calls from all six methods. After resolving ploidy disagreements, we applied a procedure to every consensus segment that looks for agreement in major and minor allele states between the output of the six callers. Finally, we produced consensus purity calls for every tumor by combining purity calls from the six copy number callers with those from subclonal architecture reconstruction methods that produce purity calls from SNV data. The full procedure results in a purity estimate for every tumor in the PCAWG dataset and a copy number call for every consensus segment, where every call is assigned a confidence level and a quality star.

##### ABSOLUTE

Ignaty Leshchiner, Dimitri Livitz, Gad Getz

We used the ABSOLUTE algorithm to calculate the purity, ploidy, and absolute DNA copy number of each sample (Carter et al., 2012). Fragment based coverage (derived from full read template span) was collected over the genome and corrected for GC and mappability biases. Tangent-normalization of the tumor copy number profile was performed by using a panel built from PCAWG normal samples. Further, allele-specific copy number was computed based on heterozygous sites identified in the paired normal and segmentation was performed by using a circular binary segmentation algorithm. The Nelder–Mead algorithm was used to search the space of possible purity and ploidy solutions and prioritize them. Dirichlet Process clustering was performed on subclonal copy number segments, to annotate identical subclonal copy-number cluster states. Somatic SNVs were imputed on the computed absolute copy number profile and the cancer cell fraction and multiplicities were inferred for each mutation independently prior to running subclonal architecture reconstruction.

##### ACEseq

Kortine Kleinheinz, Roland Eils, Matthias Schlesner

ACEseq (allele-specific copy number estimation from sequencing) (Kleinheinz et al., 2017) determines absolute copy number from a combination of the coverage ratio of tumor and matched normal in genomic windows and the B-allele frequencies (BAF) of the corresponding SNPs. In addition to the copy number, tumor ploidy and tumor cell content are estimated.

During pre-processing of the data, allele frequencies are obtained for all single nucleotide polymorphism (SNP) positions recorded in dbSNP version 135 (Levy et al., 2015). To improve sensitivity for the detection of genomic imbalances, SNPs in the matched normal are phased with IMPUTE2 (Howie et al., 2009). The coverage for 10 kb windows with sufficient mapping quality and read depth (maximum mappability over at least 50% of the window; average coverage of at least 5 reads per base) is determined and corrected for GC content-dependent as well as replication timing-dependent coverage bias. Subsequently the genome is segmented with the PSCBS package (Olshen et al., 2011) into regions of equal coverage and imbalance state. Prior to segmentation, structural variant (SV) breakpoints defined by the consensus SV set were incorporated as segment borders. The resulting segments were submitted for consensus breakpoint estimation.

The segments obtained with the final consensus breakpoints were annotated with coverage and BAF values to estimate the tumor cell content and ploidy of the sample. All ploidy / tumor cell content combinations in the range of 1 and 6.5 for ploidy and 15% and 100% for tumor cell content were modeled, and the combination which best explains the data was chosen. Balanced segments were constrained to even-numbered copy number states during the fitting, and solutions which required a BAF value > 1 or negative copy numbers for at least one segment were excluded.

##### Battenberg

Stefan Dentro, Kevin Dawson, Henry Lee-Six, David Wedge, Peter Van Loo

We applied the previously described (Dentro et al., 2017; Nik-Zainal et al., 2012) Battenberg algorithm to the PCAWG data. Briefly, the Battenberg pipeline starts with collecting read count information for all 1000 genomes phase 3 SNPs from the tumor and its matched normal. B-allele frequency (BAF) and relative coverage ratio (logR) are calculated for each SNP, after which the logR is corrected for GC content. The matched normal is used to obtain germline heterozygous SNPs, which are subsequently phased using IMPUTE2 to obtain haplotype blocks. Outlier blocks are switched because two consecutive haplotype blocks can have a different allele chosen as allele *b*. The phased SNPs result in precise BAF estimates that allow for detection of subclonal copy number.

The data is segmented using piecewise constant fitting (PCF), with structural variants (SVs) taken in as prior established breakpoints. A clonal copy number profile is fit by a grid search over purity and ploidy combinations. The purity/ploidy combination that yields the largest proportion of the genome with clonal copy number is picked. Subclonal copy number is fit by first testing whether the BAF is significantly different from the currently fit clonal copy number state, allowing for at most a 1% deviation from the clonal BAF. If the BAF is significantly different, then there are four options under the assumption that there are at most two cellular populations with only a single allele copy number difference: Allele A and B are rounded up, A and B are rounded down, A is rounded up and B is rounded down, or A is rounded down and B is rounded up. The option that explains the observed BAF best is picked and used to fit the final copy number states.

The algorithm used for this analysis is different from the original version in three ways: Correction for GC content, inclusion of SVs during segmentation and merging of adjacent segments that are assigned the same copy number states. Sequencing data can be affected by inconsistent coverage that correlates with GC content (Benjamini and Speed, 2012) and coverage is contained within the LogR calculations. A correction step is therefore required. We correct the LogR by fitting a linear model that explains the data as being affected by two types of GC artifacts: A high frequency wave, encoded in a small window size and a low frequency wave corresponding to a large window size. We pre-calculated the GC content in various windows centered on each 1000 genomes SNP. For each window size we calculate the GC content correlation and pick the highest correlating window size below 1MB and of 1MB or larger. The residuals of the linear model are saved as the corrected LogR.

Inclusion of SVs during segmentation increases the accuracy of the segmentation and therefore the copy number calls. We first create SV segments by sorting the breakpoints by chromosome and position. Then, for every SV segment, PCF is run separately to obtain the final segmentation. After copy number has been established, we merge adjacent segments with equal copy number states. This step removes SV supported segments that do not constitute a copy number change (inversions).

##### cloneHD

Ignacio Vázquez-García, Ville Mustonen

We used the cloneHD algorithm for copy number calling (Fischer et al., 2014). Briefly, cloneHD implements a Hidden Markov Model (HMM) that describes the (hidden) copy number state of the sample. The hidden states emit observations that correspond to the read depth and B-allele frequency counts (BAF) of each sample. The emission models implemented in cloneHD are Poisson for read depth and Binomial for BAF, and their over-dispersed counterparts. Here we used cloneHD in read depth + BAF mode and exclusively the Poisson and Binomial emission models.

The first step in the cloneHD pipeline is the filterHD algorithm, which is a continuous state space HMM with a jump-diffusion state transition model. The diffusion component is useful to allow for the hidden state to change smoothly and models sources of (non-biological) bias. The jump component corresponds to a discrete change in the hidden state above the noise level, such as a copy number gain or loss. The result of filterHD is a posterior probability of the hidden state, e.g., the posterior read depth (both for the tumor and its matched normal) or the posterior B-allele frequency for BAF, and per-locus jump probabilities. Importantly, filterHD does not seek to explain the subclonal structure in the data and is a generic algorithm for fuzzy segmentation. It plays a similar role to the segmentation step carried out by most copy number callers.

To find out a biologically interpretable model of the data we used cloneHD. This was done by applying cloneHD to both read depth and BAF data of each sample, the per-locus jump probability for these, and the mean of the posterior read depth for read depth of the matched normal sample (to correct for bias). The HMM implemented in cloneHD aims to explain the data as a mixture of copy number states weighted by the corresponding cellular fractions of normal and cancerous cells, while allowing for the hidden state to change using the per-locus jump probability. Copy number calling using single samples from a tumor suffers from degenerate solutions that can be difficult to resolve. Most importantly, a correct baseline ploidy of the sample has to be inferred. cloneHD allows the user to specify a penalty for leaving a normal baseline (diploid) copy number. A penalty value of 1.0 does not penalize leaving the normal baseline. Without this penalty, higher copy number baselines are typically preferred over lower ones. Biologically, this would mean a higher number of inferred whole genome duplications. By default, we used 0.95 for this penalty. This setting was used to provide cloneHD copy number calls for the consensus breakpoint calling. After consensus breakpoints were available, we used them to obtain per-locus jump probabilities, allowing for transitions only at these set locations. We then executed cloneHD as before, using the baseline penalty values 0.80, 0.95 and 0.99. Based on the consensus average ploidy for each sample we then selected the closest cloneHD solution. We note that for most samples all three solutions are essentially the same, however, for a subset the penalty is decisive whether to call a whole genome duplication or not.

##### JaBbA

Xiaotong Yao, Steven Schumacher, Rameen Beroukhim, Marcin Imielinski

We used Junction Balance Analysis (JaBbA) to integrate paired-end and read depth signals and infer copy numbers on genomic intervals and rearrangement junctions (Hadi et al., 2019).

JaBbA exploits the principle that copy number alterations (CNA) always result from rearrangements or whole chromosome gains / losses, previously explored in several publications on germline and cancer genome structural variant analysis (Li et al., 2016; Medvedev et al., 2010; Oesper et al., 2012). This results in a junction balance constraint (JBC), namely that the copy number of every genomic segment is consistent with its neighbors. Applied to a genomic graph whose nodes represent (+ and – strands of) genomic segments and edges represent alternate and reference junctions (corresponding to 3-5′ phospho-diester bonds), the JBC forces the copy number of every node to equal the sum of the copy numbers of all incoming (similarly, outgoing) edges.

To fit this model to data, we formulate a mixed integer quadratic program (MIQP) to match node copy numbers with estimates of read depth measurements parameterized as posterior means and variances at *n* genomic segments. The summed deviation of the model fits (i.e., segment copy numbers) from the observed data (mean estimate on segment read depths) is captured in a weighted sum of squares quadratic objective function that incorporates purity, ploidy, and inverse variances as weights. To allow for missing junctions, which frequently result from rearrangements occurring at hard to map genomic regions (e.g., centromeres) and force local violations of junction balance, we add a slack parameter to the JBC. These slack parameters represent “loose ends” in the structural variation model, which can either represent missing junctions or “biological loose ends” that could correspond to neo-telomeres in the cancer karyotype. We then add a linear slack penalty to the objective function, which penalizes the number of slack (loose end copies). We provide a user-defined hyper-parameter, which balances the contribution of (linear) slack and quadratic (read-depth) penalties to the analysis. Model fitting yields estimated integer value copy numbers on graph nodes, edges, and loose ends and numeric value purity / ploidy.

For the PCAWG consensus copy number analysis, we applied JaBbA in two rounds, in concordance with the other copy number methods. In practice, JaBbA begins with a .bam file, a junction call set (e.g., a BND VCF file, a .bedpe), and (optionally) a preliminary segmentation and purity / ploidy input. We compute high density binned read depth (200bp bins) by calculating tumor / normal ratios of GC and mappability corrected coverage of “proper” read pair centroids in the .bam file (fragCounter). In the absence of a preliminary segmentation (pre-consensus run), we use circular binary segmentation (CBS) to segment high-density binned coverage (∼15M intervals) into a lower dimensional collection (e.g., 10-100K regions) of regions of constant copy number. In the subsequent “consensus” run, we use the “initial copy number consensus” as the preliminary segmentation. For the junction input, we used the full PCAWG-6 call set during the pre-consensus run. In subsequent runs, we used a strict intersection of this junction set with the end points of consensus PCAWG-11 segmentation.

The union of the endpoints in this segmentation and the breakpoints in the junction call set induce a partition of the reference genome, from which we build a genomic graph of *n* segments and *m* edges. We use the high-density coverage to compute posterior means and variances on fragment density at each of these *n* segments. We solve a preliminary least-squares problem (corresponding to our full MIQP objective function, but without JBC, i.e., with zero slack penalty) to infer an affine transformation between read depth and integer copy number, and hence purity and ploidy. This least-squares inference implements principles described in by Van Loo et al. (2010). We find that in practice, the JBC does not alter purity and ploidy estimation, and dividing the computation into these two phases dramatically improves convergence and speed of the MIQP. Following purity and ploidy inference, application of the full MIQP using CPLEX (http://www.ibm.com//www.ibm.com/products/category/business/commerce) fits *n* nodes and *m* edges in the graph with integer copy numbers. After fitting integer (total) copy numbers, JaBbA uses allelic read counts at germline heterozygotic sites (obtained via samtools pileup at HapMap v3 sites) to infer likely allelic copy numbers at genomic segments. See the pipeline in Figure 4 in Methods S1.

##### Sclust

Martin Peifer, Yupeng Cun, Tsun-Po Yang

The copy number module of Sclust has extensively been used in our recent large-scale sequencing efforts of small cell lung cancer and neuroblastoma (Cun et al., 2018; George et al., 2015; Peifer et al., 2015). In total, Sclust performs copy number segmentation and determines purity, ploidy, and allele-specific copy number (both clonal and subclonal). As input, the method requires read counts from tumor and matched normal sample in a sufficiently partitioned genome. Within the ICGC pan-cancer analysis, we chose a partitioning into non-overlapping 1kb windows. The read counts are then used to generate GC-corrected read ratios between the tumor and the normal. Second, Sclust requires base counts of common single nucleotide polymorphisms (SNPs) in both the tumor and the matched normal sample. This data is used to compute B allele frequencies at all heterozygous sites of the normal. Given this as input data, the algorithm first preforms segmentation based on the read ratios by detecting significant jumps in the data. Purity and ploidy estimates are then computed using a conditional maximum likelihood approach. In particular, the likelihood of B allele frequencies is optimized to obtain purity estimates for a fixed expected ploidy. This yields purity estimates in function of the expected ploidy, which is plugged into the likelihood of read ratios and optimized in order to obtain an estimate of the expected ploidy. Here, the search domain can be adjusted to select a more suitable solution in case of whole genome duplications. Along the optimization, allele-specific copy numbers are computed. To derive subclonal copy number changes, we test which of the B allele frequencies disagree with the model predictions (here a significance threshold of 1% is chosen). For all segments assigned to be subclonal, we select the best combination of copy number states that are just one copy apart. Finally, ploidy estimates are computed directly from the determined copy number states.

#### Copy number consensus approach

##### Consensus copy number procedure

ICGC PCAWG relied on a consensus strategy for SNVs, SVs, and indels, as calls for each on which different algorithms agreed were understood to be high-confidence predictions. For copy number calls, we relied on a similar consensus approach, which combined results from six individual copy number callers: ABSOLUTE, ACEseq, Battenberg, CloneHD, JaBbA and Sclust.

Each copy number caller uses a two-step process, first segmenting the genome into regions assumed to have constant copy number state, then determining the clonal and subclonal copy number states of each segment. Disagreement among copy number callers arises primarily from two factors: differences in genome segmentation, and uncertainty concerning whether a whole-genome duplication (WGD) occurred. Thus, our consensus strategy resolved both factors for each sample, allowing us to determine a consensus copy number state for much of the genome across samples.

##### Copy-number-calling methods differ in genome segmentation

Jeff Wintersinger, Quaid D. Morris

Copy number callers segment a sample’s genome into regions assumed to have constant copy number. To delineate these segments, they find breakpoints at which the boundaries between segments where copy number state changes. Once these breakpoints are established, callers then determine the mixture of copy number states within each segment, encompassing the number of major allele copies, the number of minor allele copies, and the proportion of cells with that state.

Copy number methods differed substantially in the number of breakpoints they defined through their genome segmentations (Figure 5 in Methods S1), with some methods calling an order of magnitude more breakpoints than others. Broadly speaking, these can be broken into two classes: “liberal” methods (ACEseq and cloneHD) called, on average, a great many more breakpoints than “conservative” methods (ABSOLUTE, Battenberg, JaBbA, and Sclust). To resolve this disagreement between different algorithms concerning the proper genome segmentation, we established a set of consensus breakpoints, taking into account this “liberal” versus “conservative” distinction. All methods subsequently used that consensus segmentation in determining copy number states.

##### Method for determining consensus segment breakpoints

Jeff Wintersinger, Quaid D. Morris

As the six copy-number-calling methods differed substantially in the segmentation they calculated, we increased agreement in our consensus copy number calls by deciding upon a set of consensus breakpoints for each genome. We developed a consensus strategy that favored “true positive” breakpoints at the potential cost of increasing “false negatives” to create a complete set of breakpoints. Orthogonal evidence of copy number breakpoints from structural variants was used to quantify the “true positive” and “false negative” rate of our consensus approach (further detailed below). Copy number methods were required to take the consensus segmentation as input, but were permitted to merge adjacent segments they judged as having the same copy number. They were however not allowed to introduce additional breakpoints that would create new segments. The cost of introducing spurious breakpoints was therefore less than that of missing breakpoints where the underlying copy number state did indeed change.

The algorithm we developed for determining consensus breakpoints draws on the insight that regions between adjacent segments indicate a method’s uncertainty in precisely where the breakpoint delineating change in copy number state should lie. The segmentation released by each method consists of a set of regions defined by the genomic loci *S*_*i*_ and *E*_*i*,_ indicating the start and end of each region, with the interval (*S*_*i*_, *E*_*i*_) representing a region of constant copy number. On a given chromosome, however, we need not have each region immediately following its predecessor such that there is no gap, which would imply *S*_*i*_ = *E*_*i*-1_ + 1. Instead, the region (*E*_*i*-1_, *S*_*i*_) has undefined copy number—the segmentation method inferred that CN status changed at some point within this interval, but could not pinpoint the location because of the noisy signal.

Our consensus breakpoint algorithm leverages this information concerning uncertainty (Figure 6 in Methods S1).

1. For each copy number segmentation method *M*_*s*_, take each reported segment (*S*_*i*,_ *E*_*i*_), and generate an interval spanning (*E*_*i*_ - δ, *S*_*i*+1_ + δ). This interval represents a plausible region over which a breakpoint may lie according to *M*_*s*_, permitting the breakpoint to move δ bases upstream or downstream beyond the reported boundaries. Here, we set δ = 50 kb, which we selected after comparing the breakpoints generated by a range of δ values to the underlying read depth and B allele frequency signals in the data. δ = 50 kb achieved a reasonable balance between false-positive consensus breakpoints (when δ was too large) and false-negative consensus breakpoints (when δ was too small).
2. Compute the intersection of intervals between each method. Scanning from the start of the chromosome, find the first intersection *I*_*s*_ supported by the threshold method set *T.* Here, we defined *T* to be any combination of at least three of the six copy number methods, or any combination of two of the “conservative” methods (i.e., ABSOLUTE, Battenberg, JaBbA, and Sclust). We called this strategy *any3_any2_conservative.* This avoided calling consensus breakpoints supported by only the two “liberal” methods (ACEseq and cloneHD), while correcting false-negative cases where a breakpoint was supported by only two of the input methods despite clear evidence in the underlying data. Relative to *any3*, a stricter criterion requiring that at least three of six methods support a breakpoint, *any3_any2_conservative* added only a small number of breakpoints (Figure 7 in Methods S1).
3. Select the start and end interval loci from each method *m* falling within the interval, corresponding to *S*_*m*_ and *E*_*m*_, respectively. Score each locus according to the size of the associated gap *G*_*m*_ = rank(*S*_*m*_ - *E*_*m*_), where *G*_*m*_ corresponds to the rank in the empirical cumulative distribution of all gaps generated by the given method. Thus, if a method assigns a large gap between two segments relative to the other segments it generates, its uncertainty in breakpoint placement is understood to be relatively large; conversely, a relatively small gap indicates high certainty. Select the breakpoint from within the intersection that has the smallest *G*_*m*_ value. In the case that two breakpoints in the intersection have the same *G*_*m*_ (which occurs, e.g., because both *E*_*m*_ and *S*_*m*_ fell in the intersection), arbitrarily prefer end loci to start loci. Record this as the single consensus breakpoint associated with this intersection. Otherwise, in the rare case that no input start and end loci fall in the intersection, report the upstream-most end of the intersection as the consensus breakpoint. Such cases arise when only the δ bases padding each input segment intersect, meaning that the intersection as a whole is relatively small, and that either end of the intersection can be taken as a reasonable representation of a breakpoint’s position.
4. Remove all intervals that contributed to the intersection. Return to step 2. Repeat until no intersections passing the threshold remain on the chromosome.
5. Add PCAWG consensus structural variants to the consensus breakpoint set. To do so, find all consensus breakpoints within 100 kb of a consensus SV. Replace the consensus BP with the consensus SV, as the SV presumably represents the same mutational event, but with greater precision concerning position. For any SVs lacking a consensus BP within 100 kb, add the SV as an additional consensus breakpoint.
6. Add breakpoints at centromeres and telomeres as necessary, as copy number states cannot be called across these boundaries. Use the chromosome lengths and centromere start and end locations reported in the hg19 human reference genome. If any centromere start or end lacks a consensus breakpoint within 1 Mb, add an additional consensus BP at that location. If a consensus breakpoint occurs within the centromere, move it to the start or end of the centromere, according to whichever point is closer. Likewise, if no breakpoint occurs within 1 Mb of a chromosome start or end position (representing telomere locations), add an additional breakpoint at the chromosome start or end.

##### Most consensus breakpoints obtain support from SVs

Every copy number event should generate associated structural variants (barring whole chromosome events or breakpoints that fall within regions where difficulties in the alignment of sequencing reads hinder SV calling, like centromeres). As such, each breakpoint should have associated with it a nearby structural variant. On a per-tumor basis, we compared the number of consensus breakpoints with support from the PCAWG consensus SVs, to the number of consensus breakpoints lacking such support (Figure 8 in Methods S1). Under the *any3_any2_conservative* strategy, 80% of cancers had more SV-supported than SV-unsupported breakpoints, which helped validate our consensus threshold. In examining the proportion of breakpoints with SV support as a function of number of breakpoints, we found that the average cancer received SV support for 77% of its breakpoints (Figure 9A in Methods S1), with a slight positive correlation between total number of breakpoints and the proportion finding SV support. Furthermore, in each tumor, 83% of consensus SVs supported nearby consensus breakpoints (Figure 9B in Methods S1), with no correlation between number of SVs and the fraction that supported breakpoints.

In theory, all non-centromeric, non-telomeric copy number events should have associated structural variants, but not all structural variants should correspond to copy number changes (e.g., balanced translocations do not affect copy number). Consequently, finding on average SV support for 77% of our consensus breakpoints assured us that our consensus breakpoints were largely recapitulating the breakpoints found by orthogonal structural variant detection algorithms, increasing confidence in our results. Manual inspection of consensus breakpoints without associated consensus SVs suggested that there was sufficient evidence in the underlying B allele frequency and read depth signals to call the breakpoints, implying that simply taking the set of consensus SVs as our breakpoints would have missed legitimate copy number events. Given our preference for false positives over false negatives, the non-SV-supported breakpoints were a worthwhile addition to our breakpoint set, even if some were false.

##### Resolving ploidy disagreement between copy number callers

Jeff Wintersinger, Stefan Dentro, Ignaty Leshchiner, Kortine Kleinheinz

After the six copy number callers produced copy number profiles on the provided consensus segmentation, we focused on disagreement between the callers on ploidy calls. An automated procedure was used to identify these tumors, which were subsequently subjected to a rigorous review procedure with the aim to resolve the discrepancy.

##### Finding outlier profiles

Jeff Wintersinger

CNA callers often disagreed on whether cancers had undergone whole-genome duplications (WGDs). To determine which cancers suffered from this disagreement, we ran all CNA callers across the full dataset, then declared each cancer “WGD-certain” if all callers agreed on whether it was diploid or tetraploid, and “WGD-uncertain” otherwise. We performed this characterization as follows:

1. For each caller, compute the proportion of the genome with a clonal diploid copy-number state of (major allele, minor allele) = (1, 1). Also compute the proportion in clonal tetraploid (2, 2). If either proportion exceeded 20% of the genome, declare the caller’s result on that cancer “diploid” or “tetraploid,” respectively. Otherwise, if neither state accounted for at least 20% of the genome, report the caller’s result as “unknown.”
2. If all callers agreed on “diploid” or “tetraploid,” declare the cancer as “WGD-certain,” meaning that the callers agree it is diploid (no WGD occurred) or tetraploid (a WGD occurred).
3. Otherwise, if any callers disagree with the others on “diploid” or “tetraploid,” or if any caller reports “unknown,” declare the cancer as “WGD-uncertain.”

After this procedure, 461 of 2778 cancers were deemed WGD-uncertain.

A preliminary consensus copy number procedure revealed an additional 315 tumors with agreement on less than 20% of the genome between the CNA callers. These 315 cases were also reviewed.

##### Resolving whole genome duplication uncertainty

Ignaty Leshchiner, Stefan Dentro, Kortine Kleinheinz

The above described copy number reconstruction methods differ in how copy number is estimated and perform different pre-processing steps. It was therefore expected that the methods could deviate on the called profiles. The samples identified through the above procedure have been put through a review by a panel of experts. Initially to understand where the discrepancy lay between the profiles, and later to resolve the differences. The expert panel consisted of three core and five alternating members and sat down for four afternoons. Each member prepared a figure per sample with all possibly interesting information (raw data, SNV multiplicity states, subclonal architecture calls, etc.). A central figure (see Figures 10 and 11 in Methods S1 for examples) was used to feed the discussion that contained: Copy number profiles from all methods and raw BAF, copy ratio (LogR) and multiplicity values from ABSOLUTE. During the review, a sample was marked as *WGD* or *no_WGD*, and it was only marked on unanimous agreement among the panel. When the panel could not agree the sample was marked as *unknown*. The panel operated on the basis that sufficient evidence of a genome doubling is required to mark a tumor as *WGD*.

Key identifiers of a missed whole genome doubling that were considered: Subclonal copy number called at 0.5 CCF (these segments become clonal when doubling), a SNV cluster at 0.5 CCF (these mutations become clonal when doubling the ploidy), there should be a clonal SNV cluster at around 1.0 CCF and no superclonal clusters beyond 1.0 CCF, while very large homozygous deletions in general should not occur (whole chromosome(s), or whole chromosome arm(s), in general we worked with a threshold of 20Mb, which is often just visible on whole genome figures). Finally, when the tumor is called as genome doubled one expects some SNVs on one chromosome copy, except in the unlikely scenario that the whole genome duplication was the last event to occur in the tumor before resection and it became clonal without the tumor acquiring further SNVs.

Over time, we observed recurrent scenario’s, which allowed for quick identification of the outlier. For example, one method did not allow for a purity below 0.3. It would call a higher purity, pushing the copy number profile upward and leaving a purity/ploidy discrepancy that does not allow clean inference of the subclonal architecture inference from SNV data. Another example are samples with very heavy wave artifacts in the coverage. In this case, some methods would over-segment the genome to fit the noise, leading to a discrepant ploidy that our automated approach could not resolve (see Figure 11 in Methods S1). Methods have been adapted since the review to address the scenarios uncovered.

The panel did not agree on the WGD status of 38 cases, due to the high complexity of their copy number profiles. All segments of these genomes have been marked with level *i* accordingly (see further).

##### Consensus copy number calls

Stefan Dentro, David Wedge, Peter Van Loo

With a consensus segmentation established and whole genome duplication uncertainty resolved we could build the consensus copy number profiles. We aimed for every profile to contain a call for every segment where there are reliable calls. To do so we first identified 6 ways of extracting agreement between the CNA callers:

a. All methods agree on a clonal copy number call (both major and minor alleles).
b. A single method disagrees on the copy number state of a single segment, leaving the call out creates agreement.
c. A single method disagrees on the ploidy of a sample, leaving the profile out creates agreement.
d. The majority of available methods agree on clonal copy number.
e. Complete or leave-one-out agreement may be achieved by rounding subclonal copy number.
f. Majority vote may be achieved after rounding subclonal copy numbers.

For each sample, every segment goes through the list starting at *a*, until agreement is reached. On average, we obtain consensus on 93% (median 95%) of the genome after reaching level *f*. The segments that remain without a consensus call go through a second approach that is designed to find a call from a single method.

We first calculate, for every CNA method, what proportion of the consensus profile it agrees with after reaching level *f*. This allows ranking of the methods, where an overruled profile due to ploidy is not included (see filtering below). We then devised the following additional levels:

g. Take the call from the best method. If there is consensus for the copy number state of one of the alleles we require the best method to agree with it (see rounding below).
h. Take the call from another method, iterating from the best to the worst performing method.

A special level was added to distinguish samples where the expert panel did not reach consensus on the ploidy of a sample. These cases went through the full consensus procedure, but afterward all their segments were re-marked as level *i*.

We then devised a star rating system that denotes the amount of confidence in each of the calls. Levels *a*, *b* and *c* are the strictest and require all methods but one to agree, at the least. These segments are therefore assigned 3 stars. Segments for which a majority of the methods agree on either clonal or rounded clonal copy number are assigned 2 stars (levels *d*, *e*, *f*). The remaining levels (*g*, *h*, *i*) receive 1 star to denote the lowest confidence.

##### Rounding subclonal copy number

Subclonal copy number is reported in three different ways across the 6 methods. ABSOLUTE reports up to 3 different copy number states (a combination of a major and a minor allele) per segment, of which 1 is termed the ancestral state. Battenberg and Sclust report subclonal copy number as a mixture of two states, while ACEseq returns a single non-integer state (i.e., a mixture). Both cloneHD and JaBbA provided clonal calls only.

When subclonal copy number is reported as individual states found in proportions of cells (ABSOLUTE, Battenberg and Sclust), we selected the reported states individually. For ABSOLUTE that corresponds to selection of the copy number states corresponding to the ancestral state, the highest CCF state and the lowest CCF state separately. For Battenberg and Sclust that meant selecting the states from the highest CCF and lowest CCF calls separately. As ACEseq reports two non-integer states we opted for rounding both alleles up and down.

To create a consensus call, we first obtain an inventory of the available copy number states across all possible rounded states, including the clonal calls from cloneHD and JaBbA. If there is a major/minor allele combination that satisfies the minimum number of methods criterion (either leave-one-out or majority vote) we select that state as the consensus.

If no agreement is reached we attempt to establish consensus by voting for the major and minor allele separately. An allele is accepted if it passes the minimum number of methods threshold. In some cases this leads to consensus on one of the alleles. The state of that allele is saved and fed into levels *g* and *h*, where a call is selected where one of the alleles agrees with the established consensus allele.

##### Chromosomes X and Y

Fewer methods report on X and Y chromosomes:

• X: ABSOLUTE, Battenberg (females), ACEseq, cloneHD and JaBbA
• Y: ABSOLUTE, ACEseq and JaBbA

The number of methods required to agree for the separate levels is adjusted accordingly.

##### Consensus purity

For consensus purity, we have calls from the 6 CNA methods and a number of SNV-based methods: CliP, CTPsingle, PhyloWGS, cloneHD (on SNVs) and Ccube (discussed further). Outlier calls are first removed for CNA and SNV methods separately (see filtering below). For each sample, we establish a density over the combined data. Analogous to taking the mode, we select the call that is closest to the highest peak in the density as the consensus.

There is a larger discrepancy in purity calls from CNA methods on samples with few copy number alterations. For tumors where less than 8% of the genome is altered, we therefore calculate the density over the calls from SNV based methods only.

Finally, the median absolute deviation is calculated to capture the amount of agreement between the calls and is used as a measure of confidence.

##### Filtering

After the expert panel review of ploidy-uncertain cases, a reference ploidy can be obtained for almost all samples. The methods either all agreed on large portions of the genome or certain ploidy estimates were overruled by the expert panel. We therefore calculated a reference ploidy that serves to overrule calls from individual CNA callers. To allow for larger variations on high ploidy profiles we set the threshold at 0.25 times the reference ploidy. If a profile differed by more than this threshold, it was automatically overruled and excluded from the procedure for both copy number and purity.

Further filtering on the purity calls was performed to remove outliers. A purity call was filtered out if it differed from all other non-ploidy-overruled purity calls by more than 0.2. This method was applied separately to SNV-based purity values.

We also excluded calls on complex regions by removing segments that start at the first base of the acrocentric chromosomes 13, 14, 15 and 21.

#### Subclonal copy number consensus

Ignaty Leshchiner, Daniel Rosebrock

To produce subclonal consensus copy number, three callers that had the highest concordance in the subclonal segments were chosen: ABSOLUTE, Battenberg, and Sclust. For 3-star segments, consensus copy number was kept. For 1- and 2-star segments, if 2 out of 3 callers agreed on the allelic copy number state, then this copy number was used. If at least 2 callers were in agreement of subclonality of one allele’s copy number state, then the segment was called as subclonal. If ABSOLUTE produced one of the subclonal calls, then the consensus copy number value reported by ABSOLUTE was used (due to its internal Dirichlet Process clustering of states) if downstream merging of copy number segments was required (e.g., for calling arm level events). Segments for which all callers disagreed on copy number state were called as NA.

#### Validation of purity estimates

##### Comparison of purity against TCGA

Kerstin Haase

As a means to validate our consensus inferred purity values, we compared them to the published purities from Alizadeh et al. (2015). The authors provide consensus purities for 9,364 samples across 21 cancer types from the TCGA cohort. They analyzed the samples with four orthogonal methods and have integrated them into a consensus. The different utilized approaches include expression profiles of a panel of immune and stromal genes (ESTIMATE), somatic copy number data (ABSOLUTE), leukocyte unmethylation (LUMP) and image analysis by hematoxylin and eosin staining (H&E staining). Consensus purity is determined by normalizing all methods to give them equal means and standard deviations (CPE).

Even though the methods applied by Aran et al. (2015) are quite different to our consensus approach using WGS, our consensus has a high correlation with the one reported by them (R = 0.76) (Figure S1).

#### Subclonal architecture Methods

##### BayClone-C

Subhajit Sengupta, Juhee Lee, Yuan Ji

BayClone-C is an ultra-fast method to estimate the cancer cell fraction (CCF) for each single nucleotide variant (SNV) and group SNVs into clonal or subclonal clusters with different CCF values (Sengupta et al., 2015).

Using the consensus tumor purity (ρ), the consensus copy number calls and total and variant read counts for each single nucleotide variant (SNV), the algorithm estimates multiplicity and cancer cell fraction (CCF) for each SNV. Variant allele frequency (VAF) of SNV *s* is given by the ratio *n*_*s*_*/N*_*s*_ where *N*_*s*_ is the total number of reads and *n*_*s*_ is the number of reads with variant sequence. We assume

$${\text{VAF}}_{s}=\frac{CN_{s}^{M}\text{∗}\rho }{\rho \text{∗}CN_{s}^{T}+\left(1-\rho \right)\text{∗}CN_{s}^{N}}$$

where $CN_{s}^{T}\;$and $CN_{s}^{N}\;$are the total copy number at the SNV *s* locus in tumor cells and normal cells, respectively.$CN_{s}^{M}\;$is the mutation copy number of SNV *s*, i.e., the average number of copies of the mutant allele across subclones. We consider the case where SNV *s* lies inside a clonal copy number segment. Denote the allele-specific copy numbers at the SNV *s* locus by *q*_*1 s*_ and *q*_*2 s*_ (with *q*_*2 s*_ ≥ *q*_*1 s*_). Therefore, the possible integer multiplicity values are {1, …, *q*_*2 s*_}. Multiplicity for an SNV is estimated as the minimizer $m_{s}=argmin_{t\in \left\{1,\;\cdots ,q_{2s}\right\}\;}\left|1-\;\frac{CN_{s}^{M}}{t}\right|$. We then estimate CCF for SNV *s* as $\hat{CCF}=\frac{CN_{s}^{M}}{m_{s}}$.

We fit a finite mixture of Gaussian distributions to the estimated CCFs. We use the expectation-maximization (EM) algorithm (Dempster et al., 1977) to obtain the maximum likelihood estimate (MLE) of the mixture distribution. We run EM with different numbers of mixture components starting from one to seven and select the optimal number of components based on the Bayesian information criterion (BIC) (Schwarz, 1978). The R package *mclust* (Fraley et al., 2016; Scrucca et al., 2016) is used for implementation.

A postprocessing step of merging the initial CCF clusters is conducted where each CCF cluster corresponds to a Gaussian distribution in the previous step. The merger is needed because it is possible that Gaussian mixture components are not sufficiently separated from each other to be interpreted as different CCF values (Hennig, 2010), especially when a set of CCF values can be described by unimodal distributions that are not Gaussian. We use one of the modality-based merging methods known as the ridgeline unimodal method (Ray and Lindsay, 2005). This method aims to find a partition of mixture components such that it would create unimodal clusters but further merging would generate a non-unimodal cluster. See Figure 12 in Methods S1 for an example. After merging all the Gaussian mixture components, we obtain a set of CCF clusters. Then as a final step, we collapse all the CCF clusters with cluster means greater than 0.9 into a single clonal cluster with the new cluster mean equal to 1.0. SNVs in the final CCF cluster with mean equal to 1 or less than 1 are considered clonal or subclonal, respectively. Clustering membership gives the assignment of SNVs to each cluster. We merge the Gaussian mixture components using the *mergenormals* function in the R package *fpc* (Hennig, 2010).

##### Ccube

Ke Yuan, Geoff Macintyre, Florian Markowetz

The Ccube pipeline uses the consensus copy number, consensus purity and variant and reference allele read counts from consensus somatic point mutation calls.

The Ccube pipeline also produces an independent purity estimate using mutations from balanced copy number regions. For samples without whole-genome duplication, only mutations in normal copy number regions are included. For samples with whole-genome duplication, all mutations in balanced copy number regions are included. We then convert the allele frequencies to cellular prevalence estimates using the following equation:

$$f_{i}=\frac{\eta_{i}n_{i,tot_{t}}}{2(\left(1-\eta_{i}\right)n_{i,tot_{n}}+\eta_{i}n_{i,tot_{t}}}$$

Where $f_{i}$ is the VAF of the *i* th mutation obtained as the ratio between variant and wild-type allele counts, $\eta_{i}$ is the cellular prevalence of the *i* th mutation, $n_{i,tot_{n}},\;n_{i,tot_{t}}$ are the total copy number of normal and tumor populations respectively. We then cluster the $\eta_{i}$ using student’s-t mixture model. The model is fitted with the variational Bayes approach described in Archambeau and Verleysen (2007). The purity corresponds to the component with the largest mean. In addition, the eligible component must have at least more than 1.5% of mutation assigned to it.

Ccube is a Bayesian mixture model for clustering cancer cell fractions. Each mutation is assumed to be a sample from a mixture of Binomial distributions, each of which is parameterized by the total number of reads covering the position and expected VAF. The expected VAF, denote as $E\left[f_{i}\right],$ is parameterized as the following:

$$E\left[f_{i}\right]=\frac{\rho (m_{i}\left(1-\epsilon \right)-n_{i,tot_{t}}\epsilon )}{\left(1-\rho \right)n_{i,tot_{n}}+\rho n_{i,tot_{t}}}CCF_{k}+\epsilon$$

Where $CCF_{k}\;i$s the CCF of the *k* th cluster, ρ is the purity of the sample, ϵ is the sequencing error. The multiplicity is determined during clustering by the following:

$$m_{i}=\underset{m_{i}\in 1,\ldots ,n_{i,maj}}{\text{argmax}}\sum_{k}E_{q\left(z_{i,k},CCF_{k}\right)}\left[\log\;p\left(b_{i}|d_{i},\;E\left[f_{i}\right],z_{i,k}=1\right)\right]$$

Where $n_{i,maj}$ is the copy number of the major allele at the *i* th mutation, $z_{i,k}$ is 1 if the *i* th mutation is assigned to the *k* th cluster and 0 otherwise. $b_{i}$ and $d_{i}$ denote the variant and total read counts, respectively. p(.|.) is a conditional distribution, $E_{q\left(.\right)}\left[.\right]$ is an expectation function with respect to q(.), andq(.) is an approximation to the posterior distribution of the arguments. This approximation is obtained by the variational Bayes approach. The number of cluster is handled by a truncated Dirichlet Process prior for cluster-wise parameter $CCF_{k}$. Full details on the Ccube model will be presented elsewhere.

The solution with the best lower bound to the marginal likelihood of the model is selected. The solution consists of 1) posterior distributions of $CCF_{1,\ldots ,K}$ in terms of means and variances; 2) posterior probabilities of each mutation to be assigned to all clusters and final assignments; 3) multiplicities and observed CCFs based multiplicities. Clusters with less than 1% of mutations assigned are removed. The mutations are re-assigned in a variational expectation step. Clusters with mean CCF less than 10% apart are merged by re-running the fitting algorithm with the merged cluster configuration. A typical graphical summary can be found in Figure 13 in Methods S1.

##### CliP (Clonal structure identification through Penalizing on pairwise difference)

Kaixian Yu, Hongtu Zhu, Wenyi Wang

CliP is flexible on inputs: it requires either total copy number or allele-specific copy number, a rough estimation of purity, and single nucleotide variant read counts. However, the ideal set of inputs are allele-specific copy number profile, purity estimates, and single nucleotide variant read counts (counts supporting the variant and reference alleles), as are available in this study. The multiplicity of each SNV *i* is estimated by,

$$m_{i}={\left[\frac{r_{i}}{n_{i}\rho }\left(\rho C_{i}^{V}+2\left(1-\rho \right)\right)\right]}_{0}$$

where $r_{i}$ is the variant allele read count, $n_{i}$ is total read count, ρ is purity, and $C_{i}^{V}\text{is}\;$the clonal total copy number. The operator ${\left[x\right]}_{0}$ is defined as: ${\left[x\right]}_{0}=max\left\{1,\;\left[x\right]\right\},$ where [x] represents the closest integer from x.

CliP assumes each variant read $r_{i}$ comes from a binomial distribution $Binom\left(n_{i},\;\theta_{i}\right)$, where $\theta_{i}$ can be expressed as,

$$\theta_{i}=\frac{\phi_{i}m_{i}}{2\left(1-\rho \right)+\rho C_{i}^{V}}$$

where $\phi_{i}$ is the cellular prevalence (CP), $C_{i}^{V}$the clonal total copy number, and $m_{i}\;$the multiplicity of the SNV. Here, $\phi_{i}$ is our main interest. To ensure ${\phi_{i}}^{\prime }s$ fall in [0,1], a logit transformation is necessary; therefore,

$$\phi_{i}=\frac{e^{\omega_{i}}}{1+e^{\omega_{i}}}$$

and,

$$\theta_{i}=\frac{e^{\omega_{i}}m_{i}}{\left(2\left(1-\rho \right)+\rho C_{i}^{V}\right)\left(1+e^{\omega_{i}}\right)}$$

Then we formulate the problem as minimizing a penalized profile likelihood:

$$argmin_{\omega_{i}}\{\frac{1}{2}\sum_{i=1}^{N}\frac{n_{i}{\left(\theta_{i}-{\hat{\theta }}_{i}\right)}^{2}}{\theta_{i}\left(1-\theta_{i}\right)}+\sum_{1\leq i\leq j\leq N}p_{\lambda }\left(\left|\omega_{i}-\omega_{j}\right|\right)\}$$

where $p_{\lambda }\left(x\right)$ is a penalizing function, one may use a LASSO (Tibshirani, 1996) type penalty (L1), MCP (Zhang, 2010), SCAD (Fan and Li, 2001), etc.

The minimization problem is solved by Alternating Direction Method of Multipliers (ADMM). Distinct ${\phi_{i}}^{\prime }s\;\left({\omega_{i}}^{\prime }s\right)\;$represent different clusters.

A series of $\lambda^{\prime }s$ are used to generate solutions, selecting an appropriate λ can be achieved by information criterions (such as BIC, AIC). We also propose a bootstrap log-likelihood ratio test to select the penalizing parameter λ, which is much more computationally intensive. When an appropriate λ is selected, we further conduct a filtering step in which a cluster with fewer than 1% SNVs is merged with the closest larger cluster.

The purity can be estimated if we further make the assumption that the clonal cancer cell frequency (CCF) is 1. Under such an assumption, the purity is set to be the largest $\phi_{i}$. If the estimated purity is different from the initial input purity, i.e., larger than 0.01 in actual implementation, a rerun of the whole pipeline is performed with this newly estimated purity until the difference between two consecutive estimates is less than 0.01.

##### cloneHD

Ignacio Vázquez-García, Ville Mustonen

Given a mixed sample of the genomes of clones that have accrued single-nucleotide variants (SNVs), cloneHD reconstructs their evolutionary history from bulk DNA sequencing. This problem consists of three parts: (i) identification of subclones, (ii) reconstruction of subclone-specific profiles, and (iii) inference of evolutionary relationships between subclones.

We used consensus copy number and consensus purity as priors for cloneHD SNV clustering. The priors that cloneHD can use are the mean total copy number and the available copy number per locus. The mean total copy number is the mean copy number for each SNV locus of the entire sample, including both tumor and normal parts. The available copy number prior depends on the major allele state of the locus and allows mutations to be in states from zero up to the major allele state. For example, if a mutation was present before a chromosome underwent copy number gains, it would be present in all gained copies, whereas if it happened after the gain it would be present in fewer copies.

The cloneHD algorithm described in Fischer et al. (2014) identifies subclones and infers a subclone-specific posterior probability of SNV genotypes using a discrete state space HMM. The algorithm uses a genotype-based description, where the SNV genotypes of each subclone are encoded as hidden variables. Each subclone has an identity across the whole genome without a tree constraint on the relationship of the subclones. Mapping between subclones and clusters implies that when the model dimensions are increased by one subclone, two possible subclonal clusters are added at once (Figure 14 in Methods S1). As a result, alternative subclonal architectures can be problematic to resolve based on single samples from a tumor. To alleviate this problem, we extended cloneHD to allow for an SNV prior (‘tree prior’) to be learned from the data. This optionally enforces a tree structure on the SNVs and addresses the relationships between subclones. The other priors used in SNV mode reflect copy number and are described in the Supplementary Information of the cloneHD paper, Equations 34-35 (Fischer et al., 2014). They are computed from copy number and B allele frequency data, as described above.

To build an informative SNV prior, we take into account the lineage relations between subclones. Therefore, we treat the relation between subclones as a rooted tree, where the normal tissue is taken as the root, from which the clonal mutations of the sample are derived. Each node in the tree represents a cluster of mutations. On the way from this root to the terminal nodes, we assume that a cell may acquire a new mutation exactly once. Each of the connecting edges then represents mutations that distinguish the child from the parent node. In the subtree below this state switch, the genotype always remains mutated. This is commonly referred to as the ‘infinite sites’ assumption, because it is unlikely for the same mutation to occur twice.

We can describe the tree by the SNV genotype prior. The constraints on the tree phylogeny restrict the SNV genotype prior and thus the family of lineage trees that are possible. Here we implemented a set of tree priors shown in Figure 14 in Methods S1. Resolving the complete space of tree topologies is currently limited by the resolution of the data, but it may be possible to implement a generative model of tree structures, instead of constraints on the prior, which would require an integration over all possible trees.

Finally, we carried out model selection to determine the number of subclones that best explain the data. We required an improvement of 50 units of log-likelihood (for details of the scoring see the Supplementary Information in Fischer et al. (2014). We then converted from a representation of subclone genotypes to mutation clusters. To remove clusters that are not supported by sufficient high-confidence mutations, we required that the total sum of posterior probability of mutations to belong to a given cluster at 0.9 or higher probability was required to be > 10. This corresponds to a minimum of ∼10 high confidence mutations assigned to each cluster.

##### CTPsingle

Salem Malikic, Nilgun Donmez, Cenk Sahinalp

As input, CTPsingle (Donmez et al., 2017) takes reference and variant read counts for single nucleotide variant (SNV) calls. CTPsingle expects only somatic SNVs in its input; all germline SNPs should be discarded in advance. It also expects the mutations to reside in copy number neutral regions of the genome. In other words, CTPsingle assumes that all mutations in the input are heterozygous SNVs belonging to the regions that have not been affected by copy number aberrations. Mutations on non-autosomal (i.e., X and Y) chromosomes can be included if the gender of the patient is given. Tri-allelic variants are also discarded from the input. For each SNV, the numbers of reads supporting variant and reference allele are used to obtain a clustering of mutations as described in the next section.

For the given set of SNVs, CTPsingle uses a beta-binomial model to perform clustering of mutations in read count space Denoting the number of variant reads and the total number of reads covering an arbitrary mutation i as $y_{i}$ and $n_{i}$, respectively, we assume that $y_{i}$ is binomially distributed with unknown probability of success $p_{i}:$

$$y_{i}|\left(n_{i},p_{i}\right)∼Binom\left(n_{i},p_{i}\right).$$

The probability parameter $p_{i}$ is assumed to be generated from a Dirichlet process as given below:

$$p_{i}|G∼G$$

$$G|\left(\alpha ,G_{0}\right)∼Dir\left(\alpha ,G_{0}\right)$$

The baseline distribution $G_{0}$ is taken to be a Beta-binomial distribution with parameters $a_{1}$ and $b_{1}$. Since the Beta prior is conjugate to the Binomial distribution, resulting in a Beta-binomial posterior, inference can be performed using a standard Markov Chain Monte Carlo (MCMC) method as described in MacEachern (1998). Based on our earlier observations on simulated and real data, parameters α, $a_{1}$ and $b_{1}$ were set to 0.001, 5.0, 5.0, respectively. In order to mitigate overclustering due to a fraction of noisy mutation calls, we weakly constrained the minimum cluster size to at least 2% of the total number of mutations.

From the clustering step, we obtain the number of subclones (i.e., clusters) k and the assignment of mutations to subclones. After the clustering step, the mean allelic frequency $s_{j}$ for each subclone j=1,2,…,k is calculated as the average of allelic frequencies $y_{v}/n_{v}$ of all mutations v belonging to the cluster j. As the clustering is performed using variant read counts of heterozygous SNVs, cellular prevalence $s_{j}^{*}$ of subclone j is obtained as $s_{j}^{*}=2s_{j}.$ Given the cellular prevalence of each subclone, the purity of the sample is inferred as $p=\text{max}\left(s_{j}^{*}\right)$ where the maximum is taken over all indices j∈{1,2,…,k}. The output of the clustering step is illustrated in Figure 15 in Methods S1 where mutations are merged together and their counts are shown based on the values of $2y_{v}/n_{v}$ that reflect their cellular prevalence.

##### DPClust

Stefan Dentro, David Wedge, Kevin Dawson, Peter Campbell, Peter Van Loo

The DPClust pipeline takes as input an allele-specific (subclonal) copy number profile, a purity estimate and single nucleotide variants (SNVs) reported as chromosome, position and allele counts. The variant allele frequency (VAF) of each SNV is calculated through the reported allele counts using only the reads reporting the variant and wild-type alleles. With the VAF established, we obtain an estimate of the cancer cell fraction (CCF) of each SNV in three steps: First we approximate the copy number state of the SNV, its multiplicity, through:

$$u_{i}=\frac{f_{i}}{\rho }\left[\rho n_{tot_{t}}+\left(1-\rho \right)n_{tot_{n}}\right]$$

Where $f_{i}$ is the VAF of SNV i, ρ represents the tumor purity and $n_{tot\_t}$ and $n_{tot\_n}$ is the total copy number at the locus of SNV i in tumor and normal cells respectively. The multiplicity is established by rounding the approximate mutation copy number state $u_{i}$:

$$m_{i}=\left\{ \begin{array}{l} 1,\;u_{i}<1\\ \left|u_{i}\right|,\;u_{i}\geq 1 \end{array}\right.$$

Finally, the CCF estimate of each SNV is obtained through

$$CCF_{i}=\frac{u_{i}}{m_{i}}$$

DPClust is based on a Dirichlet Process (DP) that models the SNVs as drawn from a statistical distribution that consists of an unknown number of distributions. A posterior estimate of the number of internal distributions is obtained through Markov Chain Monte Carlo (MCMC) using stick-breaking. To account for read sampling variation on the number of observed reads, each variant is modeled as a draw from a binomial distribution. A description of the algorithm’s characteristics is provided in Bolli et al. (2014).

After completing the MCMC iterations, we aim to obtain three estimates: (1) An estimate of the finite number of distributions (cell populations), *K*, that are present in the input data, (2) the proportion of tumor cells that each population consists of $\left(CCF_{k}\right)$ and (3) likelihoods of each SNV belonging to each population. The number of cell populations *K* is determined by finding peaks in the posterior weight density (Figure 16 in Methods S1). In each iteration *j*, the stick-breaking procedure assigns a weight $\omega_{k\_j}$ to each cluster that represents its size and the cluster has a $CCF_{k\_j}$. Over many iterations, weight accumulates in the CCF space, where a large amount of weight corresponds to a high likelihood of the existence of a mutation cluster. We then obtain an estimate of *K* by obtaining all local maxima in the weight density.

With the *K* clusters and their locations $\left(CCF_{k}\right)$ established, SNVs can be assigned to clusters. We first establish the CCF area covered by each k∈K by finding the CCF location between each pair of neighboring clusters that corresponds to the minimum density. The minimum density on either side of a cluster represents its upper and lower CCF bound. Probabilities of a mutation belonging to a cluster are then established by accounting how often a SNV would have been assigned to each *k* throughout the MCMC iterations. Finally, small clusters are removed: Clusters smaller than 1% of total mutations in tumors with fewer than 150 SNVs and clusters with fewer than 30 SNVs otherwise.

##### PhylogicNDT

Ignaty Leshchiner, Dimitri Livitz, Daniel Rosebrock, Gad Getz

ABSOLUTE computes a probability distribution for the cancer cell fraction (CCF) of every mutation (independently). These distributions factor in sample purity and local absolute copy number.

PhylogicNDT utilizes called somatic variants and absolute copy number to perform Dirichlet Process clustering on the calculated CCF of the somatic variants in order to learn the underlying number of clusters from the data (Leshchiner et al., 2019).

In order to learn the underlying subclonal structure, we applied the Dirichlet Process as described by Escobar and West (1995) to the discretized CCF distributions of the variants. The mixing parameter was resampled iteratively from a prior gamma distribution as follows:

1) Sample eta: η=B(α+1,n) where *n* is the number of variants and *B* is the beta function.
2) Sample alpha:

$$\alpha =\pi_{\eta }G\left(a+k,b-log\left(\eta \right)\right)+\left(1-\pi_{\eta }\right)G\left(a+k-1,b-log\left(\eta \right)\right)$$

[Equation 13, Escobar and West, 1995)] where η is given above, *k* is the number of clusters, *a* and *b* are given by the prior, G(a,b) is a sample from the Gamma Distribution with shape and scale a and 1/b respectively, and $\pi_{\eta }$ is given by:

$$\frac{\pi_{\eta }}{1-\pi_{\eta }}=\frac{a+k-1}{n\left(b-log\left(\eta \right)\right)}$$

In each iteration, the probability of opening a new cluster is proportional to:

$$\frac{\alpha }{n-1+\alpha }$$

Within each iteration a Gibbs sampler over the mutations is run, where the probability of a mutation joining a particular cluster is proportional to:

$$\frac{m}{n-1+\alpha }$$

where *m* is the size of each cluster and is dictated by the likelihood of a particular mutation CCF distribution to be joined with other mutations present in the cluster at a given iteration.

Once the Dirichlet Process was completed for 10,000 iterations, we integrated the mutation probability distributions across all iterations and variants into a posterior density.

The prior mutational CCF distributions and the posterior cluster densities can be visualized with the distribution of clustered mutations across chromosomes (expected to be uniform) for quality control.

See Figure 17 in Methods S1 for an illustration of PhylogicNDT clustering.

The number of clusters, *k*, was then computed from this distribution by identifying all local maxima corresponding to peaks with sufficient density (1%). Mutations were then assigned to the best fitting detected cluster based on the similarity of their MCMC trace to the peak.

##### PhyloWGS

Jeff Wintersinger, Amit Deshwar, Shankar Vembu, Quaid D. Morris

We subsampled simple somatic mutations (SSMs, also referred to as SNVs) to a maximum of 5,000 per sample, selecting SSMs according to the provided priority list used across methods. We selected only SSMs located in genomic regions where the consensus copy-number state was known, allowing the method to correct for copy-number changes’ effects on observed variant allele frequencies.

PhyloWGS places copy-number aberration (CNA) events as mutations in its inferred phylogenetic tree based on their reported cellular prevalence. Although PhyloWGS can utilize subclonal CNAs, only clonal consensus CNAs were available for these analyzes, and so only those were provided to PhyloWGS. We modified PhyloWGS so that all CNAs were assigned to the same cluster, but did not require them to be assigned to the clonal cluster, thus permitting clonal clusters defined by only SSMs.

To input a CNA into PhyloWGS, we must know not only the cellular prevalence *p* of the CNA, set here to the consensus purity, but also the uncertainty in the cellular prevalence estimate (i.e., the standard error *e*). In the original PhyloWGS, we communicated these values to our Bayesian model by generating a “pseudo-SSM” for each CNA, and setting its variant reads *v* and total reads *d* to the values PhyloWGS would expect for an SSM with the same cellular prevalence and standard error.

Given the read depth *d* for the pseudo-SSM, we set *v* to represent the reported cellular prevalence by setting *v =* 0.5*^∗^pd*, meaning that *v* represents the expected number of variant reads for an SSM in a diploid region with a cellular prevalence of *p*. In general, we recommend setting the read depth *d* so that the standard error of the pseudo-SSM’s implied cellular prevalence would be equal to the standard error in the estimate of the CNA’s cellular prevalence. As PhyloWGS uses a binomial likelihood, we can approximate the standard error of the pseudo-SSM’s implied cellular prevalence as

$$e=\sqrt{\frac{p\left(1-p\right)}{\left(d+1\right)}}$$

and then solve for *d*. Note that *e* scales as the inverse of *d*, meaning that higher read depths produce smaller errors.

As standard errors for the CNAs were not available, we estimated them using a rule of thumb that let us compute *d* directly. Because most CNA-calling methods use heterozygous SNPs to determine cellular prevalence, and there are on average seven heterozygous SNPs per 10 kb of genomic sequence, we assumed that the standard error in the estimate of the cellular prevalence for a 10 kb CNA would be approximately equal to the cellular prevalence estimate error for an SSM cluster containing seven heterozygous SNPs. As such a cluster’s *d* value would be the sum of read depths for all seven SNPs, we set the *d* value for the pseudo-SSM representing a CNA *i* to be *d*_*i*_ = *L*_*i*_^∗^ *R* ^∗^ *D*, such that *L*_*i*_ indicates the length (in kb) of the genomic region affected by the CNA, *R* indicates the heterozygous SNP rate used to infer the CNA (i.e., 7 heterozygous SNPs per 10 kb), and *D* indicates the average read depth, taken as the median number of total reads across all SSMs. Next, we merged clonal CNAs into single large CNA events by summing their *d*_*i*_ values, preventing them from being assigned independently of one another to different (subclonal) populations in the inferred tree, producing *d*_*S*_ *= Σd*_*i*_. Finally, we determined the weight *d*_*C*_ for the single clonal CNA event *C* as *d*_*C*_ = min([*d*_*S*_], *A* ^∗^ *D*), such that *A* indicates the average number of SSMs per tumor (set to *A* = 3000, as this was approximately the average number of clonal SSMs in the PCAWG dataset). *A* represents the maximum confidence level we would have in the cellular prevalence estimate of a CNA in terms of equivalent number of SSMs. Thus, we inform the model that the standard error in the cellular prevalence estimate for a CNA must be no smaller than the standard error associated with a cluster of 3000 SSMs all possessing the sample’s average read depth. That is, we constrained the confidence of cellular prevalence estimates from SSMs and from clonal CNAs to be approximately the same. Consequently, for an average read depth of 50 and a purity of 80%, which are typical values seen in PCAWG datasets, this minimum standard error is:

$$e_{min}=\sqrt{\frac{p\left(1-p\right)}{\left(d+1\right)}}=\sqrt{\frac{0.8\left(1-0.8\right)}{\left(3000\text{∗}50+1\right)}}\approx 0.001$$

The PhyloWGS algorithm is fully described in Deshwar et al. (2015). In brief, we build upon the tree-structured stick-breaking process (TSSB) prior, using Gibbs sampling to sample trees, population frequencies, and SSM assignments via MCMC. After discarding the initial 1000 burn-in trees, we recorded 2500 trees for each dataset. The population frequencies were determined via 500 iterations of Metropolis-Hastings for each Gibbs sampling iteration.

PhyloWGS generates clone trees, while the PCAWG consensus efforts required only mutation clusters. As such, we discarded all tree-structure information, retaining only mutation clusters and their assigned frequencies from each MCMC sample. Clusters bearing less than 1% of total mutations were removed, with their SSMs reassigned on a per-mutation basis to whichever cluster offered the highest binomial likelihood assignment. We discarded polyclonal trees (i.e., trees bearing multiple independent clonal cancerous populations), rerunning PhyloWGS with a different random seed if more than 80% of sampled trees for a given dataset were polyclonal. On the remaining monoclonal trees, we removed “superclonal” populations. Superclonal populations were deemed to occur when the clonal population had a single child population bearing at least three times as many SSMs as the purported clonal population, with the child possessing no more than one child population itself (but any number of descendant populations). After removing superclonal populations, we assigned their SSMs to their single descendant, then set the descendant’s cellular prevalence to the mean of its own cellular prevalence and that of the former clonal population, weighted by the number of SSMs originally in each.

To generate a single solution for each dataset, we first determined the mode number of cancerous populations *K* across all MCMC samples, restricting further steps to sampled trees that also had *K* populations. To match populations between trees, we sorted the *K* populations from each sampled tree in order of descending cellular prevalence. Next, to establish SSM assignments to populations, we counted the number of times a given SSM was assigned to each of the *K* possible populations, reporting the mode assignment as our answer. To determine the per-population number of mutations, rather than simply report the count of summarized SSM assignments for each population, we better summarized the posterior consensus concerning mutation count by calculating the mean number of SSMs per population across selected trees. As the per-population values could then take non-integer values, we rounded these values to the nearest integers, distributing rounding errors by adjusting mutation counts upward or downward by one in the population with the most mutations, and continuing to modify mutation counts in populations ordered by descending number of mutations until the rounding error was fully distributed. Thus, though the per-population mutation counts may not match the reported SSM assignments, the sum of counts will match the total number of mutations assigned. Finally, we determined summary cellular prevalences for each population simply by calculating the mean cellular prevalences of each population across selected trees.

##### PyClone

Ke Yuan, Geoff Macintyre, Florian Markowetz

The PyClone pipeline uses consensus copy number, consensus purity and variant and reference allele read counts from consensus somatic point mutation calls (SNV).

The description of PyClone is in Roth et al. (2014). The model assumes that each mutation is sampled from a mixture of Beta-Binomial model. The cluster-wise parameter is a draw from a Dirichlet Process prior. For copy number, we use the parental copy number prior. The prior assumes the composition cells in terms of normal, reference and variant populations. The reference and variant populations are cancer cells. We set the two to have the same copy number profiles. The multiplicities are chosen among *[1, n*_*i,min*_*, n*_*i,maj*_*]*. The multiplicities are marginalized out with a prior, enforcing equal probabilities. The model is fitted to data with MCMC.

After discarding burn-in samples, we use MPEAR implemented in the R package mcclust to compute consensus assignments from the assignment traces. CCF cluster centers are obtained as median of CCF traces from the mutations assigned to the same cluster. The results consisted of 1) CCF centers; 2) final assignments; 3) multiplicities and observed CCFs. Clusters with less than 1% of mutations assigned are removed. Clusters with their CCF centers less than 10% apart are merged.

##### Sclust

Martin Peifer, Yupeng Cun, Tsun-Po Yang

From copy number states, estimated purity, and variant allele frequency we derive the cancer cell fraction of each mutation similar to DPClust. This serves as input for the mutational clustering module of Sclust. The general idea of our mutational clustering approach is that the histogram of SNV cancer cell fractions can be decomposed into a distribution of clonal populations and a distribution of sampling error due to the finite coverage. The distribution of the sampling error is approximated by a Gaussian with zero mean and average standard distribution derived from the cancer cell fractions. Next, the distribution of the clonal populations is derived by a deconvolution of the histogram of cancer cell fractions with the distribution of sampling error. To solve this ill-posed inverse problem, we use smoothing splines with a fixed smoothing parameter. Peaks in the estimated distribution of clonal populations constitute the cluster location of subclonal populations. Finally, mutations are assigned to the most probable peak using a binomial model.

##### SVclone

Geoff Macintyre, Ke Yuan, Florian Markowetz

For a detailed explanation of the SVclone algorithm and pipeline please see Cmero et al. (2020). Here we provide further details regarding the application of SVclone to PCAWG samples.

Using the consensus SV v*cf.*, consensus SNV v*cf.*, final consensus copy number profile, consensus purity estimate, and indexed WGS miniBAM file for each sample, the following preprocessing steps were carried out using SVclone:

• annotate - each of the SV pairs was annotated as coming from a deletion, inversion, translocation, or duplication
• count - normal and variant read counts for each SV were extracted from the miniBAM file and adjusted based on SV event type
• filter - the following filters were applied:
• any SV or SNV that was not matched to a valid copy number segment was removed
• any SV that lacked split or spanning supporting reads was removed
• any SV less than 1000bp was removed
• samples with greater than 4000 SNVs had their SNVs randomly downsampled to 4000 SNVs for clustering

The co-clustering mode of SVclone was used to simultaneously cluster and determine the CCF of SNVs and SVs. MCMC was carried out for 25,000 iterations (12,500 burn-in) to approximate the posterior distributions over model parameters. During clustering SVclone’s cluster merging procedure was used to merge clusters with overlapping CCF 95% confidence intervals. This procedure was repeated 8 times for each sample and the optimal run was chosen based on SVclone’s BIC-like measure, svc_IC. alpha and beta values for the gamma distribution prior over the Dirichlet concentration were set to 0.1 and 0.5 respectively.

Cluster membership for the variants used during clustering, and cluster mean CCFs, were determined as per SVclone’s default approach. Variants not used in clustering were retroactively assigned to the most likely cluster using SVclone’s post_assign procedure. Low-count clusters were filtered out (< 10 variants or cluster contains < 1% of variants) and variants from these clusters were re-assigned to the second most likely cluster.

#### Subclonal architecture consensus approaches

Kaixian Yu, Maxime Tarabichi, Amit Deshwar, Stefan Dentro, Ignaty Leshchiner, David C. Wedge, Quaid D. Morris, Peter Van Loo, Wenyi Wang

The 11 subclonal reconstructions described above were applied to the 2,778 cancer genomes in the PCAWG dataset and we aimed to combine the output into a robust consensus subclonal architecture. During this procedure, we used the PCAWG consensus SNVs and indels [Synapse ID syn7118450] and SVs [syn7596712].

We applied the 11 individual callers, described in the previous subsection, to a subset of ultra-confident copy number segments from the copy number consensus. Segments were ordered by their confidence level and selected up until at least 75% of the genome was covered. This ensured that the most confident copy number segments were used and that those with lower confidence levels could not introduce spurious mutation clusters.

To find mutation clusters, the methods were run on the provided consensus SNVs in the selected copy number regions only. This yielded 11 subclonal architectures that are described by four essential outputs:

• Number of mutation clusters identified,
• Number of mutations in each cluster,
• Proportion of tumor cells that each cluster represents,
• Mutation assignments to each cluster - either probabilistic or hard assignments of all SNVs to the clusters.

A fifth optional output is the mutation multiplicities, i.e., the number of physical DNA copies bearing the mutation, which is not provided by all individual methods but is used by CICC, one of the consensus approaches, to recalculate the proportion of tumor cells represented by the new consensus clusters.

The output of the 11 callers was used as input to the consensus procedure. We developed three orthogonal approaches (described below under Consensus approaches) that utilize different representations of the 11 subclonal reconstructions: WeMe uses cluster sizes and locations only, CSR uses mutation co-assignment probabilities, while CICC uses mutation-to-cluster hard assignments.

Each of these three methods returns output for three of the key subclonal architecture descriptors: the number of mutation clusters, the number of mutations in each cluster, and their cellular prevalence. The results reported in this paper are from the WeMe consensus method, but as we show below, we could have chosen CSR or CICC, which lead to similar results.

We ran individual methods together with the consensus approaches on two simulated datasets and on the PCAWG data. We derived a metric system in which we could score the results and evaluate robustness. The metrics and simulations are described in the following sections.

Importantly, individual methods perform downsampling when there are more than a given number of SNVs *N* to be clustered, each method *m* using its own *N*: = *N*_*m*_ to consider downsampling to *N*_*m*_ SNVs. Because CICC and CSR rely on mutation assignment to produce a consensus, we asked individual methods to downsample from a pre-randomized list of SNVs, taking the *N*_*i,downsampled*_ first SNVs from that list as input, where *N*_*i,downsampled*_ is the *i*^*t*h^ method’s threshold for downsampling. This ensured a minimal overlap of *min*_*i*_*(N*_*i*_*)* across methods to produce the consensus from.

Finally, to assign all SNVs and also indels and SVs, we provided the consensus subclonal architecture description, the full consensus copy number profile and all consensus SNVs, indels and SVs for which allele frequencies were available to the MutationTimer algorithm, described in Gerstung et al. (2020). MutationTimer assumes each mutation cluster can be modeled by a beta-binomial and calculates probabilities for each mutation belonging to each cluster while also taking into account the size of the mutation clusters. This step yielded the final consensus subclonal architecture with the aforementioned key features, while also performing timing of mutations relative to gains to classify mutations as clonal early, clonal not specified, clonal late and subclonal.

##### Consensus approaches

Because there is no straight answer to which output should be used to construct the consensus subclonal architecture nor how a method can integrate all outputs, we designed three independent consensus approaches, taking different inputs in order to build a consensus:

**Sparse clustering for subclonal reconstruction (CSR or “Caesar”)**, which takes the co-clustering probability matrix output of each method, i.e., the soft cluster assignment and extract common signals through non-negative matrix factorization (NMF);

**Cluster-ID Consensus Clustering (CICC or “Chic”)**, which takes the hard cluster assignment of each SNV and performs a hierarchical clustering on the distance of the assignments to identify SNVs that most often cluster together across methods;

**Weighted Median (WM or “Weme”)**, which takes a weighted median of the locations and the proportion of SNVs assigned to the location to construct a consensus location profile, ignoring the assignment of individual SNVs.

Not only are the consensus methods based on different principles (Figure 18 in Methods S1) but they also vary in their pre- and post-processing steps (see following subsections).

##### Caesar (CSR – Sparse Clustering for Subclonal Reconstruction)

When there are several subclonal callers that make distinctive calls, we expect that the sum of co-clustering matrices (CCMs) contains a certain amount of incorrectly clustered pairs of SNVs, as well as differences in SNVs that define cluster boundaries. CSR aims to identify the valuable common information from all methods and discard the disagreeing information through decomposition, in order to construct a cluster topology that reflects the strong signals contained in the sum of CCMs across callers. Specifically, CSR decomposes the sum of co-clustering matrices (CCMs) across all individual methods into a sum of a low rank matrix (signal) and a sparse matrix (noise). The low rank matrix captures the clustering information, and the sparse matrix takes care of possible mis-clustering and method-specific errors.

Suppose $M_{h}$ is a CCM of size N×N for method h, where N is the number mutations. The elements of $M_{h}$, $m_{ij}^{\left(h\right)}\;$takes value 1 if the i- and j-th mutation was clustered together by method h, and 0 otherwise. The mean of CCMs from each method is defined as

$$M=\frac{1}{H}\sum_{h=1}^{H}M_{h}$$

where H is the total number of methods to generate a consensus from.

To account for potential outliers, a thresholding was imposed on the mean CCM:

$$M=\left(m_{ij}\right)=\left\{ \begin{array}{l} m_{ij},m_{ij}\geq T\\ 0\;,\;m_{ij}<T \end{array}\right.,$$

In the PCAWG project, there were 11 (H=11) participating methods, and the threshold was set as T=0.2.

Next, the mean CCM, M, is decomposed into a sum of two matrices:

M=DA+Σ

where $D\in R^{N\times K}$, $A\in R^{K\times N}$, and $\Sigma \in R^{N\times N}$. The product DA is the low rank matrix (rank up to K≪N), and Σ the noise matrix. Such a decomposition is also called dictionary learning, where D is the dictionary, and A the code. Our main interest is matrix *A*, presenting a new set of features for mutations, which are less contaminated than the original features provided by M. We use the Python package SPAMS (version 2.1) to estimate D and A. Once A is obtained, a k-means algorithm is applied to $A=\left(a_{1},\;a_{2},\;...\;a_{N}\right),a_{i}\in R^{K}$ to identify mutation clusters. CSR requires as input the number of clusters, K. In the PCAWG project, K was chosen to be the median of all methods (rounded up if only even number of methods had results on the given sample).

With the clustering results, we further estimate the cellular prevalence (CP) for each cluster. Suppose the CP of SNV i estimated by method h is$\;\phi_{i}^{\left(h\right)}$, then the consensus CP of SNV i is defined as $\Phi_{i}=median\left\{\phi_{i}^{\left(h\right)}\;\right\}$. Subsequently, the cluster-specific CP is computed as

$$\Phi_{C}=\frac{1}{\sum_{i=1}^{N}I\left(i\in C\right)}\sum_{i=1}^{N}\Phi_{i}I\left(i\in C\right)$$

where C denotes a mutation cluster, and I(⋅) is an indicator function, taking value of 1 if SNV i belongs to cluster C, and 0 otherwise.

To make the obtained cluster architecture more biologically meaningful, several post processing steps are applied: 1) all the clusters with CP > *the consensus purity* (i.e., super-clonal SNVs) are merged with the closest non-super-clonal cluster. 2) Clusters that are too close in CP are merged to form one new cluster. The minimum distance between two nearest clusters is calculated as the median of the shortest distances between two clusters called by individual methods. When most methods call only one cluster, the minimum distance is set as 0.05. 3) Clusters containing too few SNVs will be dropped. The thresholding of tiny clusters is also inferred from results of each method, by taking the median of the smallest cluster sizes. When most methods give only one cluster, the minimum size is set as 5% of total SNV or 50 when the total number of SNVs is fewer than 1,000.

In samples that are hyper-mutated, i.e., their number of SNVs is above 30,000, a subsampling strategy is conducted to address computer memory limitations when constructing the CCMs, to reduce to 25,000 SNVs. The subsampling strategy is based on the distribution of CP for each SNVs and their frequencies of being called by individual methods. Then the CCMs are only computed for the sampled SNVs. This sampling strategy is preferred over a random uniform sampling as we need to ensure the subsampled distribution of CPs to represent the full distribution, so that clusters of smaller sizes are less likely to be missed due to down sampling. Besides, the ordered sampling based on frequencies of being called repeatedly guarantee that the maximum information from each method is used.

##### Chic (CICC – Cluster-ID-based Consensus Clustering)

Let $a_{i}^{\left(h\right)},\;h\in \left\{1,\ldots ,H\right\},$ andi∈{1,…,V} denote the cluster ID/label of the i-th SNV assigned by method h, and H denote the total number of methods and V the total number of SNVs assigned by at least one method. If thei-th SNV was assigned by method h, $a_{i}^{\left(h\right)}$ takes value from $\left\{1,\ldots ,\;K_{h}\right\}$, where $K_{h}$ is the number of clusters identified by method h; if not assigned it takes value of −1.

For the i-th SNV, we write the vector of assignment

$$A_{i}=\left(a_{i}^{\left(1\right)},a_{i}^{\left(2\right)},\ldots ,a_{i}^{\left(H\right)}\right)$$

If for $i,j\in \left\{1,\ldots ,V\right\},\;A_{i}=A_{j},\;$SNV i and j were assigned to the same cluster by all methods. There will be a limited number of distinct ${A_{i}}^{\prime }$s, let us denote these unique assignment vectors as $U_{l},l\in \left\{1,\ldots ,L\right\}$, and denote $N_{l},\;l\in \left\{1,\ldots ,L\right\}$ as the numbers of SNVs having the unique assignment vectors, respectively.

For each pair of $U_{i}$ and $U_{j},\;i\neq j\in \left\{1,2,\ldots ,L\right\}$, we define a distance between them as:

$$d\left(U_{i},U_{j}\right)=\sum_{h=1}^{H}d\left(a_{i}^{\left(h\right)},a_{j}^{\left(h\right)}\right),$$

Where

$$d\left(a_{i}^{\left(h\right)},a_{j}^{\left(h\right)}\right)=\left\{ \begin{array}{l} 0,\;|a_{i}^{\left(h\right)}=a_{j}^{\left(h\right)}\;and\;a_{i}^{\left(h\right)}\neq -1,a_{j}^{\left(h\right)}\neq -1\\ 1,|a_{i}^{\left(h\right)}\neq a_{j}^{\left(h\right)}\;or\;a_{i}^{\left(h\right)}=a_{j}^{\left(h\right)}=-1 \end{array}\right.$$

An extended distance between $U_{i}$ and $U_{j}$ is defined as

$$d_{ij}=10\max_{j\in \left\{1,\ldots L\right\}}N_{j}d\left(U_{i},U_{j}\right)-\text{max}\left(N_{i},N_{j}\right)$$

$D=\left(d_{ij}\right)$ was used to quantify the assignment distance between $U_{i}$ and $U_{j}$, and a hierarchical clustering with Ward’s criterion was performed on D to obtain the dendrogram.

If $K_{max\;}:=median\left(K_{i}\right)<3$, where $K_{i}$ is the number of clusters for method h, we set $K_{opt}=K_{max}$, where *K*_*opt*_ is the optimal number of output clusters. Otherwise, the Proportion of Ambiguous Clusters (PAC) (Șenbabaoğlu et al., 2014) was used to obtain $K_{opt}$. First, H methods were sampled with replacement from the original methods. For each $k\in \left\{2,\ldots ,K_{max}\right\}\;$we repeated the procedure 100 times to derive a consensus co-clustering matrix $M_{k}$ and set $K_{opt}={\text{argmin}}_{k}\;PAC\left(M_{k}\right)$.

We then cut the original dendrogram to obtain $K_{opt}$ clusters of ${U_{i}}^{\prime }$s. SNVs were assigned to the same cluster if their corresponding assignment vector $U^{\prime }$s were clustered together.

The CCF of each consensus cluster was calculated as the median of the consensus CCFs of the mutations assigned to that cluster. The consensus CCF of a mutation is computed as

$$CCF_{i}=\frac{p_{i}}{\rho m_{i}}\left(\rho C_{i}^{\left(T\right)}+\left(1-\rho \right)C_{i}^{\left(N\right)}\right)$$

where $p_{i}$ is the variant allelic fraction for SNV i, ρ is the consensus purity, $C_{i}^{\left(T\right)}$ is the consensus number of clonal copies, $C_{i}^{\left(N\right)}$ is the number of copies in the normal tissues and it is fixed to the diploid state, i.e., $C_{i}^{\left(N\right)}=2$ in our study, and the multiplicity is the floor of median of the multiplicities given by individual methods: $m_{i}=median\left\{m_{i}^{\left(1\right)},\ldots ,m_{i}^{\left(H\right)}\right\}.$ The cellular prevalence (CP) of the cluster is $CP_{i}=\rho CCF_{i}$.

##### Weme (WM – Weighted Median)

The Weighted Median (WeMe) method takes as input (i) a set of different *clusterings* of 1-d data and (ii) the proportion of outlier clusterings (r%) and it outputs a consensus clustering of the 1-d data with K clusters where K is a typical number of clusters in the set of clusterings. This consensus clustering has the property that, among all clusterings with at most K clusters, it minimizes the earth mover distance (EMD) to the *median clustering* of the (100−r)% inlier clusterings. The median clustering of a set of clusterings is a clustering that minimizes the sum of the EMDs from it to all members of the set, however, in general, this clustering has many more clusters than K. Below we define the term, *clustering* of 1-d data, formally.

A *clustering* of 1-d data is a set of ordered pairs $\left\{\left(p_{k},f_{k}\right)\right\}$, $k=1,\ldots ,K_{h}$, where $p_{k}$ is the proportion of data objects assigned to cluster k, and $f_{k}$ is the location of cluster k in the 1-d scale. In this study, $f_{k}$ corresponds to the cellular prevalence of cluster (i.e., lineage) k and $p_{k}$ the proportion of the SNVs assigned to that cluster. $K_{h}$ is the number of clusters of method h, this number can vary between the different clusterings of the data objects (e.g., SNVs in this study).

Here, each clustering is derived from a subclonal architecture summary generated by an individual reconstruction algorithm. These summaries provide for each subclonal lineage (i.e., cluster): a cellular prevalence of the lineage k, $f_{k}$, and the fraction of mutations assigned to that lineage, $p_{k}$. Importantly, Weme does not include any per-mutation information.

To justify and explain the WeMe algorithm, we first note that the EMD between any two clusterings in 1-d is equal to the integral of the absolute difference between their corresponding cumulative distribution functions (CDF). Specifically, the EMD between clusterings i and j, i,j∈{1,2,…,H} whose CDFs are $F_{i}\left(x\right)$ and $F_{j}\left(x\right)$, respectively is

$$EMD\left(F_{i},F_{j}\right)=\int_{-\infty }^{\infty }\left|F_{i}\left(x\right)-F_{j}\left(x\right)\right|dx$$

Where the $F_{i}\left(x\right)$ of a clustering, $\left\{\left(p_{k},f_{k}\right)\right\}$, $k=1,\ldots ,K_{h}$, is:

$$F\left(x\right)=\underset{i\in \left\{k|f_{k}\leq x\right\}}{\sum }p_{i}$$

Note, as indicated above, that the CDF, F(x), corresponding to a clustering is a piecewise constant function of x, with the discontinuities occurring at the values of $f_{k}$ (Figure 19, top left, in Methods S1). At these discontinuities, the y-value of the CDF jumps by $p_{k}$. Because of this, it is easy to compute the EMD between two methods, namely i and j. We just need to compute the area of, at most, $k_{i}+k_{j}$ different rectangles, where $k_{i}$ and $k_{j}$ are the number of clusters in the i- and j-th clusterings, respectively. These rectangles are delineated by the union of the cluster locations of the two clusterings - these are the discontinuities in each individual CDF (Figure 19, top right, in Methods S1).

We defined an unconstrained consensus clustering for a set of clusterings {1,2,…,H} as a new clustering $H^{*}$, $\left\{\left(p_{k}^{*},f_{k}^{*}\right)\right\}$, that minimizes the sum of the EMDs to all other clusterings, i.e.

$${\text{argmin}}_{\left\{\left(p_{k}^{\text{∗}},f_{k}^{\text{∗}}\right)\right\}}\sum_{h=1}^{H}EMD\left(F_{h},F_{H^{\text{∗}}}\right)$$

It is straightforward to prove that clustering $H^{*}$ has clusters at each location in the union of the cluster locations from the set of clusterings, and these clusters have their proportions set so that

$$F_{H^{\text{∗}}}\left(x\right)=median\left(F_{h}\right),\text{ for }h=1,\ldots ,H$$

Figure 19, bottom left, in Methods S1 displays a visual proof of this for H=3. Because $H^{*}$ is defined using the median of the CDFs for the other clusterings, we use the term *median clustering* to refer to the unconstrained consensus clustering $H^{*}$. However, that the median clustering can have many more cluster locations than a typical clustering in the set. We would prefer a consensus clustering that also has a number of clusters that is typical for the set of clusterings. As such, we define the K*-constrained median* clustering as the clustering $H^{W}$, $\left\{\left(p_{k}^{W},f_{k}^{W}\right)\right\}$, with no more than K cluster locations that minimizes $EMD\left(H^{*},H^{W}\right)$. Note that, in general, $H^{W}$ need not have cluster locations corresponding to any of those in the median clustering $H^{*}$. We will describe how to select K later, and for now, assume K is known.

To find the K-constrained median clustering $H^{W}$, we first compute the median clustering, $H^{*}$. Then we perform a grid search over possible settings of a vector p of length K whose elements represent the proportions of objects assigned to each of the K clusters. On the CDF, $p_{k}$ is the jump at the k-th discontinuity. Given p, we then set the corresponding cluster locations, f, to minimize $EMD\left(H^{*},H^{W}\right)$. We can minimize this by taking the weighted median of the cluster locations of $F_{H^{*}}\left(x\right)$ within the y-range of the step (Figure 19, bottom right, in Methods S1). We then set the K-constrained median clustering to be the one that minimizes this minimal EMD over all settings of p.

To define a robust WeMe consensus, we remove outlier clusterings, and compute the K-constrained median clustering only on the remaining, *inlier* clusterings. To identify the outliers, we do the following:

We compute the median clustering of all of the methods. We remove the reconstructions with the highest r% of EMDs to the median clustering (rounded down).

Then WeMe recomputes the median clustering using only the remaining clusterings, and computes the K-median clustering based on the new median clustering, setting K to be the ceiling of the median of the remaining clusterings.

##### Three random methods

The subclonal architecture summary metrics described below can be used to assess the performance of a subclonal architecture caller, where low scores mean a method is performing well. Often methods will not obtain the perfect solution and therefore their scores will deviate from the perfect score. What is not clear however is what performance can be reasonably expected. We reasoned that a caller should be able to outperform a simple random method and that running a method that yields a random subclonal architecture on the same data as the caller would then provide an upper bound of what can be considered reasonable performance. To this end we have developed two simple methods that generate subclonal reconstructions with varying degrees of randomness and one method that returns only clonal tumors.

The stick breaking method starts with drawing a random number between 0 and 6 to determine the number of clusters. It then orders the mutations by their CCF and iteratively breaks a randomly sized chunk of the ordered mutations. Each of these chunks represents a mutation cluster and its location is obtained by taking the mean CCF of the mutations in the chunk. Mutations are automatically assigned as they belong to a particular chunk. The advantage of this approach is that it is more likely to place a cluster where there are large real clusters, but it does also tend to place multiple clusters within large real clusters.

The downside of the stick breaking approach is that it performs a single series of breaks and returns that as a solution. We wondered whether the method could be improved by selecting the best solution from a series of random models. The informed method runs the stick breaking implementation described above 100 times. Contrary to the above approach, the informed method records the size and locations of clusters, but does not record the assignments. It applies the MutationTimer approach (Gerstung et al., 2020), which models each mutation cluster as a beta-binomial and takes into account the size of the cluster. MutationTimer then calculates the proportion of mutations that are poorly explained (i.e., fall in the outermost 5% of the beta-binomial distributions). This proportion is calculated for all 100 runs, after which the run that yields the lowest value is selected as the returned subclonal architecture.

A last and third approach places a single cluster to explain the data. It can obtain the single cluster by taking the mean mutation CCF, or it can be forced to place a clonal cluster at a CCF of 1. All mutations are assigned to the single cluster.

##### Evaluation metrics

For all methods, we evaluated two aspects of the solutions: scoring against the ground truth and the comparison of similarities between pairs of methods. The scoring system is used to assess the performance of all individual and consensus methods in the simulated data, whereas the similarity measures are used to compare the relative performance across methods on the PCAWG data, where there is no ground truth.

##### Performance on simulated data

We started using conventional evaluation metrics for clustering that are based on assignments, e.g., rand index and mutual information. However, not only are these metrics not consistent and have their own flaws (Kleinberg, 2003; Vinh et al., 2010; Wagner and Wagner, 2007), but also, in our setting, they turned out to be impractical. Indeed, not all individual methods assigned the same subset of SNVs to construct the subclonal structure. Importantly, the ultimate goal of the consensus methods was to identify the subclonal structure, i.e., number of clusters, number of SNVs within them and cellular prevalence (CP) for each cluster, whereas the assignments of SNVs, Indels, and SVs were performed post hoc by a uniform assignment method.

Therefore, we designed three metrics specifically for this study to measure the performance of the clustering results. These metrics reflected the important biological aspects of the results:

1) **Relative clonal fraction change**, characterizing the proportion of clonal events, is defined as

$$CFC=1-\frac{\left|F_{O}-F_{T}\right|}{F_{T}}$$

where $F_{O}$ is the clonal fraction reported by the method, and $F_{T}$ the true clonal fraction.

2) **Relative Number of clusters difference**, representing the relative subclonal events frequency, has the form of

$$NCD=1-\left|e^{\frac{C_{O}-C_{T}}{C_{T}}}-1\right|$$

where $C_{O}$ is the number of clusters from each method, and is the true number of clusters. This metric favored slightly underestimation over overestimation, which was designed to accompany the fact that the PCAWG working group project was reporting a lower bound of number subclones.

3) **Root mean square error,** reflecting overall architecture of subclonal composition, is calculated as

$$RMSE=1-\frac{\sqrt{\frac{1}{N}\sum_{n=1}^{N}{\left(\phi_{n}^{O}-\phi_{n}^{T}\right)}^{2}}}{\rho }$$

where $\phi_{n}^{O}$ is the method reported CP of SNV n, $\phi_{n}^{T}\;$the true CP of SNV n, and ρ the purity of the sample.

All metrics are rescaled so that 0 is the worst score and 1 the best. To summarize the metrics into a final score, a normalized rank approach is used, where the sum of ranks of each metric of method h, $r_{i}^{\left(h\right)},$ in sample i is normalized as

$$R_{i}^{\left(h\right)}=\frac{r_{i}^{\left(h\right)}-\min_{h}\left\{r_{i}^{\left(h\right)}\right\}}{\max_{h}\left\{r_{i}^{\left(h\right)}\right\}-\min_{h}\left\{r_{i}^{\left(h\right)}\right\}}$$

so that the best performing method is ranked as 1, and the worst 0.

##### Similarity in real data

We compared pairs of methods to assess the similarities of the solutions within PCAWG and simulated data. We used normalized difference over average to show the relative consistency of these consensus methods on PCAWG data, and investigate the robustness of consensus methods compared to each individual method.

The normalized differences are defined as follows, where 1≤i<j≤H, and H is the number of all methods including consensus methods:

1) The fraction of clonal SNVs:

$$CF_{similarity}=1-\frac{2\left|F_{i}-F_{j}\right|}{F_{i}+F_{j}}$$

where $F_{i}$ denotes the clonal fraction of the i-th method.

2) The number of clusters:

$$NC_{similarity}=1-\frac{2\left|N_{i}-N_{j}\right|}{N_{i}+N_{j}}$$

where $N_{i}$ is the number of clusters determined by the i-th method.

3) The RMSE:

$$RMSE_{similarity}=1-\frac{\sqrt{\frac{1}{\text{N}}\sum_{n=1}^{N}{\left(\phi_{n}^{\left(i\right)}-\phi_{n}^{\left(j\right)}\right)}^{2}}}{\rho }$$

where $\phi_{n}^{\left(i\right)}$ is the i-th method reported CP of SNV n, and ρ is the purity of the sample.

To account for diversities in dynamic range among samples, a standardization step: M-min(M)) / (max(M) - min(M), where M is a metric, was conducted to ensure each metric is distributed within interval [0,1].

##### Results

The 11 subclonal structure identification methods relied on different assumptions and clustering mechanisms (Figure 20 in Methods S1).

These differences explain why each method leads to different results and tends to perform better on a different subset of samples (Figure 21 in Methods S1).

This motivated the design of consensus methods that might combine the strengths of individual methods and be more robust across various tumor subclonal topologies (e.g., Figure 21F in Methods S1).

To assess the performance of the methods, two independent simulation sets were constructed. The first set, PhylogicSim500, contained 500 samples, where the copy number profiles were sampled from the PCAWG samples and the other parameters were independently sampled from fixed distributions (Simulation of subclonally heterogeneous samples – PhylogicSim500). To allow individual methods to debug and tune their parameters, the true subclonal architectures were released along with the PhylogicSim data. The second set, SimClone1000, contained 965 samples and used a grid design to cover the collection of scenarios encountered in the PCAWG samples. The ground truth of SimClone1000 data was not released before the final evaluation of all methods; the design was also kept hidden and made the inference of the grid difficult.

As described in Evaluation metrics, to assess the performance of all the methods, we established an evaluation system using high-level summary metrics, corresponding to the biological variable of interest:

1) clonal fractions, which describes how well a method is able to estimate the fraction of (sub)clonal SNVs;
2) number of clusters, which is essential in clustering analysis and represented how well each method identifies the number of distinct subclones; and
3) root mean squared error (RMSE) of mutation assignments, which characterizes how well the overall subclonal architecture matches another architecture (see supplementary methods).

To gain an overall performance assessment, the total score for a given method in a given sample was obtained by averaging the ranks of the three metrics in the sample for this method.

Individual methods lead to variable results while consensus methods lead to more robust and accurate results in simulated data. The total scores (Figure 22 in Methods S1) show that the consensus methods were ranked at the top, together with the best individual methods. There are large variations in performance across individual methods within and across two simulation settings. However, we observed the two simulation sets yielded similar results with the consensus methods, which confirmed that the consensus methods achieved robust and consistent results as compared to individual methods.

In the detailed comparison of the three metrics themselves, each individual method topped a metric in a fraction of the samples but not all. Whereas some individual methods were globally significantly better than others on the simulated data, the scores of the consensus methods were not significantly lower than those of individual methods on both datasets - between the best consensus and the best individual method the Mann Whitney U tests fdr = 0.029 for PhylogicSim500, and fdr = 0.336 for SimClone1000.

Given the design of the SimClone1000 simulations, we could ask how parameters such as true number of subclones and the number of reads per tumor chromosomal copy (nrpcc) influenced the absolute and relative performances of the methods (Figure 23 in Methods S1). As expected we observed: an improvement of the performance with increasing power (nrpcc); that CliP systematically overestimated the number of subclones when there were none; and that most methods performed well at integrating copy number changes, except from CTPsingle, whose relative performance significantly improved in fully diploid tumors (not shown). Most importantly, the consensus methods performed competitively across the grid of nrpcc and number of subclones (Figure 23 in Methods S1), further suggesting that they combine the strengths of individual methods. This was also true when we looked at performance only in samples for which the scores for number of subclones were equal, demonstrating that the high performance of the consensus did not stem only from taking the median number of subclones across individual methods (not shown).

Top performing methods lead to more similar results in both simulations sets and real data, suggesting that their performance remains the same in real data. Top performers displayed higher similarity (Figure 24 in Methods S1). Whereas individual method’s performance varied across independent simulation sets, the consensus methods consistently showed higher similarities than individual methods. Interestingly, individual methods were more similar to the consensus methods than they were to each other.

As expected, the consensus methods were the most similar to each other. Few pairs of methods showed high similarity, although not as consistent across the two datasets as the three consensus methods. As desired, the random methods had a quite uniform distribution of similarities against any method.

Finally, running on more methods leads to better results. We assessed the performance of the consensus methods running on 2 to 11 input individual methods. We ran CSR, WM, and CICC on all 965 simulated tumors from SimClone1000 for all of the 2,035 combinations of input methods, i.e., 1,963,775 times. This showed that performance increased with number of methods taken as input, suggesting a consensus of all methods is guaranteed to provide results at a median-ranked performance, and safeguarded from low performance (Figure 25 in Methods S1).

Integrating more methods into the consensus improved the lower bound and the median of the median performances. Few combinations of a subset of the methods significantly outperformed the individual methods on this dataset. These particular combinations, however, would be dataset-specific and not generalizable to real PCAWG data.

In conclusion, in the absence of ground truth, the high similarities between consensus methods in real PCAWG and in simulated samples, together with their consistently high performance on simulated data, suggested robust accurate reconstructions through consensus clustering on PCAWG data. Each individual method showed high performance across a range of scenarios and added to stabilize the consensus results.

Therefore, we applied the consensus approaches to the outputs of the 11 subclonal structure reconstruction methods on 2,778 PCAWG tumor samples and settled on using the output of one of the consensus methods, WeMe – this choice of consensus approach was arbitrary, as all three led to similar results.

#### Power correction

Somatic mutation callers require several high-quality supporting reads to reliably call a mutation. In samples with low purity and/or low allele fraction subclones, the observed allele fraction and cancer cell fraction (CCF) of the clones will be biased upward, since the signal from allele count fluctuations that fall below the caller’s detection limit will be unaccounted for (variants are not detected). If subclonal reconstruction is done without correcting for of such biases, the lower CCF cluster positions would be inaccurate and subclonal cluster sizes (number of mutations) would be significantly underestimated.

We wish to correct for the effects of WC on both the inferred CCF of identified subclonal lineages and the number of SNVs belonging to that lineage. For this purpose, we have developed two orthogonal methods as described below.

##### PhylogicCorrectBias

Ignaty Leshchiner, Dimitri Livitz, Gad Getz

To measure and correct the bias caused by the WC effect, we employed a simulation-based search method, PhylogicCorrectBias **(**a component of the PhylogicNDT package), that empirically estimates the correct CCF cluster position and its mutational composition. In case there is no bias, the CCF of the simulated clusters will match that of the detected sample.

To perform the simulation, a potentially truncated CCF cluster of mutations is investigated as follows. Simulated mutations are randomly placed on an allele along the genome according to the copy number profile and bases at risk at a chromosome/allele, while their expected allele fraction is selected according to the current cluster CCF and local coverage. Subsequently, the algorithm defines the measured number of alternative and reference reads given the expected allele fraction according to a binomial distribution and decides if the mutation is “detected” based on the filtering and detection criteria of the callers used. The simulated cluster CCF is then calculated from the “detected” mutations. In the event the values of the simulated and experimentally measured clusters do not match, the algorithm iteratively lowers the real cluster CCF downward in 0.01 increments (maximum CCF resolution) until the “detected” cluster CCF and mutation number match the experimentally measured cluster position, within 0.01 CCF delta. On convergence of the algorithm the corrected cluster position and expected mutation count is reported.

##### SpoilSport

Amit Deshwar, Quaid D. Morris

To correct for the WC, we make some simplifying assumptions about how the cellular prevalence (CP) estimates for each lineage were derived. Specifically, we assume that the CPs were maximum likelihood estimates under a binomial sampling model where all variants are at average read depth and exist as a single copy in a region of copy number equal to the average ploidy of the tumor. To estimate the magnitude of the WC effect we first convert from CP to a probability of observing a variant read using the following equation:

$$p_{v}=\frac{\theta }{\rho \psi +\left(1-\rho \right)2}$$

Where θ is the CP of the lineage, ρ is the purity of the tumor and ψ is the tumor ploidy. The denominator of this equation is the average copy number in the sample.

Given $p_{v}$, we use moment-matching to find the truncated binomial distribution with mean observed success probability equal to $p_{v}$. The PDF of the truncated binomial distribution truncated at T successes is:

$$\text{TruncBinom}\left(x,p,n,T\right)=\left\{ \begin{array}{l} 0,\;x\leq T\\ \frac{\text{Binomial}\left(x,p,n\right)}{\sum_{j=0}^{T}\text{Binomial}\left(j,p,n\right)},\;x>T \end{array}\right.$$

There is no closed form equation for the mean but it can be calculated conditioned on *T, p* and *n* by summing over values of *x* from 0 to *n*:

$$E\left[\text{TruncBinom}\left(p,n,T\right)\right]=\sum_{x=0}^{n}x\text{ TruncBinom}\left(x,p,n,T\right)$$

We set *n* to equal the rounded mean read depth of variants in the lineage we are correcting and find *p* by solving the following optimization problem:

$$p^{\text{∗}}=\underset{p}{\text{argmin}}\left|E\left[\text{TruncBinom}\left(p,n,T\right)\right]-p_{v}\right|$$

We solve this problem using the bounded *localmin* procedure as implemented in the R function *optimize*.

After finding *p^∗^* we convert this back to CP by multiplying by the average copy number. This is the WC corrected CP used for downstream analysis.

We additionally infer the ratio of true variants to observed variants (sf) by inverting the untruncated proportion of the probability mass of the binomial distribution:

$$sf=T\frac{1}{1-\sum_{j=0}^{T}\text{Binomial}\left(j,p^{\text{∗}},n\right)}$$

##### Power correction consensus

Ignaty Leshchiner, Amit Deshwar, Dimitri Livitz, Quaid D. Morris, Gad Getz

Consensus correction for the effect of mutation detection power was produced by integrating the values reported by the two methods above. Consensus CCF shift correction was obtained as the mean between the reported values, while for the corrected number of mutations in the subclone a geometric average was used. In a small set of samples a most conservative solution was used when available.

#### Validation of subclonal reconstruction

##### Simulation of subclonally heterogeneous samples – PhylogicSim500

Ignaty Leshchiner, Daniel Rosebrock, Dimitri Livitz, Gad Getz

To generate simulated data, we followed specific guidelines to ensure biologically realistic and heterogeneous data. The software simulator, PhylogicSim, (another component of the PhylogicNDT package) was used to generate 500 samples with diverse heterogeneity profiles. First, we randomly generated cluster CCFs, as well as the number of mutations in each cluster. A clonal cluster was included in each simulated sample. The number of subclones (N) in each sample was chosen randomly from the set (0, 1, 2, 3, 4) for samples with linear evolution, and (2, 3, 4) for samples with branched evolution, where samples with branched evolution always contained two dominant sibling clones whose CCFs summed to 1. Cluster CCFs were furthermore chosen to have a distance of 0.1 from the nearest cluster CCF, and to be at least 0.1 in magnitude. The number of mutations in each sample was chosen from a uniform distribution with domain 2,000 - 7,000, U[2000,7000]. The proportion of mutations assigned to each cluster was then estimated by taking N random numbers from U[0,1], dividing by the sum, and assigning each of these proportions randomly to a cluster.

Samples were chosen by inspecting the PCAWG consensus copy number profiles across all cancer types, selecting tumors where at least 70% of the genome was covered by clonal 3-star segments (N = 853 samples). We then used the ABSOLUTE output as the copy number profile for the simulated tumor. Each segment with a subclonal allelic copy number state was rounded to the nearest clonal copy number state. Segments were then assigned randomly to subclones in the same CCF space as the mutation clusters. For each sample, purity was chosen from the distribution of consensus purities across all called PCAWG samples.

We used a beta binomial model to simulate coverage profiles (number of reads at each locus in the genome) for each simulated sample. To do this, all normal (non-tumor) coverage profiles from all PCAWG samples were fit to a beta-binomial distribution, optimizing parameters alpha and beta in the beta-binomial pdf, leaving n = 500 fixed. For each simulated sample, we chose a mean coverage by sampling from the distribution of mean coverages of all tumor samples in PCAWG (total mean coverage = 53.6x). We then chose alpha, beta, and n from the beta-binomial fits of all normal coverage profiles that had the closest mean to the sampled tumor coverage. Coverage at each site in the simulation was then chosen at random from a beta-binomial distribution using these alpha, beta, and *n* parameters, scaling accordingly to account for local copy number changes.

Using the actual (true) CCF of the mutation, multiplicity, and allelic copy number information at the site of the mutation, we calculate expected allelic fraction for each SNV site. Finally, the measured variant allele count for each SNV site is chosen from a binomial distribution ∼B(n,p), where n is the coverage at the site, and p is the expected allelic fraction of the mutation.

For each simulated SNV the following information is used to calculate the expected (true) allelic fraction:

• mult = multiplicity of mutation
• $CCF_{mut}$ = c*cf.* of mutation
• ρ = sample purity
• $CN_{cl}$ = local absolute copy number (alleles A+B) in clonal fraction (i.e., $1-CCF_{scna}$)
• $CN_{sub}$ = local absolute copy number (alleles A+B) in subclonal fraction
• $CCF_{scna}$ = c*cf.* for subclonal copy number event

To calculate the expected (true) allelic fraction we used the following measurements.

a) in case of no sub-clonal CN alterations:

$$af=CCF_{mut}mult\rho /2\left(1.0-\rho \right)+\rho CN_{cl}$$

b) in case of sub-clonal CN alterations:

$$mult=mult_{sub}CCF_{scna}+mult_{cl}\left(1-CCF_{scna}\right)$$

$$af=\frac{CCF_{mult}mult\rho }{2\left(1.0-\rho \right)}+CN_{sub}CCF_{scna}\rho +CN+CN_{cl}\rho \left(1-CCF_{scna}\right)$$

we allow a) $CCF_{mut}=1$or b) $CCF_{mut}=CCF_{scna}$ and $mult_{cl}=0$ or c) $CCF_{mut}=1-CCF_{scna}$ and $mult_{sub}=0$

We have used the above-simulated datasets for extensive quality control and validation of the individual reconstruction methods as well as the performance of the consensus subclonal reconstruction. In general, 11 subclonal reconstruction methods as well as the consensus approaches used in this manuscript produced consistent results (Figure 26 in Methods S1) on the simulated datasets. In some samples, specific methods deviated from the majority, justifying the consensus approaches.

As an additional metric “obtainable” truth was calculated from the true cluster positions, based only on mutations that mutation callers would discover (which results in a CCF shift of cluster positions and sizes) and by performing additional pairwise cluster permutation significance test to identify pairs of clusters which would be statically undistinguishable from a single cluster (p value > 0.05).

We have generated a set of simulated samples to specifically explore the resolution of single sample subclonal reconstruction. We have simulated sample sets with varying purities and cluster-to-cluster distance series ranging from 0.2 to 0.03 CCF inter-cluster distance. In summary, even in the purest samples, none of the methods were able to consistently resolve clusters that were less than 0.07 CCF apart. These results provide an extra argument for potential additional intra-tumor heterogeneity within subclonal reconstructions in sequenced samples, especially when subclonal CCFs overlap in single sample cases.

The simulated datasets were used to extensively benchmark and validate the power correction methods. When run on the “detected” portion of simulated variants they accurately recapitulated the true CCF of the subclonal clusters and properly corrected the associated number of mutations.

##### A grid approach to simulations of tumors – SimClone1000

Maxime Tarabichi, Stefan Dentro, Quaid Morris, Wenyi Wang, Moritz Gerstung, David Wedge, Peter Van Loo

In addition to the PhylogicSim500 simulations, we simulated an independent set of 700 tumors using the SimClone simulator, for which the truth was hidden. We opted for a grid approach to explore the performance of the methods across a range of values for four parameters: number of reads per tumor chromosomal copy (nrpcc), number of subclones, fraction of clonal SNVs, and number of clonal SNVs.

Additionally, we resimulated 300 of the 700 samples in a fully diploid setting with the exact simulation parameters, including nrpcc, allowing us to assess the impact of copy number aberrations on performance.

We simulated subclonal architectures by first establishing a series of required parameters (see the next section) and provide these to the SimClone simulator. In this instance, SimClone takes a subclonal architecture design (number of mutation clusters, the proportions of tumor cells that each cluster takes up and the number of mutations per cluster) and a copy number profile (genome wide copy number and a purity value).

SimClone makes a number of assumptions during its simulation runs: Both the mutation and wild-type alleles are supported by a number of reads. We assume that the distribution of the number of mutation-supporting-reads takes on the shape of a binomial distribution. To model variation on the total number of reads covering the locus where the mutation has occurred, we assume that the depth can be modeled through a Poisson distribution. The mutation is carried by a number of chromosome copies (multiplicity). The shape of this distribution is partly determined by the copy number profile, that bounds the possible multiplicity states, and by cancer type specific development, i.e., if gains occur late there will be many SNVs on multiple copies, while if gains occur early there will be few. Multiplicity is modeled through a Poisson, and for the SimClone1000 dataset its parameter was set to 1. Finally, as a simplification, subclonal mutations cannot be carried by more or less than 1 chromosome copy.

With cluster locations and sizes provided as input, SimClone now simulates the mutations per cluster. Further input is required in a copy number profile, a tumor purity value, coverage and a multiplicity λ parameter (which is set to 1 by default). Mutations are generated by calculating the expected number of reads reporting the mutation and wild-type alleles. But the multiplicity must first be determined before those can be calculated.

The multiplicity (*mm*) is drawn from a Poisson distribution with the provided λ parameter as input.

$$m_{m}∼\text{ Poisson}\left(\lambda \right)$$

The mutations are randomly assigned to a copy number segment in the provided profile. If the multiplicity is not possible given the major and minor allele of the selected segment we adjust it to the copy number of the major allele.

Then the number of reads per chromosome copy for the tumor (*ct*) and normal (*cn*) cells are calculated from the total coverage (*C*), tumor purity (ρ) and tumor ploidy (ψ*t*). The total copy number of the normal cells is assumed to be 2:

$$c_{t}=C\frac{\rho }{\rho \psi_{t}+2\left(1-\rho \right)}$$

$$c_{n}=C\frac{1-\rho }{\rho \psi_{t}+2\left(1-\rho \right)}$$

The expected number of mutant alleles *rm* is determined by the multiplicity of the mutation, the mutation’s fraction of tumor cells (*f*) and the number of reads per tumor chromosome copy (*ct*):

$$E\left(r_{m}\right)=m_{m}fc_{t}$$

The expected number of wild-type alleles *rw* consists of three components: (1) Reads from normal cells (can be zero when the sample is pure and does not contain normal cells), (2) reads from whole chromosome copies from tumor cells that are not carrying the mutation (can also be zero when the copy number is 1+0) and (3) if the mutation is subclonal, an additional number of reads from cells that are not part of the subclone that carries the mutation:

$$E\left(r_{w}\right)=2c_{n}+m_{w}c_{t}+m_{m}\left(1-f\right)c_{t}$$

The total number of observed reads is then drawn from a Poisson distribution:

$$r_{d}∼\text{Poisson}(E\left(r_{m}\right)+E\left(r_{w}\right))$$

And the final mutant and wild-type alleles are determined by a draw from a binomial distribution:

$$r_{m}∼\text{Binomial}(r_{d},\frac{E\left(r_{m}\right)}{E\left(r_{m}\right)+E\left(r_{w}\right)}$$

The values for the grid parameters were taken to cover the range of values seen in the 2,778 samples from PCAWG and beyond.

The coverage of the simulations was fixed to the average coverage in PCAWG, coverage=48.46621.

Six purity clusters were selected using *kmeans* on the PCAWG purity values (Figure 27 in Methods S1). Five clusters for the number of clonal mutations were defined by *kmeans*, one of which was fixed at $N_{SNV}=10^{5}$, to better cover hypermutators, i.e., an important corner case in real data. Similarly, five clusters for the fraction of clonal mutations were defined using *kmeans*, one of which was fixed at $f_{clonal}=0.995$, to cover the typical corner case of melanomas, i.e., high *N*_*SNV*_ and high *f*_*clonal*_. Finally, four clusters of number of subclones were selected, corresponding to $N_{subclones}=\left\{0,1,2,3^{+}\right\}$, where the number of subclones in the 3^+^ categories was drawn uniformly from {3,4,5,6,7}.

This led to N=6×5×5×4=600 combinations of grid parameter values.

To limit the ability for individual methods to infer the grid design and thus tune their output to better (over-)fit the grid, we added noise in the input simulations at multiple levels:

i. grid coverage

From 600 combinations of parameters for the grid, i.e., the *wholebatch*, we randomly selected 700 combinations with replacement in two passes: a first selection of 600 combinations with replacement from *wholebatch* yielded *batch1*; a second selection of 100 combinations with replacement from {subbatch|subbatch∈wholebatch,subbatch∉batch1} yielded *batch2*. The final batch was the union of *batch1* and *batch2*. This two-pass strategy was selected as to maximize the coverage of the grid values. Using this two-pass strategy, we randomly selected 700 combinations, covering 462 out of the 600 combinations, i.e., 77% of the grid.

ii. input values

Input values were randomly selected from the clusters of PCAWG values defined by the *kmeans* clustering for purity, fraction clonal and number of clonal mutations.

iii. purity input values

The selected purity values *purity*_*in*_ were used to generate the simulations. However, the purity values given to the individual methods as input were defined as

$$purity_{out}=purity_{in}+purity_{error}$$

where $purity_{error}∼U\left(-mad_{purity},mad_{purity}\right)$. The median absolute deviations (mad) on the purity values were taken from the consensus purity values in PCAWG. This explains why some of the purity values were slightly greater than 1. However, we verified that no purity values were negative.

The copy number profiles were the consensus copy number profiles taken from the PCAWG sample from which the purity value was used. We only selected samples for which the fraction of genome aberrated $f_{aberrated}>0.1$, as measured from the consensus copy number profiles.

To assess the impact of copy number on the reconstructions, a random set of 300 samples from the 700 were simulated with fully diploid genomes, leading to a total of 1,000 simulations. Their purity was adapted to maintain the same average nrpcc, i.e.

$$purity_{diploid}=\frac{2}{ploidy_{non-diploid}}purity_{non-diploid}$$

The CCFs of the subclones were drawn from a uniform distribution $CCF_{subclone}∼U\left(0.1,0.9\right)$. The sizes of the clusters were defined using a stick-breaking procedure. Starting with a number of subclonal mutations $N_{subclonal}=\frac{N_{clonal}-f_{clonal}N_{clonal}}{f_{clonal}}$, we iteratively selected a proportion $p_{N_{clonal}}∼U\left(0.1,0.9\right)$ of the remaining SNVs and randomly assigned the obtained numbers of SNVs to the subclones.

The simulator removes mutations for which $alt_{count}<min_{count}$, with *min*_*count*_ = 3, which is at the origin of a power detection effect on the number of mutations.

To approach the target $N_{SNV}$ output by the simulator after this filtering, we compute $N_{input}$, such that over 10 simulations the average $N_{output}\in \left[N_{SNV}-\frac{N_{SNV}}{20},N_{SNV}+\frac{N_{SNV}}{20}\right]$. We find *N*_*input*_ using a dichotomic search.

We further removed simulations for which the total number of SNVs $N_{tot}<\min_{i\in PCAWG}N_{i}$ and for which the generation lasted longer than two days on our cluster nodes - due to the high number of SNVs. This led to a final set of 965 simulations.

#### Results of reconstructing the subclonal architecture of tumors

##### Power analysis and sample filtering

Kerstin Haase, Peter Van Loo

When determining the number of subclones in each sample, we are limited by multiple factors influencing the sensitivity of our subclonal reconstruction methods. Given that the PCAWG mutation-calling pipeline requires at least three variant reads to be able to detect a mutation, ploidy and purity of a sample as well as sequencing coverage impact the probability of a mutation being identified.

To account for varying purity, ploidy and sequencing depth in the analyzed samples, we calculated the number of reads per tumor chromosomal copy (nrpcc) to uniformly quantify the power to detect subclonal mutation clusters:

$$\text{nrpcc }=\frac{\rho \;\cdot \text{ cov}}{\rho {\text{ψ}}_{\text{t}}+\left(1–\rho \right){\text{ψ}}_{\text{n}}}$$

where ρ is te determined purity of the sample and *ψ*_*t*_ and *ψ*_*n*_ denote the ploidy of the matching tumor and normal sample, respectively. As we assume all germline samples to be diploid, *ψ*_*n*_ is set to two by default. We verified that the nrpcc is a strong factor influencing the number of identified subclones in a sample, whereas the total number of mutations identified does not impact the reconstruction (Figure S2A).

Given that a mutation can be detected when it is supported by at least three variant reads, we are theoretically able, in a sample with nrpcc of at least 10, to detect mutations in 30% of tumor cells or more. This consideration is supported by the reconstructions (Figure S2B). As a result, we decided to limit the downstream analyses to samples with a minimum nrpcc of 10 to exclude tumors that would appear completely clonal simply because of a lack of power to detect subclonal mutations.

Also, samples have been limited to those with less than 2% tumor contamination in the matched normal sample and no activity of any of the identified artifact signatures (Alexandrov et al., 2020). Only representative samples (ICGC/TCGA Pan-Cancer Analysis of Whole Genomes Consortium, 2020) from multi-sample cases are shown.

##### Results by individual methods are consistent with consensus results

Kerstin Haase, David Wedge, Peter Van Loo

After the results of the 11 subclonal reconstruction methods were combined by the consensus approach (section 2.2), we wanted to confirm that the final subclonal architecture does indeed reflect the individual solutions. For this purpose, we compared the fraction of subclonal mutations per cancer type between the 11 input methods and the consensus reconstruction. Overall, the distributions were very similar, and the ranking of cancer types by median fraction of clonal mutations was mostly conserved (Figure 28 in Methods S1).

As complex events, such as chromothripsis and chromoplexy, can introduce a large number of structural variants in a sample, we repeated this analysis after removing all SVs asscociated with complex events, but we could not observe a systematic shift in subclonality.

##### Correlation between fraction of subclonal mutations and mutation burden

Kerstin Haase, Maxime Tarabichi, Peter Van Loo

As the Figure 3 seemed to show a negative correlation between overall mutation burden and the fraction of subclonal mutations over the whole cohort, we investigated the Pearson’s correlation within each cancer type. After correction for multiple testing using Benjamini-Hochberg (Benjamini and Hochberg, 1995) only glioblastoma, medulloblastoma, and colorectal adenocarcinoma show a significant correlation (FDR < 0.05; Figure S2C), demonstrating that there is no systematic technical bias causing the amount of subclonal mutations to be dependent on mutation burden.

#### Selection and driver genes

##### Selection and dN/dS

Maxime Tarabichi, Iñigo Martincorena, Peter Van Loo

dN/dS is the ratio between the rates of nonsynonymous and synonymous substitutions. This metric has been extensively used for the detection of selection acting on protein-coding genes since its conception in the 1980s and has a long history in the field of molecular evolution. More recently, dN/dS has also been used to study selection in cancer genomes. In this study, we use a Poisson implementation of dN/dS, initially developed in Greenman et al. (2006), and further developed in Martincorena et al. (2015).

This implementation is feasible in the context of cancer genomics owing to the very low density of mutations per genome (typically < 1x10^−5^ mutations/bp), and it allows the use of complex trinucleotide substitution models that avoid biases due to context-dependent mutational processes (Martincorena et al., 2017). Point substitutions are classified according to two criteria: (1) the 192 possible strand-specific trinucleotide changes *i* considering one base up- and downstream of the mutant base, and (2) their functional impact (as synonymous *s*, missense *m*, nonsense *n* and essential splice site *e* substitutions). For example, the number of C > T missense mutations in an A[C]G context observed in a cohort of samples can be modeled as:

$$n_{ACG>ATG,m}∼Poisson\left(\lambda =r_{ACG>ATG}L_{ACG>ATG,m}\omega_{m}\right)$$

Where, *r*_ACG > ATG_ is the rate of ACG > ATG mutations per ACG site, *L*_ACG > ATG,m_ is the number of ACG trinucleotides in the sequences analyzed whose change for an ATG would lead to a missense amino acid change, and ω_m_ is the dN/dS ratio for missense mutations. Given that there are 192 by 4 possible nucleotide changes based on this classification, there are 768 equations like the one above. Maximum-likelihood estimates for all parameters as well as confidence intervals, including for all 192 parameters and the 3 possible ω (i.e., dN/dS) parameters are estimated using Poisson regression.

Using this framework, dN/dS ratios can be calculated for different groups of mutations, such as clonal and subclonal mutations in known cancer genes, which yields insights about the density of driver mutations in each group of mutations. With the dndscv (Martincorena et al., 2015) R package version 0.0.0.9 dN/dS analysis was run on the clonal and subclonal mutations as described above using the 566 cancer gene census from COSMIC v84 in *Tier1* (i.e., high confidence cancer genes) that were included in the hg19 *refCDS* gene list from dndscv. The maximum number of coding mutation per sample was set to 500.

##### Subclonal driver genes and their unique sequence

As outlined in the previous subsection, we ran dN/dS separately on clonal and subclonal mutations. This identified driver genes with dN/dS > 1 and q-value < 0.05 for clonal and/or subclonal mutations.

Running dN/dS on known cancer genes across all samples, we see that many known cancer genes that have a high dN/dS clonally, also do subclonally. For example, *KRAS* (dN/dS_mle_ = 14.2, q = 4.0x10^−5^) was the driver with the highest subclonal dN/dS for missense mutations, among other genes, such as *TP53* (dN/dS_mle_ = 12.4, q = 1.7x10^−10^), *SMAD4* (dN/dS_mle_ = 4.8, q = 4.3x10^−2^), and *PIK3CA* (dN/dS_mle_ = 6.8, q = 5.2x10^−9^), among many others. None of these known cancer genes were specifically subclonal.

Instead of looking at cancer genes only, we next extended our analyses to all genes and saw a few genes appear with a high dN/dS in the subclonal mutations only.

However, we noticed that those genes had a large fraction of their coding region displaying high sequence similarity with other genomic regions, as annotated in an independent study looking at calling somatic substitutions in the non-unique genome (Tarabichi et al., 2020). Because sequence similarities lead to alignment ambiguities, reads mapping to those regions will be randomly aligned across them. This can lead to a technical dilution of the local VAF of mutations falling in one of those regions, which in turn can lead to artificial subclonal assignment.

To illustrate this, we show the q-value from the dN/dS for each gene (clonal versus subclonal) in a scatterplot in Figure 29 in Methods S1, where the point size is correlated with the fraction of the coding sequence that is ambiguously mapped.

Using all genes, we see that *TP53* and *SMAD4* are the only two genes that have a unique coding sequence and for which subclonal mutations show a significant excess of non-synonymous mutations.

##### Gene set analysis of subclonally mutated genes

Ignaty Leshchiner, Dimitri Livitz, Gad Getz

The top 30 subclonal driver genes were subjected to gene set analysis using *GO_molecular_function* gene sets from MSigDB (Subramanian et al., 2005). We derived p values from the hypergeometric distribution for (k-1, K, N - K, n), where k is the number of genes in the intersection of the query set with a set from MSigDB; K is the number of genes in the set from MSigDB; N is the total number of genes; n is the number of genes in the query set. We obtained the false discovery rates by adjusting the hypergeometric p values for multiple-hypothesis testing according to Benjamini-Hochberg. Gene sets found to be significantly enriched are shown in Table 1 in Methods S1.

#### Analysis of phaseable mutation pairs

Jonas Demeulemeester, Amit Deshwar, Stefan Dentro, Ignaty Leschchiner, Maxime Tarabichi, Peter Van Loo, Quaid D. Morris

Phased SNV pairs can provide direct evidence of whether two SNVs co-occur in the same cell, and thus are in collinear subclonal lineages, or whether they are in separate cells, i.e., are mutually exclusive. Specifically, if read pairs cover the loci of two nearby SNVs, each such read pair is a pairwise haplotype and the pattern of these haplotypes can be examined to establish the ancestral relationships between the subclonal lineages containing the SNVs. Assuming that the haplotypes are accurate and controlling for violations of the infinite sites assumption (see 5.1.1), if two SNVs are in*-cis* (they appear on the same haplotype), they must be collinear. Furthermore, if two SNVs are in*-cis*, and one of the haplotypes contains only one of the SNVs, then that SNV occurred prior to the other along the lineage. In contrast, if two SNVs are in*-trans*, and they appear in a haploid region (e.g., on chromosome X in a male patient, or in a clonal copy number 1+0 region), then the two SNVs are mutually exclusive and must have occurred on distinct branches of the phylogenetic tree.

We obtained phasing information for all SNV pairs that are within 700bp. Upon first inspection, we noted that a lot of putative pairs harboring both somatic SNVs were located in low-complexity regions of the genome or contained multiple mismatches in the read (a sign of alignment artifact). We therefore applied the following stringent filtering, retaining only pairs with mapping quality ≥ 20, mismatch bases quality ≥ 25, no hard or soft-clipped bases, that were properly paired, were not flagged as duplicates and did not have a failed vendor quality control flag. Furthermore, we removed read pairs with indels and those that had ≥ 2 mismatches in a single read or ≥ 3 in the whole pair (if both phased SNVs were spanned by different reads in the pair). To further reduce biases we removed all pairs in regions known to undergo somatic hypermutation (T cell receptor α,β,γ,δ and the immunoglobulin heavy and light chain loci), and those flagged up as being part of kataegis events (ICGC/TCGA Pan-Cancer Analysis of Whole Genomes Consortium, 2020). In addition, pairs in the pseudo-autosomal regions of X and Y in males are excluded, as well as all pairs on consensus copy number segments with confidence level below d (see 1.3 Consensus copy number approach). Lastly, pairs on copy number segments which show evidence for a shifted CCF distribution of the SNVs on that segment, compared to the SNVs on all other confidence level a–d segments are also excluded (one-sided Mann–Whitney *U* test, Benjamini-Hochberg corrected *q* ≤ 0.01).

Having obtained high quality phasing information, we identify collinear phylogenetic relationships by looking for SNV pairs that meet the following criteria:

• ≥ 2 read pairs supporting both mutant alleles (Mut-Mut)
• ≥ 2 read pairs supporting only one of the two mutant alleles (either Mut-WT or WT-Mut, but not both)
• the SNVs fall within a single segment with major allele copy number equal to 1
• the lower bound estimate on the number of infinite sites violations < 1 or the copy number of the minor allele is equal to 0 and Battenberg reports no evidence for subclonality (see 1.2.3 Battenberg).

Branching phylogenetic relationships are revealed by looking for SNV pairs that meet the following criteria:

• no read pairs supporting both mutant alleles (Mut-Mut)
• ≥ 2 read pairs each in support of the two tumor haplotypes (Mut-WT and WT-Mut)
• the SNVs fall within a single haploid segment (copy number 1+0) and Battenberg reports no evidence for subclonality (see 1.2.3 Battenberg).

A total of 149,452 SNV pairs samples pass the quality filters above, are in the appropriate copy number context and have at least two reads supporting each haplotype. Of these, 140,259 are fully phased (93.8%), 8,140 are in-*trans* in a diploid region (5.5%), 774 and 277 support collinear and branching evolution, respectively (0.52% and 0.19%), and only 2 pairs (0.0013%) have inconsistent phasing.

The identification of collinear or branching evolution from phased SNV pairs in powered samples may be considered largely independent of the consensus subclonal reconstruction. While they both require copy number calls, this is only to identify informative regions for phasing. One can therefore use phasing results to assess the performance of the consensus subclonal reconstruction. Indeed, tumors identified to contain higher numbers of consensus subclones are enriched for collinear and branching pairs ($\chi^{2}$-test *p-value* = 3.9x10^−15^)

The frequency of branching versus linear evolution can be compared directly by subsetting linear pairs to only those that fall in regions where branching calls can also be made (i.e clonal haploid regions, see above). Unlike branching pairs, linear pairs can additionally encode clone-subclone relations. We therefore subset all linear and branching pairs to those where both SNVs have been assigned to subclones in the consensus. Considering the initially greater number of linear pairs and the probabilistic cluster assignments, we still expect the number of subclone-subclone reporting linear pairs to be inflated. As a result, the odds of branching versus linear subclonal evolution estimated here reflect a lower bound on the underlying distribution of subclonal evolutionary pathways.

##### Infinite Sites Assumption

Querying phylogenies through phasing analysis of proximal SNVs requires the infinite sites assumption to hold. Specifically, phasing to mutations that have occurred in parallel on both copies in a diploid region may be misinterpreted as evidence for a linear phylogeny. We opted for a simulation approach to establish a lower bound estimate of the expected number of such violations in any given sample.

In a single simulation, the observed SNVs in a sample are permuted across the callable regions of a diploid genome, retaining their trinucleotide context. Overlapping trinucleotides are considered to be independent from one another and any position on an allele can be hit only once (i.e., no back or forward mutation). For each sample, 1000 simulations are run, and the average number of these parallel mutations (double hits) counted. While retaining the trinucleotide context of mutations, these simulations assume a uniform distribution of mutations across the callable genome. Results from this model are hence regarded as a lower bound on the number of expected violations, since any deviation from uniform will only increase the rate of infinite sites violations.

##### Analysis of TRACERx 100 phylogenetic trees

To compare the odds of branching versus linear evolution obtained from phased subclonal SNV pairs in PCAWG to what is expected, we analyzed the detailed phylogenetic trees obtained through multiregion sequencing on the TRACERx 100 non-small-cell lung cancer cohort (Jamal-Hanjani et al., 2017). From each tree in the cohort, two subclones were randomly sampled and their relation on the phylogenetic tree (sibling or parent-child) was assessed. This analysis was performed 1,000 times. Every iteration, the odds were computed as the number of times sibling subclones were sampled divided by the number of times parent-child subclones were sampled. The median and 95% confidence interval are reported.

#### Tracking signature activities across cancer timelines

Yulia Rubanova, Quaid D. Morris

Mutations accumulate in cancer cells due to external (e.g., smoking, exposure to UV light) or internal (e.g., copy errors, failure of DNA damage repair) causes. Each source can be characterized by a probability distribution over 96 mutation types, known as a mutational *signature* (Alexandrov et al., 2013). The rate at which new mutations are generated by a certain process can vary over time and is known as the *signature activity*. Note that multiple mutational sources can be active in the same tumor.

We used a probabilistic method called TrackSig to fit the evolutionary trajectories of signature activities in individual tumors. This method is described in detail in a separate manuscript (Rubanova et al., 2020). Here we briefly describe (i) the source of the mutation signatures, (ii) the major steps in trajectory reconstruction by TrackSig, (iii) how significance of signature activity change was computed, (iv) how the estimates of coincidence between signature activity change and clonal / subclonal boundaries (and subclonal / subclonal boundaries) were computed. This analysis is based on mutation signatures obtained from PCAWG [syn8366024].

Note that this approach is complementary to the one employed in our recent study (Gerstung et al., 2020), which averages out signature differences between subclones (Figure 30 in Methods S1).

##### Source of mutation signatures

We used the Beta2 release of the mutation signatures by PCAWG working group 7 (Alexandrov et al., 2020). For each sample, we only considered mutation signatures marked as active.

##### TrackSig fitting of evolutionary trajectories of signature activity

First, we order the mutations based on their approximate relative order of occurrence in the tumor. Generally, if the mutation occurred early in the tumor development, it will be present at high cellular prevalence (CP), whereas late mutations have lower CP. We exploit this observation to infer the pseudo-time of mutation occurrence. We take the variant allele frequency (VAF) of the mutations as computed from the read counts. To eliminate the effects of quantization noise we then re-sample the VAF value from the posterior distribution given the variant and reference read counts. VAF estimates are converted into “average mutation copy number/cell”-space by multiplying the CP by the copy number. This space yields a “pseudo-time” ordering of mutations, but how this relates to real time is unknown. For convenience, we divide the time-ordered mutations into equal bins of 100 mutations. The resulting bins, each corresponding to one time point, constitute a pseudo-timeline of tumor development, which we leverage to map the signature trajectories. Timelines are constructed separately for each sample.

This method does not consider branching tumor evolution. If two subclones have a similar cellular prevalence, mutations from those subclones are likely to fall into the same time points. Note that this issue may be tackled by estimating the trajectories separately for each subclone.

Next, we estimate the signature proportions. At each time point, the SNVs are classified into 96 types based on their trinucleotide context. Then we fit a mixture of multinomials, where each component multinomial describes one of the known active signature distributions over the 96 types. Derived mixture component coefficients correspond to the signature activities and sum to 1 for each time point. The activity value reflects the fraction of the mutations generated by the associated mutational process. For ease of interpretation, activities are given as percentage. By estimating the activities at each time point, a set of trajectories is obtained. Each trajectory represents the activity of the corresponding mutational process during the evolutionary history of the tumor sample.

Finally, we seek to find the time points at which signature activities change substantially, as these represent changes in the activity of the different mutational processes. If certain mutational processes have become more or less active in a subclone when compared to the clone (e.g., inactivation of double strand break repair in the subclone), its emergence may be accompanied by a drastic shift in the trajectories. Otherwise, we expect the trajectories to remain relatively constant.

To detect the signature change points, we use Pruned Exact Linear Time (PELT) algorithm (Killick et al., 2012). PELT is based on dynamic programming. The algorithm iterates over the time points and re-computes the multinomial mixtures for all the mutations at both sides from the potential change point, treating each side as a single bin. We estimate the likelihood of the data split according to the potential change point. A point that maximizes the likelihood is considered a new change point. The Bayesian Information Criterion (BIC) is used to determine the optimal number of change points: introducing any new change point has to be accompanied by a sufficient increase in likelihood.

Note that change points are inferred without using any information from the subclonal reconstruction, allowing the method to make independent conclusions about signature behavior and its relation to subclones.

##### Evaluating uncertainty

To evaluate the uncertainty in activities, we resample the set of mutations 30 times and re-compute activity estimates. From our observations, the standard error across these bootstrapped activities does not exceed 5% absolute activity (with mean standard error 2%). Therefore, we omit change points with changes less than 5%. To evaluate overall signature changes in the tumors, we compute the maximal change of each signature as the difference between the maximum and minimum activity across pseudo-time. We consider that a tumor has a significant overall signature change if this difference exceeds 10%.

##### Comparing change points to CCF clustering

We investigate the relation between the signatures changes and CCF clustering. First, we determine where the CCF clustering boundaries map on our pseudo-timeline by dividing it according to the proportion of mutations per time point that belong to each CCF cluster. Distances in time are computed between those boundaries and our change points. To determine whether a subclonal boundary is supported by an activity change-point, we plotted the relative enrichment of change-points at a given offset from subclonal boundaries (Figure 31 in Methods S1). Using this figure, we deem a boundary to be supported if it has a change-point within 3 time points (tp).

We compute the percentage of clonal/subclonal and subclonal/subclonal boundaries that are supported by signature change points. In total, 54.7% of clonal-subclonal and 57.3% of subclonal-subclonal boundaries in all tumors are supported by our change-points (red bars in Figure 6D). These estimates are upper bounds on the number of supported boundaries, because some of the boundaries would be supported even if the change-points were randomly assigned to a time point.

To determine a lower bound on these percentages, we sample random points from the timeline and re-compute the distances between the boundaries and these random points. On average, 34.52% of clonal-subclonal and 34.49% of subclonal-subclonal boundaries are supported across 1000 random samples (green bars in Figure 6D).

To estimate the significance of boundary support, we sample random points 1000 times for each tumor and show the distribution of proportions of supported boundaries in Figure 32 in Methods S1. The empirical p value is computed as the number of occurrences when then proportion of boundaries supported by random exceeds the then proportion of boundaries supported by real change points of signature activities.

#### Analysis of drivers in clinically targetable genes

Stefan Dentro, David Wedge

To assess the potential clinical relevance of subclonal architecture inference, we considered driver mutations (SNVs and indels) in genes and pathways for which drugs have been developed or are currently in development. This allows us to estimate frequency of cases in which the only currently actionable mutation is subclonal. A list of targetable drivers was provided by the PCAWG driver group (Rheinbay et al., 2020). In this analysis we excluded the relapse tumors and all metastases except for the melanomas. For multi-sample cases, we only considered the PCAWG provided preferred sample for each donor.

Our consensus subclonal architecture approach produces probabilistic cluster assignments for each mutation. The procedure also identifies a mutation cluster as clonal (CCF of 1), allowing us to establish the probability that a mutation is clonal or subclonal. The procedure for driver mutations or protein-altering mutations is as follows: for each sample, establish the probability that all such mutations are clonal, that they are subclonal, and that there is at least one pair where one is clonal and one is subclonal.

The probability (p) of observing all n such mutations as clonal is:

$$\prod_{i=1}^{n}p_{i,clonal}$$

The probability of (p) of observing all n mutations as subclonal is:

$$\prod_{i=1}^{n}p_{i,subclonal}$$

Then the probability of observing at least one pair of mutations where one is clonal and one is subclonal is:

$$1-\left(\prod_{i=1}^{n}p_{i,clonal}+\prod_{i=1}^{n}p_{i,subclonal}\right)$$

The three probabilities were summed to create the three classes shown in the figures: *Clonal*, *Subclonal* and *Both*.

#### SV analysis and fusion clonality detection

##### Clonality analysis of recurrent structural variants

Geoff Macintyre, Ruben Drews, Florian Markowetz

The PCAWG Structural Variation Working Group identified 52 genomic regions containing significantly recurrent breakpoints (SRBs) and significantly recurrently juxtaposed regions (SRJs) (Rheinbay et al., 2020). As SRJs were mostly observed in small numbers of patients across individual tumor types, we focused our analysis here on SRBs, which were observed in larger numbers of patients across multiple tumor types.

Regions of SRBs ranged in size from 451 kb up to 5,651 kb (mean: 739 kb; median: 501 kb). 20 of the regions were enriched for amplifications, 9 for deletions, 9 for fusion events, and 5 for neutral rearrangements. Another 9 regions were characterized by general genomic instability and were therefore labeled “fragile” (Rheinbay et al., 2020).

SVs in fragile sites were considered candidate passengers rather than candidate driver events as they most likely arose due to genomic instability. To test this, we compared the difference in means of the number of subclonal SVs observed in fragile and the non-fragile loci with a Wilcoxon rank sum test. With a p value of 0.024, we confirmed an enrichment of subclonal SRBs in fragile loci, which supports the model that SRBs in fragile sites are indeed likely passenger events (Figure 33 in Methods S1). Hence, fragile SRB loci were excluded from our analysis.

The remaining 43 SRBs, covering 31,843 kb of the human genome, were used to determine which SVs were recurrent, and thus considered candidate driver SVs, and which were not recurrent, therefore considered candidate passenger SVs. For simplicity, SRBs were labeled by the name of the likely target gene lying within the SRB. Note: this did not imply that this particular gene was mutated or altered in any way. A minimum overlap of at least 1bp with an SV was required. A SV having both breakpoints inside a SRB was counted as one hit. An SV with one breakpoint outside and one inside was also considered as one hit for this particular SRB and the breakpoint outside was not used.

From 2,658 samples originating from 37 cancer histotypes, we filtered samples using the following criteria: samples classified as greylisted or excluded (75 samples) were excluded from analysis. For patients with multiple specimens, the preferred sample was chosen as determined by the latest PCAWG metadata file (36 samples excluded). Samples with nrpcc (number of reads per tumor chromosomal copy) values of less than 10 were excluded as they showed a strong bias toward clonal SVs. Within each of the 36 tumor histotypes, samples having total SV counts more than two standard deviations from the mean were considered “mutator phenotypes” and excluded from further analysis (77 samples). Cancer types with fewer than 10 patients were also excluded (8). The final filtered set of SVs contained 129,219 SVs from 28 tumor histotypes originating from 1,517 patients.

We hypothesized that candidate driver SVs differed in their subclonal probability compared to passenger SVs. We used 1 - the probability of an SV being clonal as output by *mutationTimer* (Gerstung et al., 2020) for SVs preprocessed by *SVclone* (Cmero et al., 2020). As the distribution of clonal probabilities was assumed to be of non-parametric character and non-identical, we decided to infer the significance of the difference in subclonal probability by permutation testing. Since we discarded overly mutated samples, we assumed that the distribution of subclonal probabilities of SVs were independent of the samples.

When calculating medians, we weighted them linearly by a sample’s individual nrpcc value by using the *weightedMedian* function of the R package *matrixStats.* This function allowed for the interpolation of results when ties arose.

The alternative hypothesis ***H***_o_ was formed by taking the difference in weighted medians of the subclonal probabilities of candidate drivers and passengers. The permutation testing established a null distribution ***H***_***0***_ of weighted differences between candidate driver and passenger probabilities by randomly assigning classification labels 10,000 times and taking the difference of the resulting medians. A p value was generated by taking the proportion of the alternative hypothesis lying outside the generated null distribution. The resulting p values were corrected for multiple hypothesis testing by multiplicity correction as proposed by Benjamini and Hochberg (Benjamini and Hochberg, 1995).

When plotting Figure 6A, the weighted interquartile ranges were used with the same linear weighing method applied as explained above. The IQRs were used as to represent the probabilities more closely to their density functions than the 95% confidence intervals as they tend to be very large due to the inherent noise of SV VAFs determined using short-read sequencing data (Cmero et al., 2020).

We used a GSEA-like test to see if any of the 43 SRB loci were enriched for clonal or subclonal SVs. For each SV, we transformed the subclonal probability, scaling by a weight vector representing the “detection power” of the sample in which each SV was observed. The weight vector was defined as the number of reads per chromosome copy for the sample, divided by the total across the dataset. All SVs were then ranked by their scaled subclonal probability. Using the fgsea package in R (Sergushichev, 2016), we computed an enrichment score for hits across the ranking for each SRB locus. The significance of the observed enrichment score was computed using a GSEA-like permutation test. p values were corrected for multiple testing correction using the Benjamini and Hochberg method (Benjamini and Hochberg, 1995). Significantly enriched loci and the proportion of tumor types contributing to the enrichment appear in Figure S5.

##### Fusion clonality analysis

Ignaty Leshchiner, Daniel Rosebrock, Gad Getz

A list of known driver fusions was curated by subsetting to fusions which appeared in at least 3 cases across the Jackson Lab fusion dataset (https://www.tumorfusions.org/), or which were recorded as drivers in the COSMIC Fusion dataset (https://cancer.sanger.ac.uk/cosmic/fusion) with at least 5 previously identified patient samples. Non-driver fusions were chosen by subsetting to fusions not classified as in-frame with known fusion partners from above. SV breakpoints were genotyped with BreakPointer (Drier et al., 2013) and SVClone, where mutant allele count was estimated by aggregating both chimeric read and read pair support of the fused allele, while reference allele count was estimated by summing both breakpoint spanning reads and read pairs supporting the reference allele. The likelihood of a fusion originating from a clonal or a subclonal cell population was then estimated with correction for local copy number and purity, according to PhylogicNDT model described above.

### Quantification and statistical analysis

All statistical details of the analyses are defined in the corresponding method section.

## Consortia

The members of the PCAWG Evolution and Heterogeneity Working Group are Stefan C. Dentro, Ignaty Leshchiner, Moritz Gerstung, Clemency Jolly, Kerstin Haase, Maxime Tarabichi, Jeff Wintersinger, Amit G. Deshwar, Kaixian Yu, Santiago Gonzalez, Yulia Rubanova, Geoff Macintyre, Jonas Demeulemeester, David J. Adams, Pavana Anur, Rameen Beroukhim, Paul C. Boutros, David D. Bowtell, Peter J. Campbell, Shaolong Cao, Elizabeth L. Christie, Marek Cmero, Yupeng Cun, Kevin J. Dawson, Nilgun Donmez, Ruben M. Drews, Roland Eils, Yu Fan, Matthew Fittall, Dale W. Garsed, Gad Getz, Gavin Ha, Marcin Imielinski, Lara Jerman, Yuan Ji, Kortine Kleinheinz, Juhee Lee, Henry Lee-Six, Dimitri G. Livitz, Salem Malikic, Florian Markowetz, Inigo Martincorena, Thomas J. Mitchell, Ville Mustonen, Layla Oesper, Martin Peifer, Myron Peto, Benjamin J. Raphael, Daniel Rosebrock, S. Cenk Sahinalp, Adriana Salcedo, Matthias Schlesner, Steven E. Schumacher, Subhajit Sengupta, Ruian Shi, Seung Jun Shin, Lincoln D. Stein, Oliver Spiro, Ignacio Vázquez-García, Shankar Vembu, David A. Wheeler, Tsun-Po Yang, Xiaotong Yao, Ke Yuan, Hongtu Zhu, Wenyi Wang, Quaid D. Morris, Paul T. Spellman, David C. Wedge, and Peter Van Loo.

## Supplemental information

Methods S1. Supporting Figures and Tables, related to STAR Methods

Methods S2. Members of the TCGA/ICGC Pan-Cancer Analysis of Whole Genomes Network
