# Supplementary material for: Characterizing genetic intra-tumor heterogeneity across 2,658 human cancer genomes
Source: Cell. 2021 Apr 15;184(8):2239–2254.e39. doi: 10.1016/j.cell.2021.03.009 (PMC8054914; doi:10.1016/j.cell.2021.03.009)
Supplement: Methods S1. Supporting Figures and Tables, related to STAR Methods [file mmc1.pdf]

Cell, Volume *184*

Supplemental information

Characterizing genetic intra-tumor heterogeneity across 2,658 human cancer genomes

Stefan C. Dentro, Ignaty Leshchiner, Kerstin Haase, Maxime Tarabichi, Jeff Wintersinger, Amit G. Deshwar, Kaixian Yu, Yulia Rubanova, Geoff Macintyre, Jonas Demeulemeester, Ignacio Vázquez-García, Kortine Kleinheinz, Dimitri G. Livitz, Salem Malikic, Nilgun Donmez, Subhajit Sengupta, Pavana Anur, Clemency Jolly, Marek Cmero, Daniel Rosebrock, Steven E. Schumacher, Yu Fan, Matthew Fittall, Ruben M. Drews, Xiaotong Yao, Thomas B.K. Watkins, Juhee Lee, Matthias Schlesner, Hongtu Zhu, David J. Adams, Nicholas McGranahan, Charles Swanton, Gad Getz, Paul C. Boutros, Marcin Imielinski, Rameen Beroukhim, S. Cenk Sahinalp, Yuan Ji, Martin Peifer, Inigo Martincorena, Florian Markowetz, Ville Mustonen, Ke Yuan, Moritz Gerstung, Paul T. Spellman, Wenyi Wang, Quaid D. Morris, David C. Wedge, Peter Van Loo, and on behalf of the PCAWG Evolution and Heterogeneity Working Group and the PCAWG Consortium

Methods S1 – Supporting figures and tables, related to STAR methods

METHODS S1 – FIGURES AND TABLES, RELATED TO STAR METHODS 3
Dataset 3
Copy number consensus 5
JaBbA 5
Copy number consensus approach 6
Copy-number-calling methods differ in genome segmentation 6
Method for determining consensus segment breakpoints 7
Most consensus breakpoints obtain support from SVs 9
Resolving whole genome duplication uncertainty 10
Subclonal architecture methods 12
BayClone-C 12
Ccube 12
cloneHD 12
CTPsingle 14
DPClust 15
PhylogicNDT 15
Subclonal architecture consensus approaches 16
Consensus approaches 16
Weme (WM – Weighted Median) 17
Results 18
Validation of subclonal reconstruction 24
Simulation of subclonally heterogeneous samples – PhylogicSim500 24
A grid approach to simulations of tumors – SimClone1000 25
Results of reconstructing the subclonal architecture of tumors 26
Results by individual methods are consistent with consensus results 26
Selection and driver genes 27
Subclonal driver genes and their unique sequence 27

Gene set analysis of subclonally mutated genes 27
Tracking signature activities across cancer timelines 28
SV analysis and fusion clonality detection 29
Clonality analysis of recurrent structural variants 29

METHODS S1 – FIGURES AND TABLES, RELATED TO STAR METHODS

Here we have collected figures and tables referenced in STAR methods - method details.

Dataset

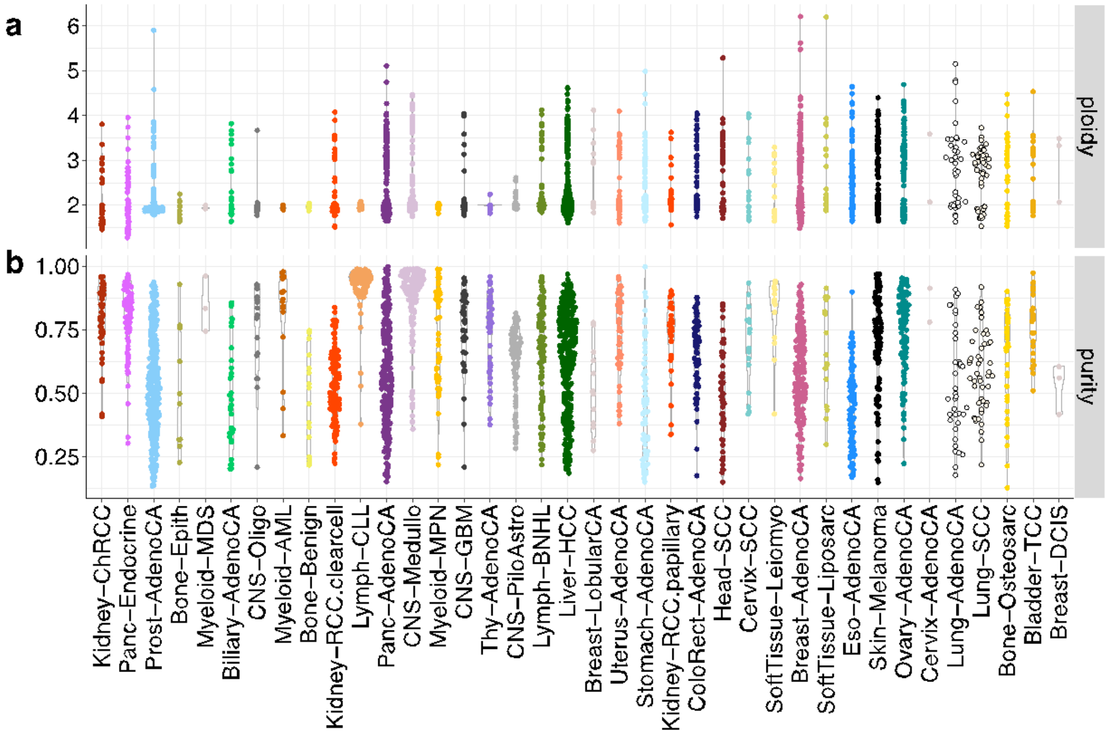

Fig. 1 Ploidy (a) and purity (b) values across cancer types, sorted by median ploidy.

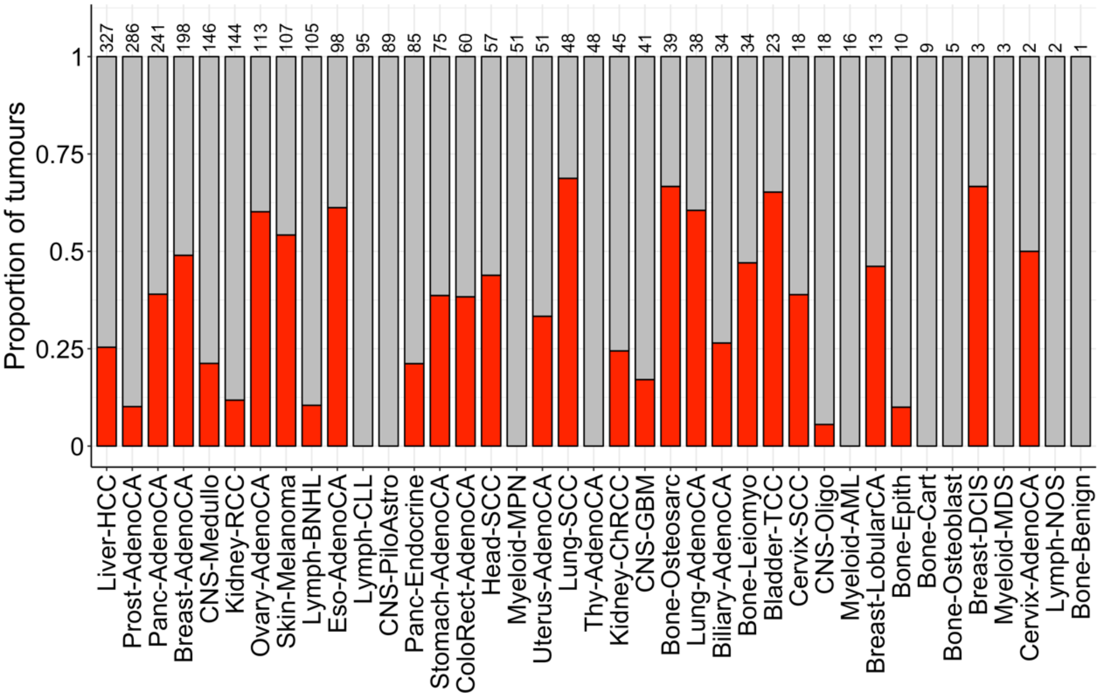

Fig. 2 Proportion of tumors with a whole genome duplication per cancer type.

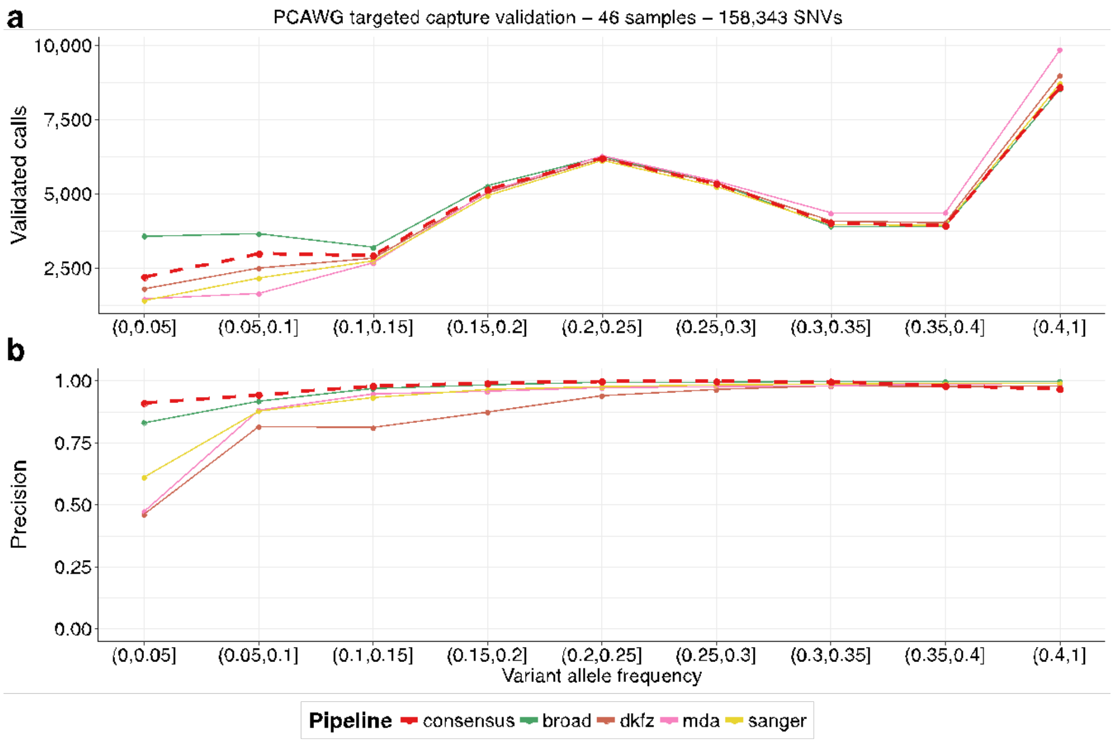

Fig. 3 ***SNV validation results.*** *The figure shows the number of validated calls for each variant allele frequency bin (a) and the obtained precision (b). The consensus achieves the highest precision of all pipelines with a minimum of 90% of positive calls (lowest VAF bin) and over 94% in all other VAF bins.*

Copy number consensus

JaBbA

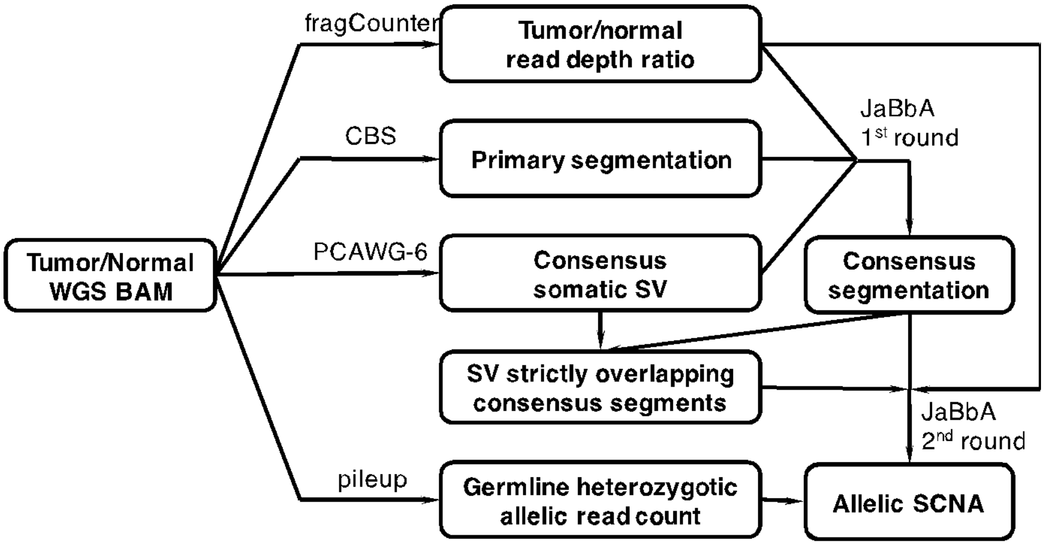

Fig. 4 The full JaBbA pipeline depicted graphically.

Copy number consensus approach

Copy-number-calling methods differ in genome segmentation

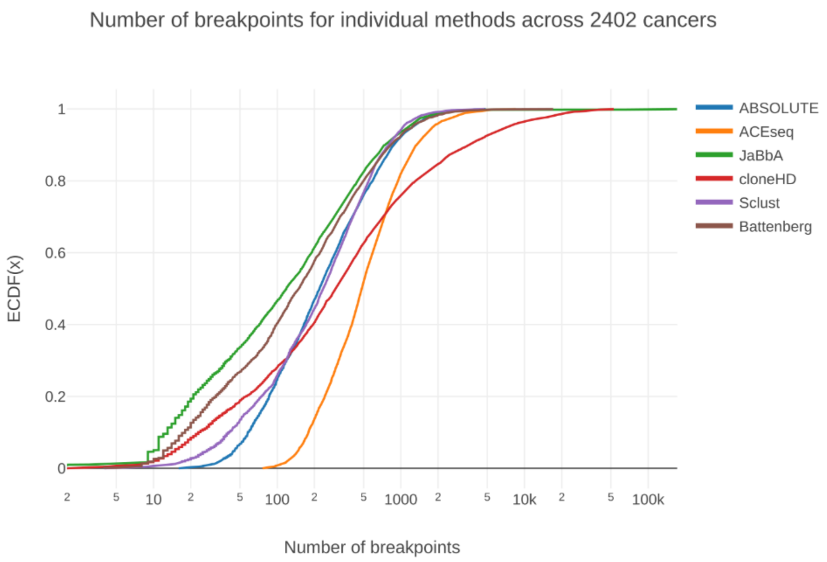

Fig. 5 Cumulative distribution of the number of samples with the indicated number of breakpoints predicted for each tumor by the six copy-number-calling methods. ACEseq and cloneHD (the "liberal" methods) often predicted an order of magnitude more breakpoints per tumor than ABSOLUTE, JaBbA, Sclust, and Battenberg (the "conservative" methods). While the four conservative methods characterized only approximately 7% of cancers as having more than 1000 breakpoints, ACEseq and cloneHD found 17% and 24%, respectively, crossing this threshold.

Method for determining consensus segment breakpoints

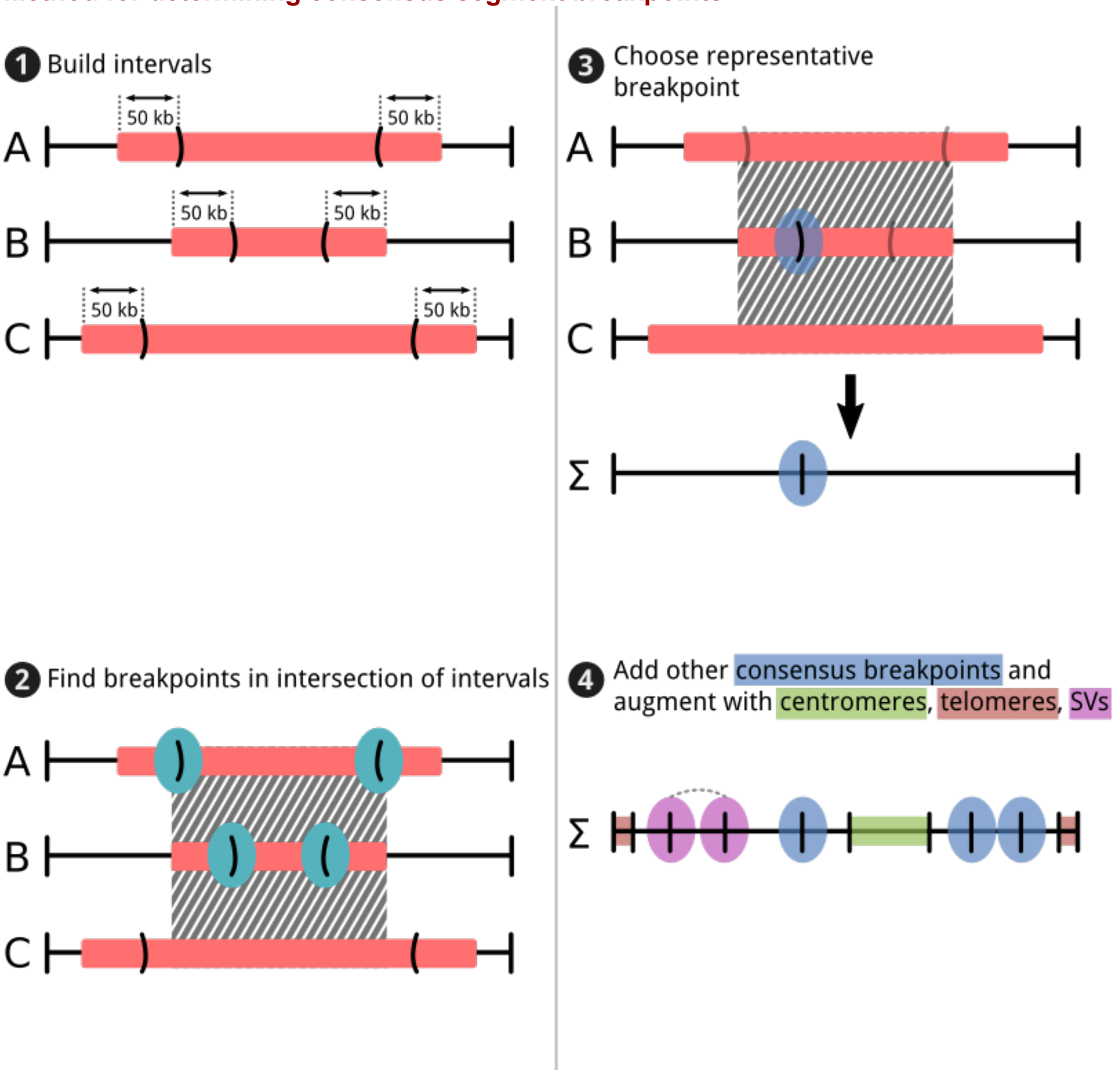

Fig. 6 Individual genome segmentation methods A, B, and C report segments of constant copy number. In this example, we determine the consensus segmentation Σ, requiring that all three methods A, B, and C contribute to the intersection that yields consensus breakpoints. Each segment from methods A, B, and C is composed of a start locus S_i and end locus E_i. Depicted here for each method is the end point of one segment, E_i, and the start point of the next segment, S_{i+1}. Note that the methods may differ in the copy number state they assign to each segment, as the consensus breakpoint method is concerned only with where segments occur, not what the status of each segment is. By proceeding through steps one to three, the consensus breakpoint algorithm selects a single breakpoint supported by the input segments from the individual methods. In step four, the breakpoints supported by the individual segmentation methods are refined and augmented using the PCAWG consensus structural variants, as well as knowledge of where centromeres and telomeres occur on each chromosome.

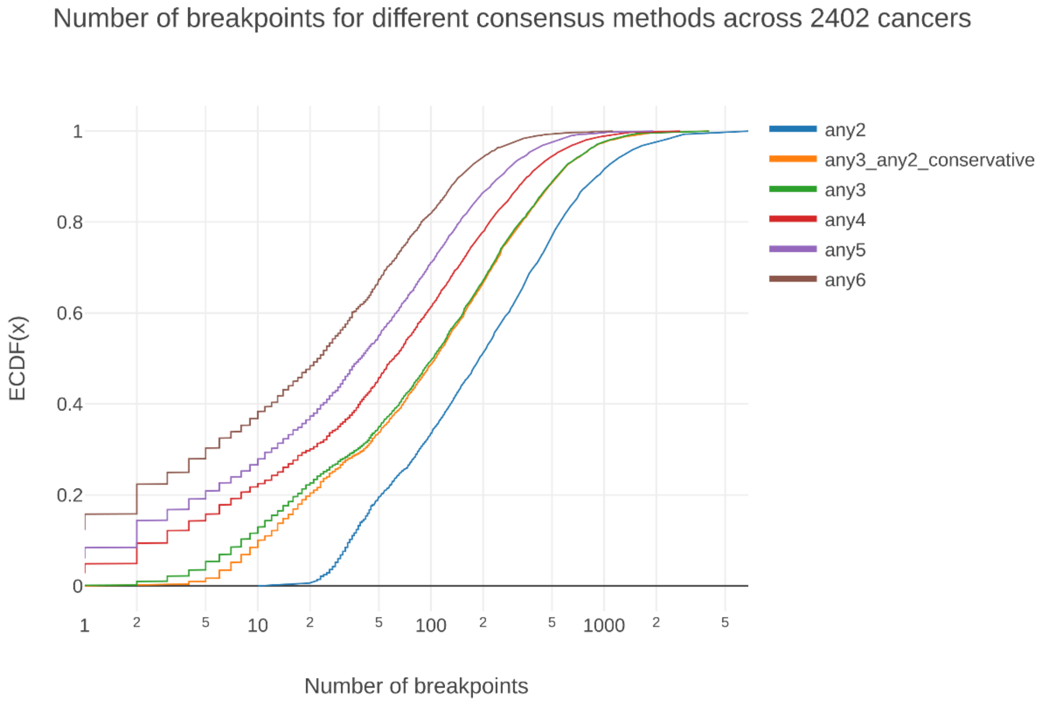

Fig. 7 Cumulative distribution of the number of samples with the indicated number of breakpoints produced for each cancer by different consensus strategies. Given the six copy-number-calling methods, the anyN strategies required N of six methods to agree on a breakpoint's placement to establish a consensus breakpoint at that location. The strategy we selected, any3_any2_conservative, required agreement between any three methods, or agreement between any two of the four "conservative" methods (i.e., ABSOLUTE, Battenberg, JaBbA, and Sclust). This avoided calling consensus breakpoints supported by only the two "liberal" methods (ACEseq and cloneHD). Relative to any3, the any3_any2_conservative strategy introduced only a few extra breakpoints, but corrected false negatives where we failed to obtain support for a breakpoint from three methods despite clear evidence of its existence in the underlying data.

Most consensus breakpoints obtain support from SVs

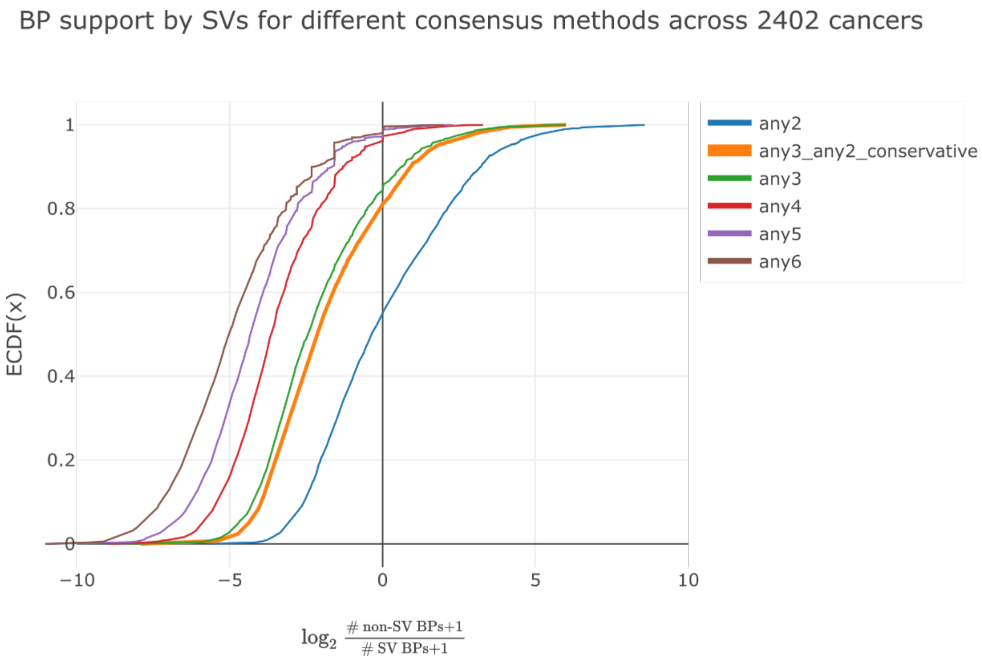

Fig. 8 Cumulative distribution across cancers of the log-ratio of the number of breakpoints without support from SVs, to the number of breakpoints with SV support. As virtually all copy number events should generate associated structural variants, most consensus breakpoints should be able to find a nearby supporting SV. Under the any3_any2_conservative consensus breakpoint strategy, only 20% of cancers had more unsupported breakpoints than SV-supported breakpoints (i.e., a log ratio greater than zero).

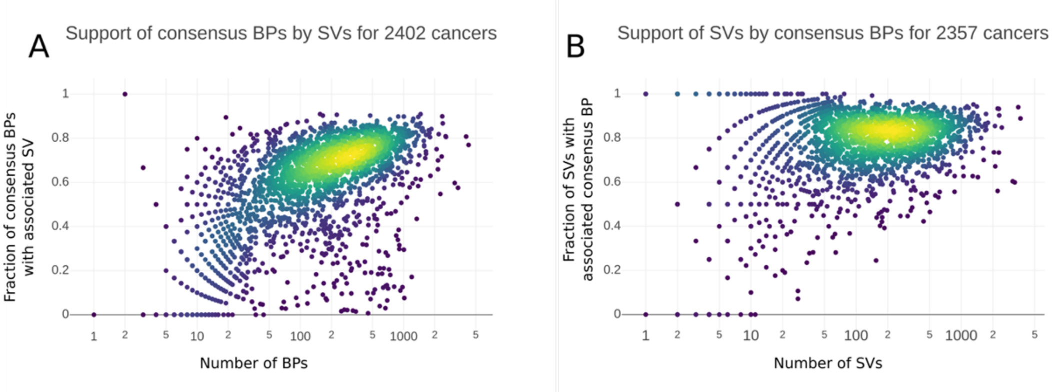

Fig. 9 ***A)*** *Support of consensus breakpoints (BPs) from SVs as a function of number of BPs. Color indicates density. As the number of BPs increased, so too did the fraction of BPs that found a nearby supporting SV. The mean fraction of BPs with SV support was 77%.* ***B)*** *Support of SVs from consensus BPs as a function of number of SVs. Color indicates density. There was no correlation between the number of SVs and the fraction with support from BPs. The mean fraction of SVs with BP support was 83%.*

Resolving whole genome duplication uncertainty

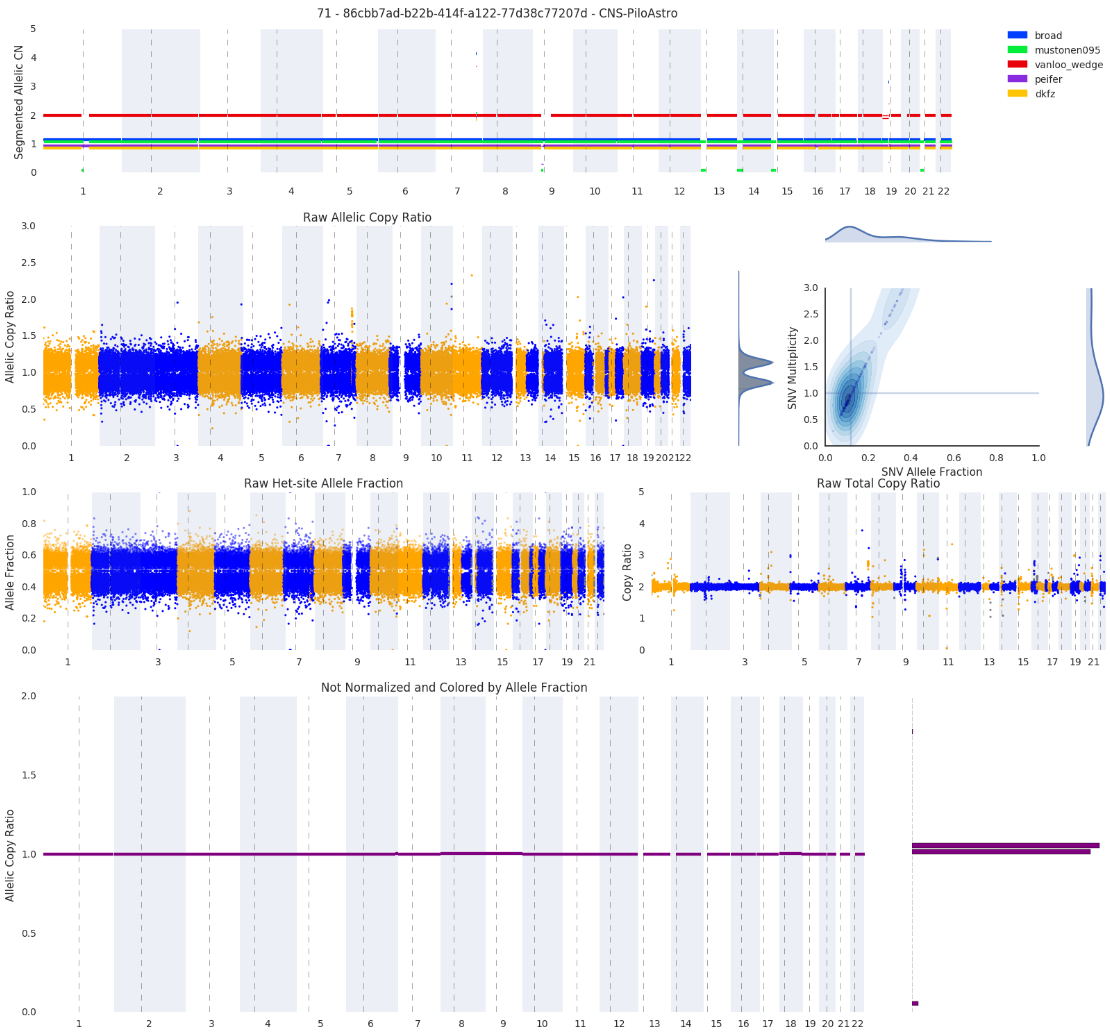

Fig. 10 Copy number review figure for sample SA414133. The figure contains copy number profiles for five methods (top), raw allele ratio's for SNPs and multiplicity values (second row left and right respectively), raw B allele frequencies and copy ratio's for SNPs (third row left and right) and normalized allele ratios (bottom). In this case the raw data shows there are no copy number alterations of note. Battenberg however (red copy number profile) calls a whole genome duplication. Adding the duplication does not allow for an increase in the proportion of the genome called with clonal copy number. In this scenario Battenberg was considered the outlier and the profile is flagged as no_WGD. This figure shows copy number profiles on the methods' own segmentation.

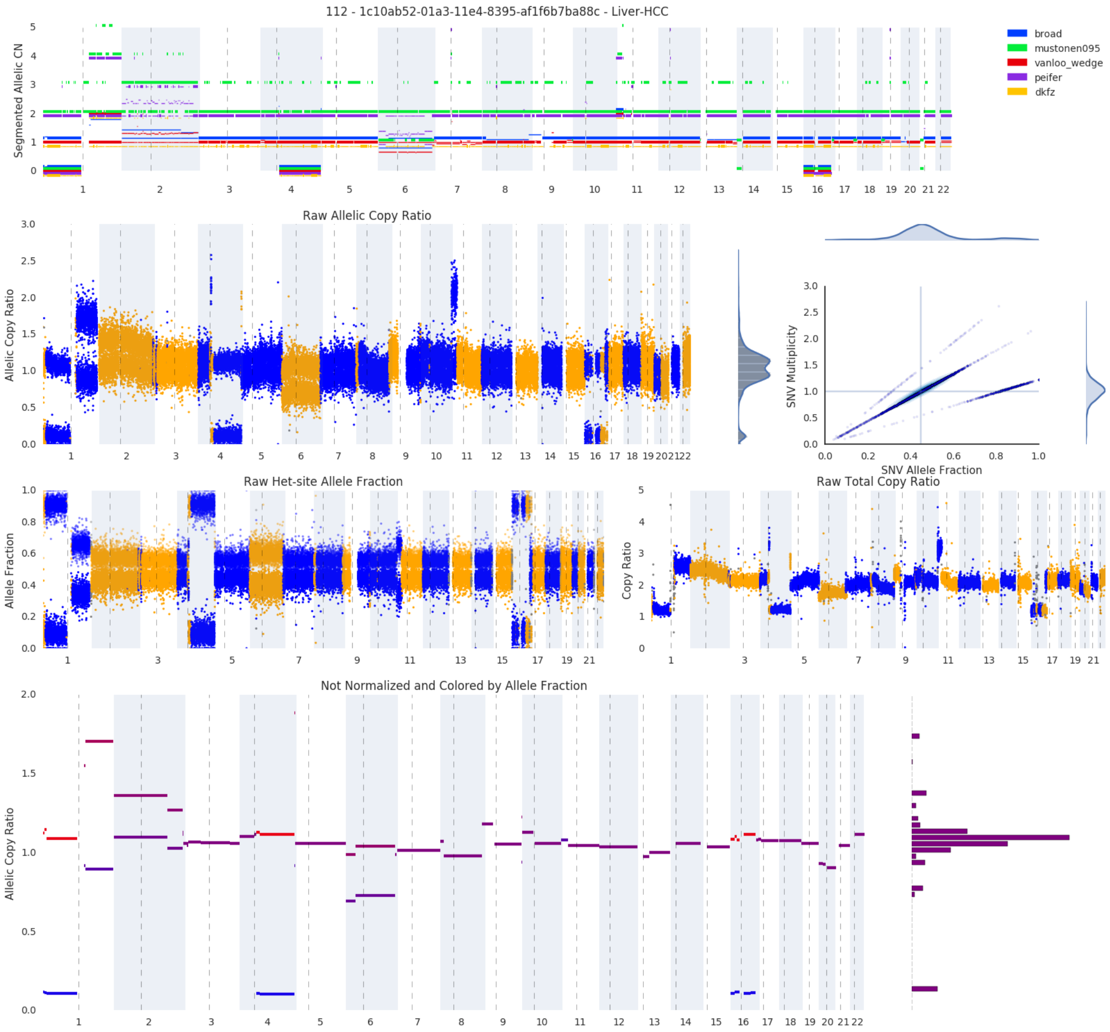

Fig. 11 Example of copy number profile where noise affects the fit and causes disagreement on the ploidy. The copy number profiles called by cloneHD (green), Sclust (purple) and ACEseq (yellow) contain large numbers of small segments, compared to ABSOLUTE (blue) and Battenberg (red). The methods affected by the noise interpret it as signal, which cause it to fit the segments with a higher or lower copy number state, resulting in a ploidy discrepancy. This figure shows copy number profiles on the methods' own segmentation. The call for this sample (SA529805) is no_WGD.

Subclonal architecture methods

BayClone-C

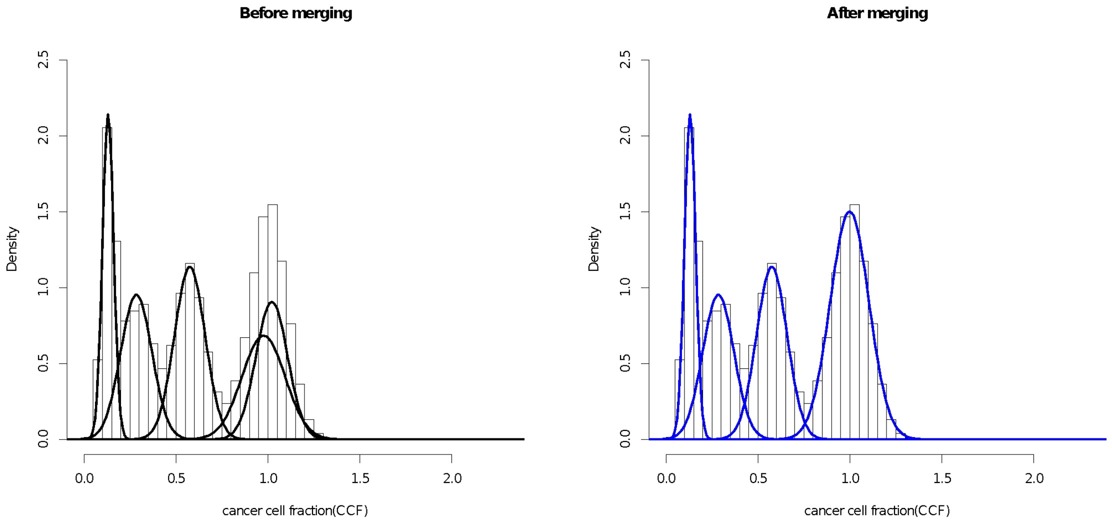

Fig. 12 An example of merging using a PCAWG sample SA6164. Left and right panels are CCF clusters before and after merging of Gaussian components.

Ccube

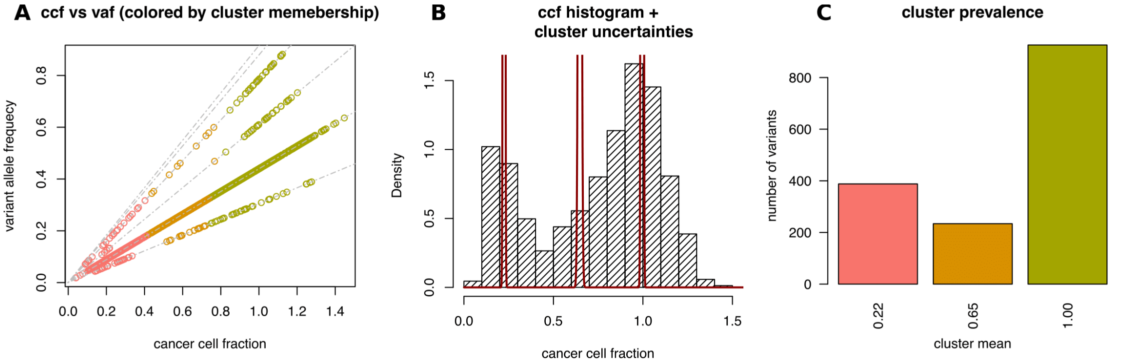

Fig. 13 Ccube results summary for sample SA518422. ***A)*** *Scatterplot of VAF and CCF. Each point in the figure is a mutation color coded by its cluster membership. The grey dashed lines are all possible linear mappings (eq. 1) determined by copy number and multiplicity configurations in the sample.* ***B)*** *Histogram of observed CCFs. The red solid line shows the approximated posterior distribution of CCF cluster centers. The peak at CCF=1 corresponds to the clonal cluster.* ***C)*** *The number of variants assigned to each CCF cluster. Each CCF cluster is labelled by its cluster center.*

cloneHD

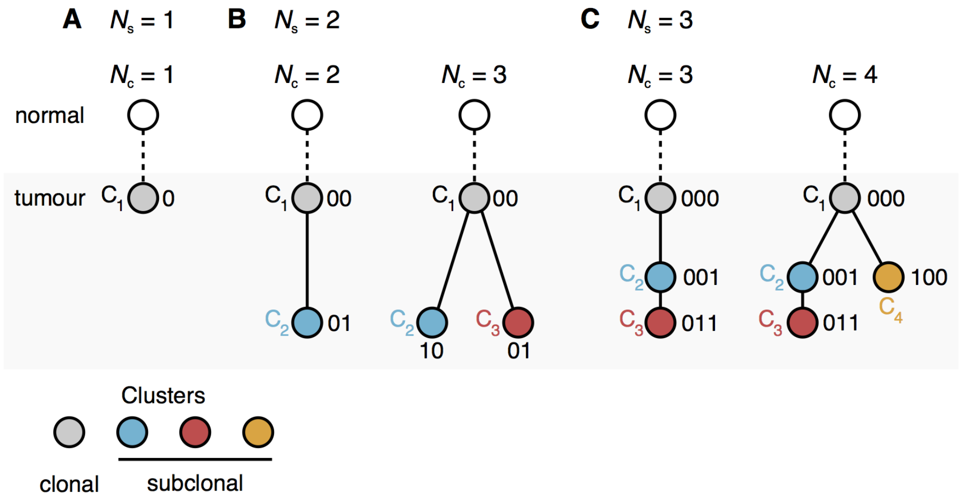

*Fig. 14 Mapping between subclone genotypes and clusters. There is a correspondence between subclonal genotypes and mutation clusters. Each node in a tree is a cluster, and mutations are acquired along the edges of the tree, from the root (top) to the leaves (bottom). Each node is labelled by the cluster genotype. The ancestral node linking to the clonal cluster is also shown. SNV genotype priors that are compliant with the infinite sites assumption constrain the set of tree topologies. The examples show families of trees compliant with this assumption relating **(A)** $N_s = 1$, **(B)** $N_s = 2$ and, **(C)** $N_s = 3$ genotypes, each of which contains N_c mutation clusters. $N_s = 1$ corresponds to a scenario where there are no subclones in the sample, so all mutations belong to the clonal cluster denoted as a grey node. The $N_s = 2$ scenario has a single subclone corresponding to one clonal and one or several subclonal clusters and $N_s = 3$ more than one subclone describing linear or branched evolution. For example, with $N_s = 2$ subclones, genotype $g = 00$ denotes all clonal mutations, $g = 10$ mutations that are private to the blue cluster and $g = 01$ mutations that are private to the red cluster. The tree prior extends cloneHD by inferring a prior for a mutation to belong to one of the clusters shown. Assuming a uniform mutation rate the prior weights can be interpreted to relate to the branch lengths of the tree leading to each node.*

CTPsingle

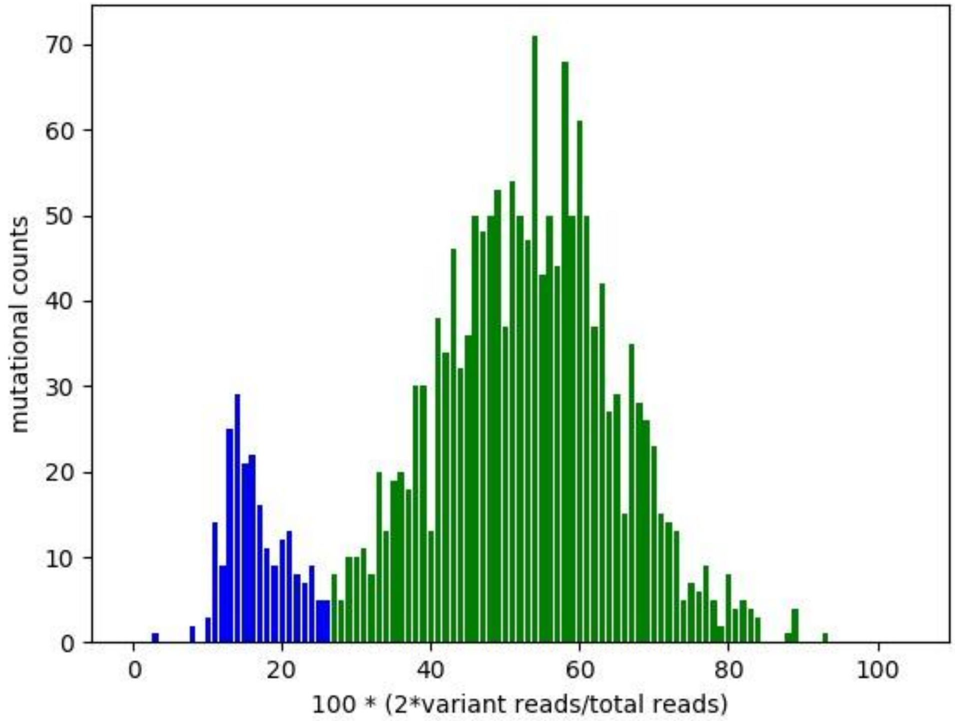

Fig. 15 Barplot of CTPsingle clustering result for sample SA530652. Different colors represent different subclones. Although clustering is performed in read counts space, for the sake of visualization, mutations are merged based on the ratios of variant and total reads shown on the x-axis and scaled to the interval [0,100]. For each integral value on the x-axis the number of mutations having the considered ratio is shown on the y-axis.

DPClust

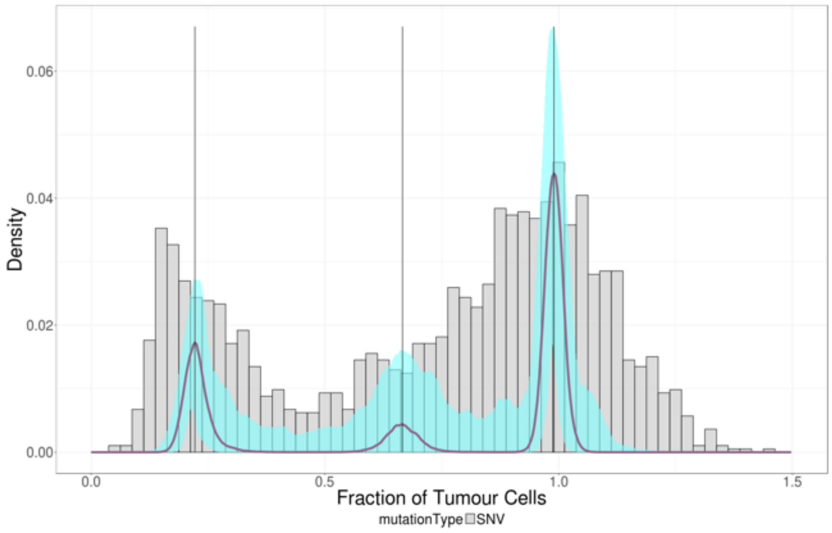

Fig. 16 Posterior density (purple) and confidence interval (cyan) of cluster locations in cancer cell fraction (CCF) space for sample SA518422. The number of mutation clusters is obtained by finding peaks in the density. The peak at CCF=1 represents the clonal SNVs carried by all tumor cells, the other two peaks represent two subclones.

PhylogicNDT

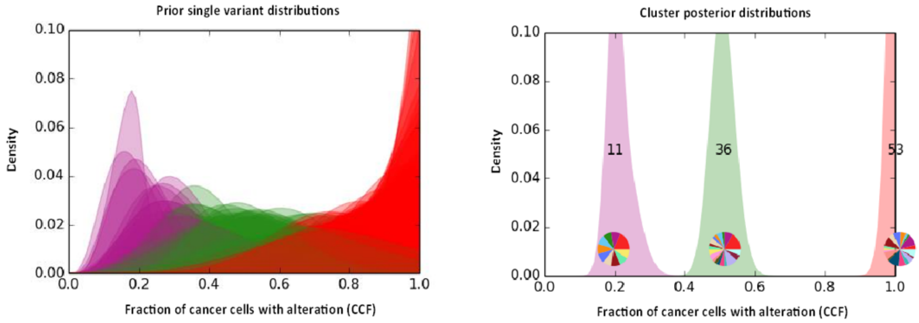

Fig. 17 ***PhylogicNDT clustering.*** *The prior mutational CCF distributions (left) and posterior cluster densities (right). Colors represent the final subclonal assignment of mutations. Pie charts show distribution of clustered mutations across chromosomes.*

Subclonal architecture consensus approaches

Consensus approaches

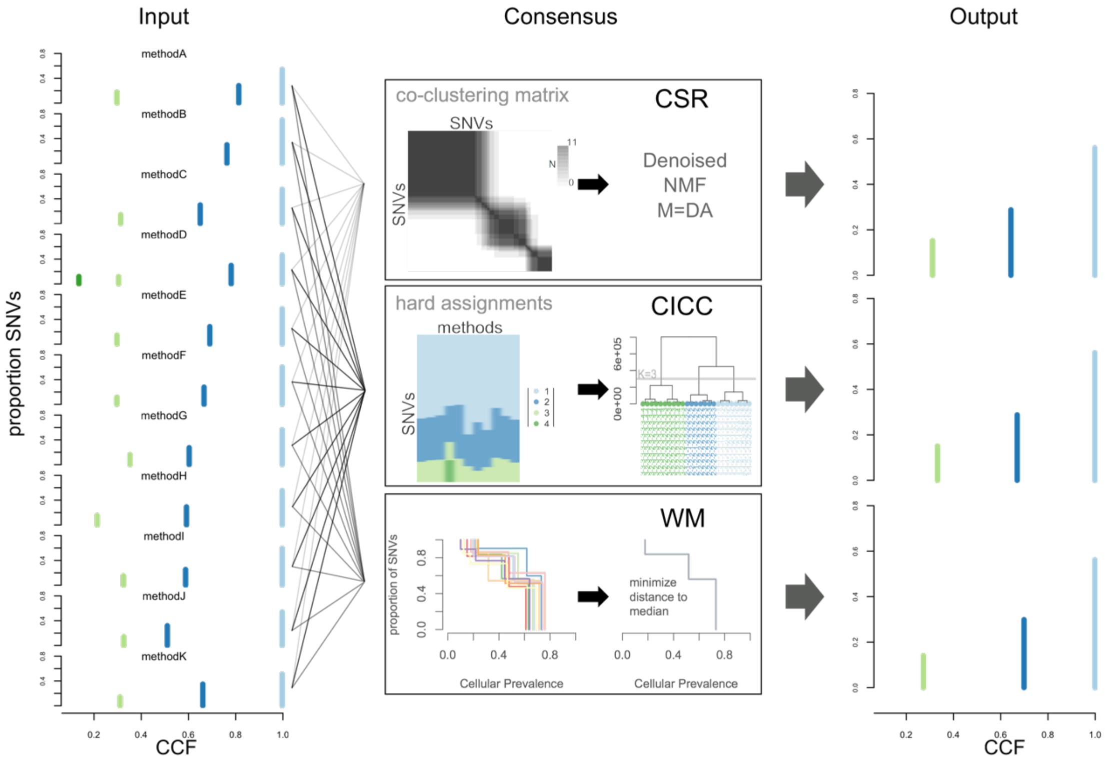

Fig. 18 Workflow of the consensus approaches. ***Input.*** *On the left, inputs are represented by their respective solutions in the space defined by CCF and proportion of SNVs of the clusters. Each cluster is represented by a vertical colored bar. Each of the 11 methods also provides mutation assignments corresponding to these solutions.* ***Consensus.*** *In the middle panels, illustration of the three consensus approaches. They take different aspects of the individual methods as input and obtain the consensus from it in different ways (see main text).* ***Output.*** *Right panels, the output solutions of the three consensus methods in the same space as Input. Both CSR and CICC also provide mutation assignments to the clusters, not shown in that space and not used by MutationTimer.*

Weme (WM – Weighted Median)

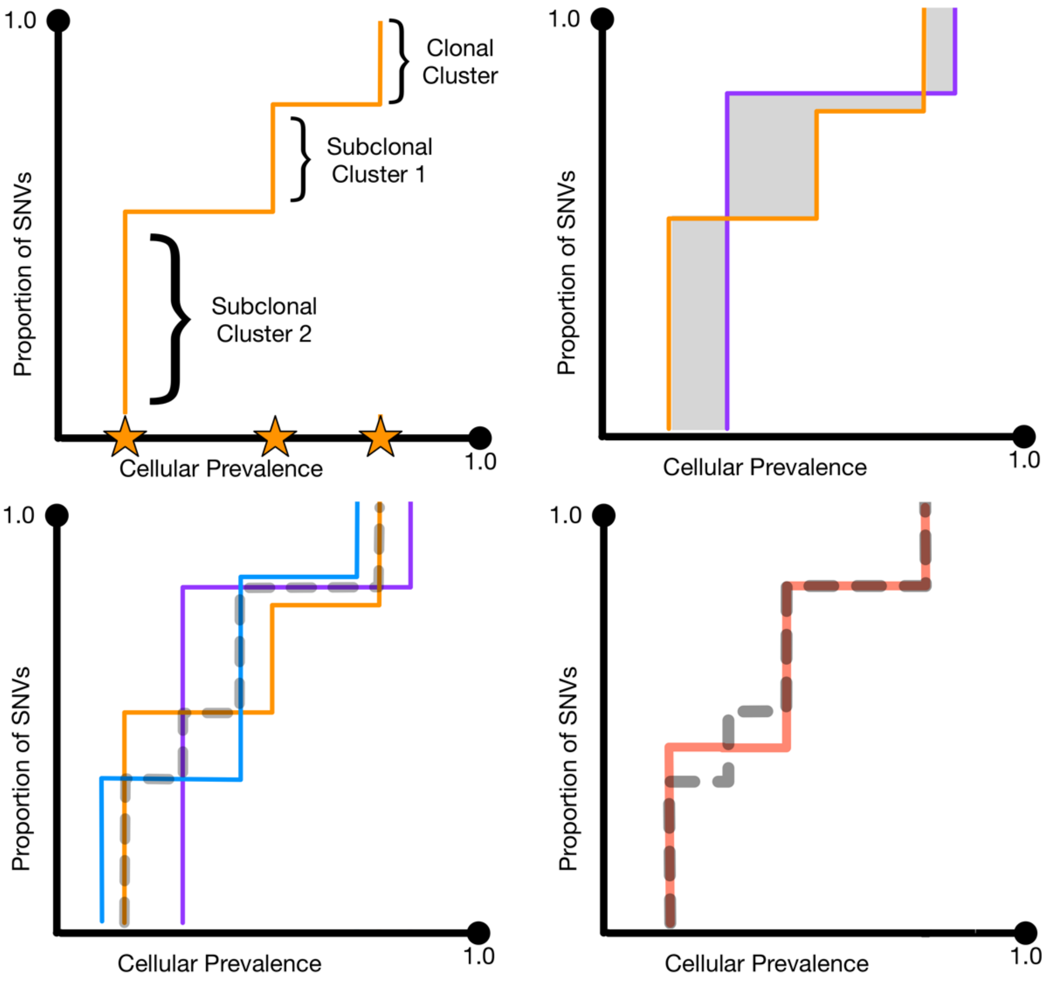

Fig. 19 ***Illustration of the WeMe algorithm****. (Top left) CDF representation of a 1-d "clustering". Stars indicate the cellular prevalence of the cluster centers; height of the CDF jumps corresponds to the proportion of mutations assigned to that cluster. (Top right) Two CDFs (orange and purple lines) indicate different clusterings of the same sample. Total area of grey region is equal to the Earth Mover Distance (EMD), also known as Wasserstein distance, between the two clusterings. (Bottom left) Three solid lines (orange, blue, purple) are CDFs for three different clusterings. The median clustering (dashed grey line) corresponds to the CDF made by taking the median in the x-axis direction of the three CDFs for each y-value. Note that the median clustering has four cluster centers. (Bottom right) The weighted median clustering corresponds to the CDF (red line) with the desired number of centers (i.e., jumps) that minimizes the EMD to the CDF for the median clustering (dashed grey line).*

Results

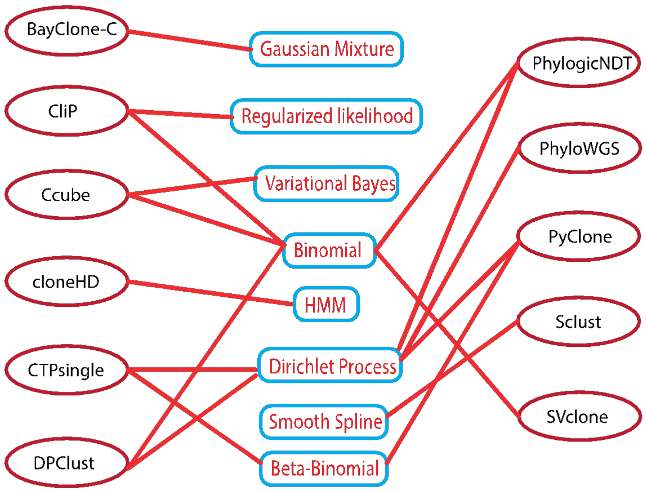

Fig. 20 Individual methods and their internal assumptions and models. Each method typically does not share assumptions/mechanisms with other methods. Even if they do, like PhylogicNDT and DPClust, they utilize the mechanism differently and there are substantial differences in pre-/post- processing.

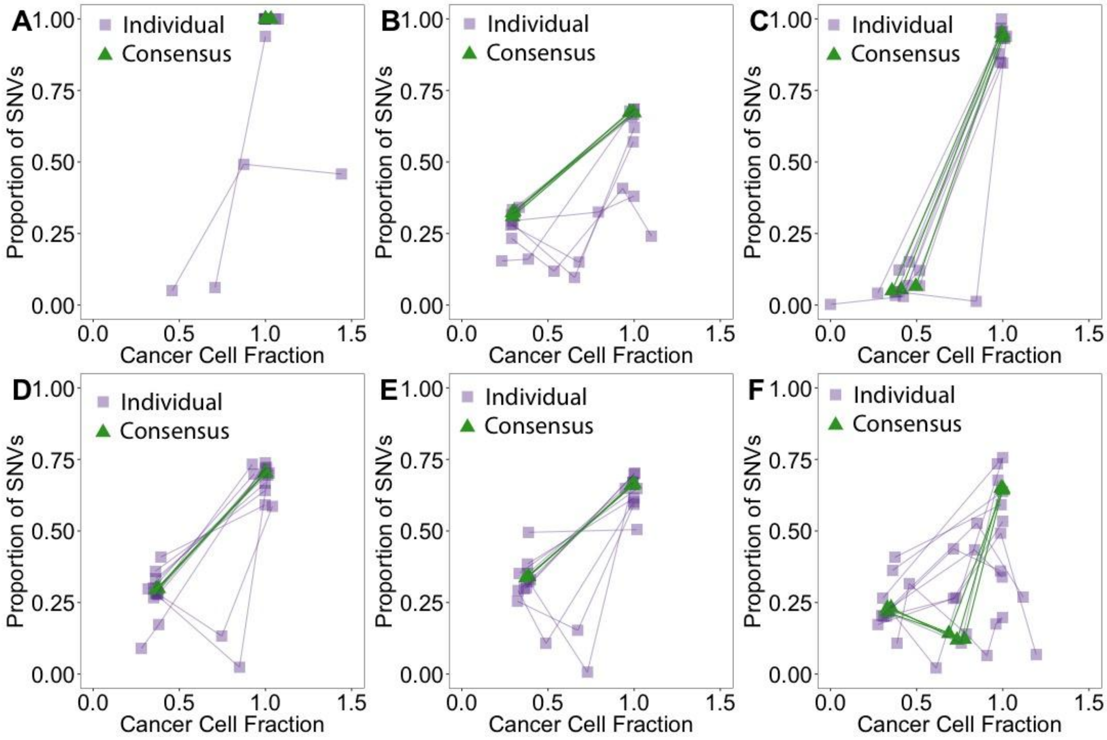

Fig. 21 Examples of subclonal reconstruction differences across methods. Results across methods are variable, but the consensus methods are able to reconstruct the subclonal structure robustly. Each individual method's subclonal peaks are linked with a line. A) Most solutions agree except two. B) Methods agree on the clonal location and size, but they diverge for the subclonal position and size. C-E) Global agreement on the number of clusters but deviation in position and size. F) High variability and disagreement across methods still leads to stable consensus solutions.

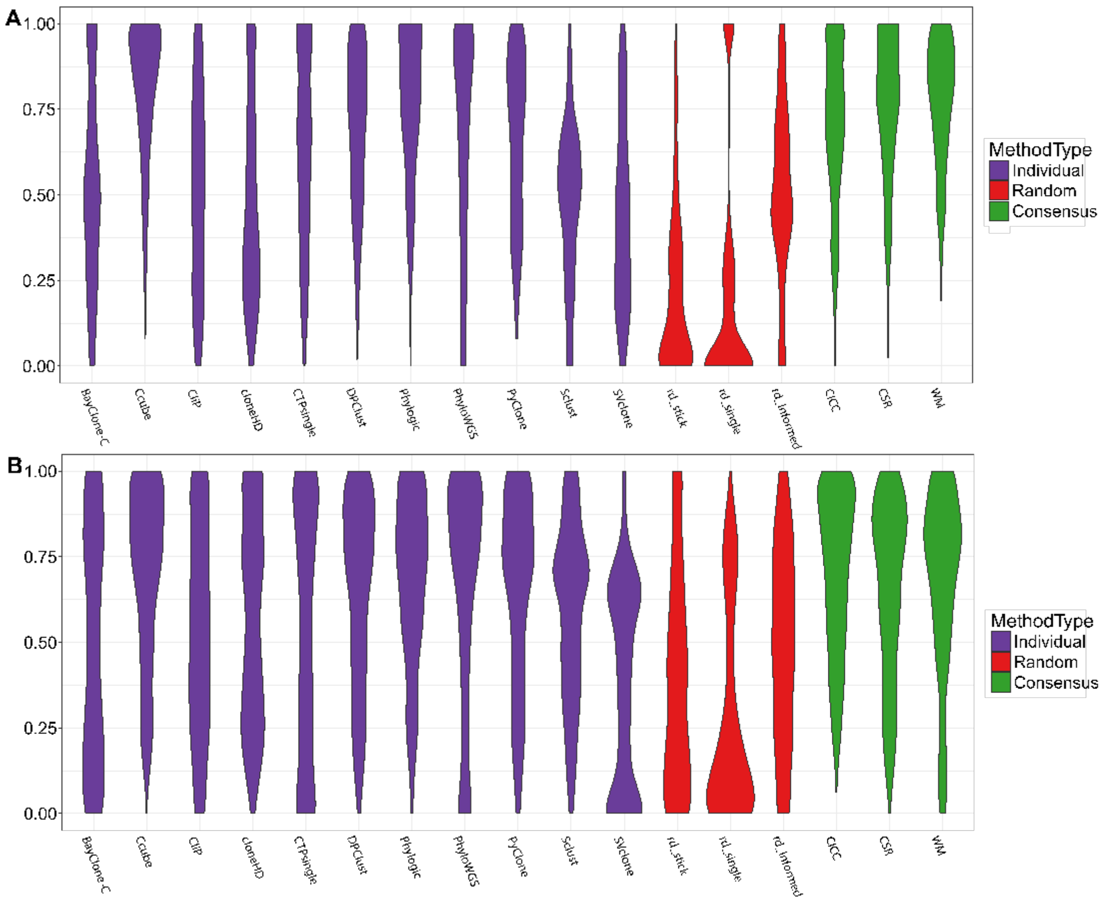

Fig. 22 The overall scores of all individual (purple), random (red), consensus (green) subclonal architecture reconstruction methods on A) PhylogicSim500 and B) SimClone1000.

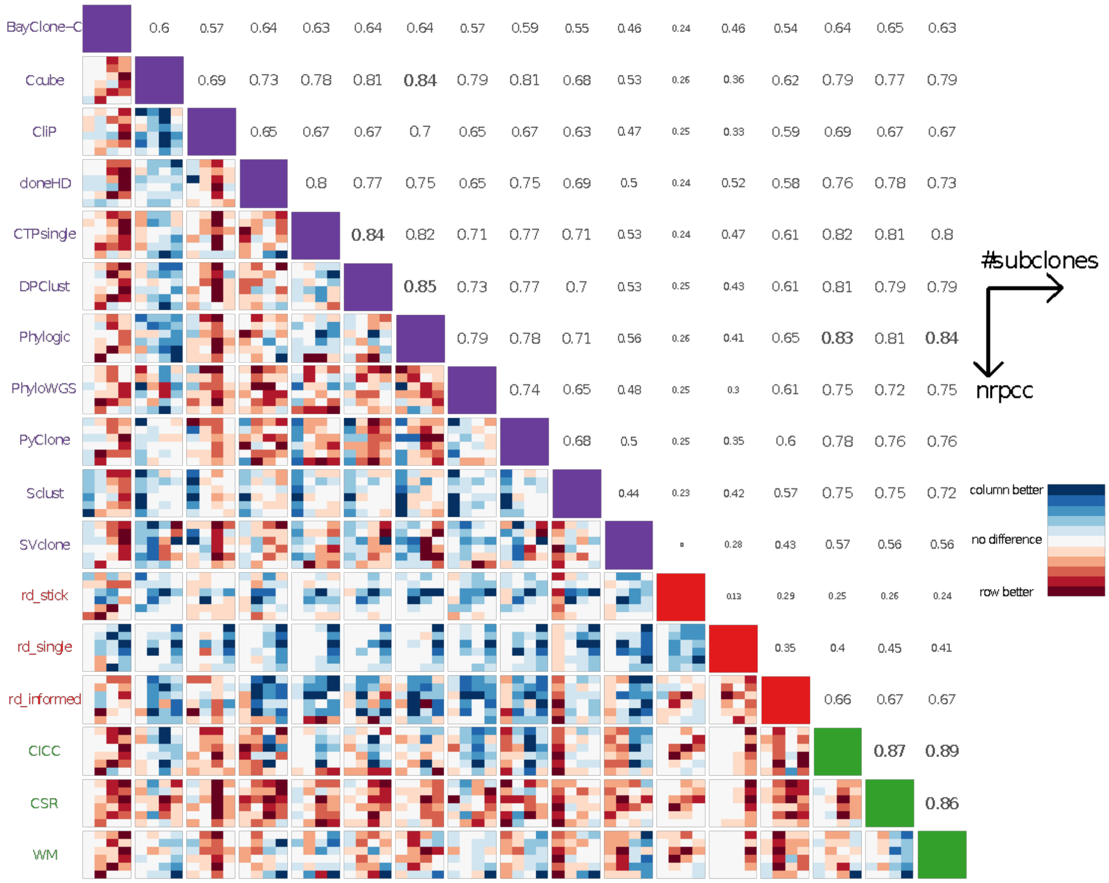

Fig. 23 Upper triangle: relative similarities between methods (individual methods are in purple, random methods are in red and consensus methods are in green). The size and transparency scale linearly with the similarity score. Lower triangle: heatmaps of relative ranks across simulations according to two grid parameters: number of subclones on the x-axis of each heatmap, nrpcc on the y-axis of each heatmap (the legend for these axes is on the top right). The scores are average across all simulations falling into the grid cell and a minmax normalization is then applied across the grid values, cells are then colored from red (method on the row is better) to blue (method on the column is better) with the intensity of the color scaling with the normalized score.

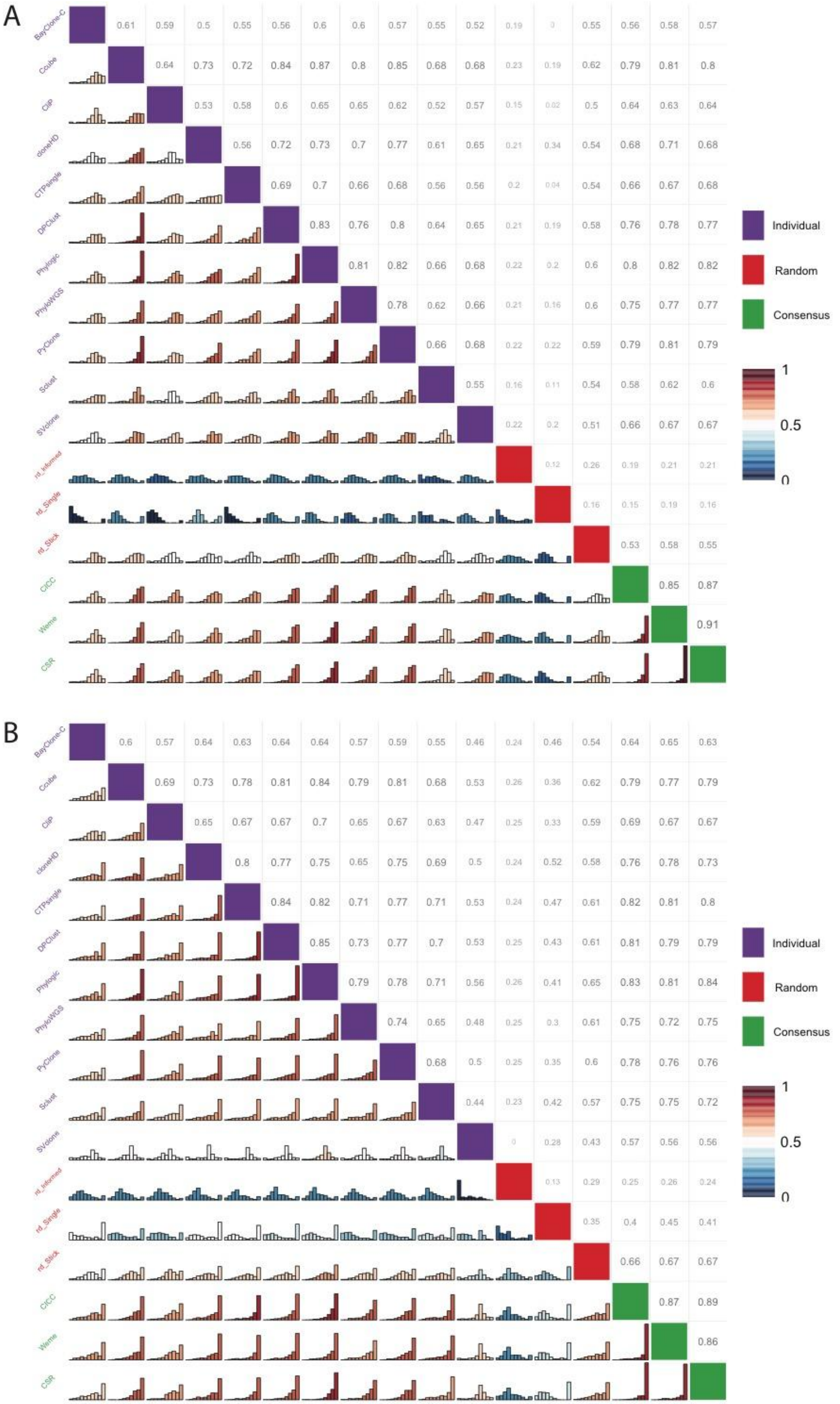
A
B
BayClone-C
Ccube
CliP
cloneHD
CTPsingle
DPClust
Phylogic
PhyloWGS
PyClone
Sclust
SVclone
rd_informed
rd_Single
rd_Stick
CICC
Wemme
CSR
Individual
Random
Consensus
1
0.5
0

Fig. 24 Pairwise distributions of the similarities across samples (lower triangle; bar width was set to 10%) and median similarities across samples (upper triangle) for A) PhylogicSim500 and B) SimClone1000.

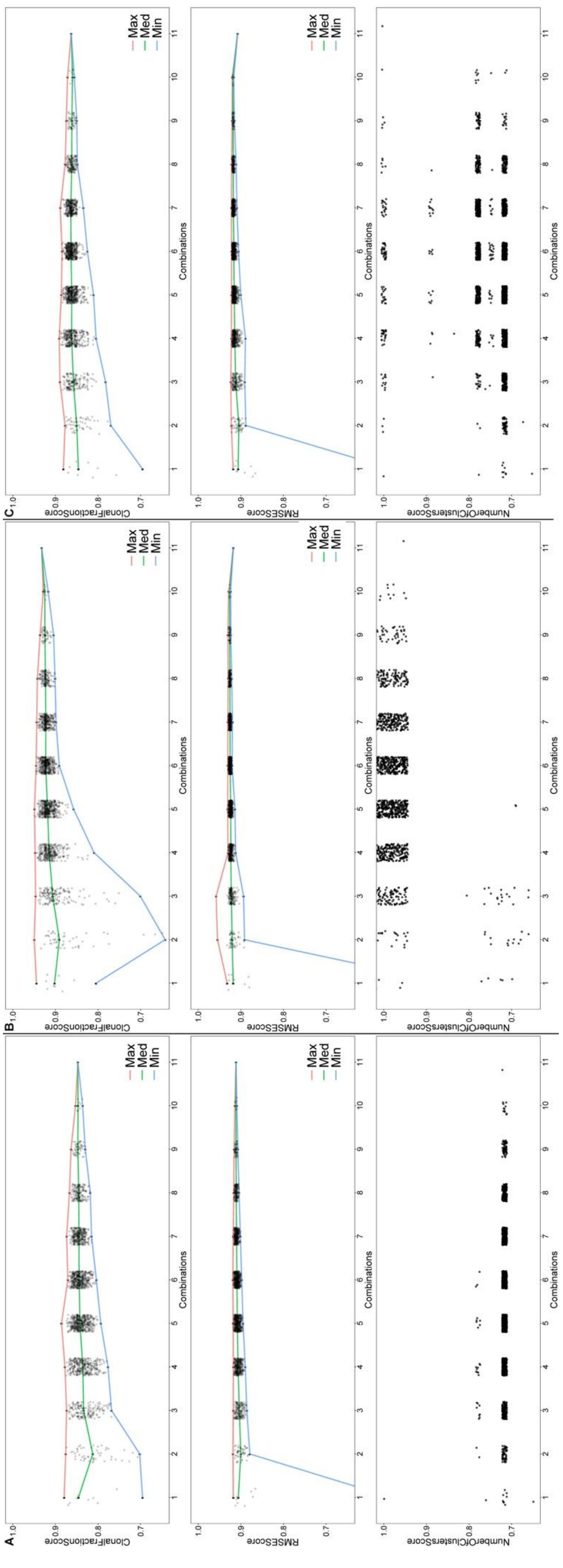
A
B
C
ClonalFractionScore
RMSEScore
NumberOfClustersScore
Combinations
Max
Med
Min

Fig. 25 Median performances of consensus methods on SimClone1000 with different numbers of methods included, from 1 (individual methods) to 11 (the reported consensus results), for clonal fraction, number of subclones and RMSE, from top to bottom. The green line connects the medians of the medians; the red and blue connect the extreme top and bottom values, respectively. Consensus was obtained using CSR (A), CICC on a subset of 680 out of 965 samples (B) or WM (C).

Validation of subclonal reconstruction

Simulation of subclonally heterogeneous samples – PhylogicSim500

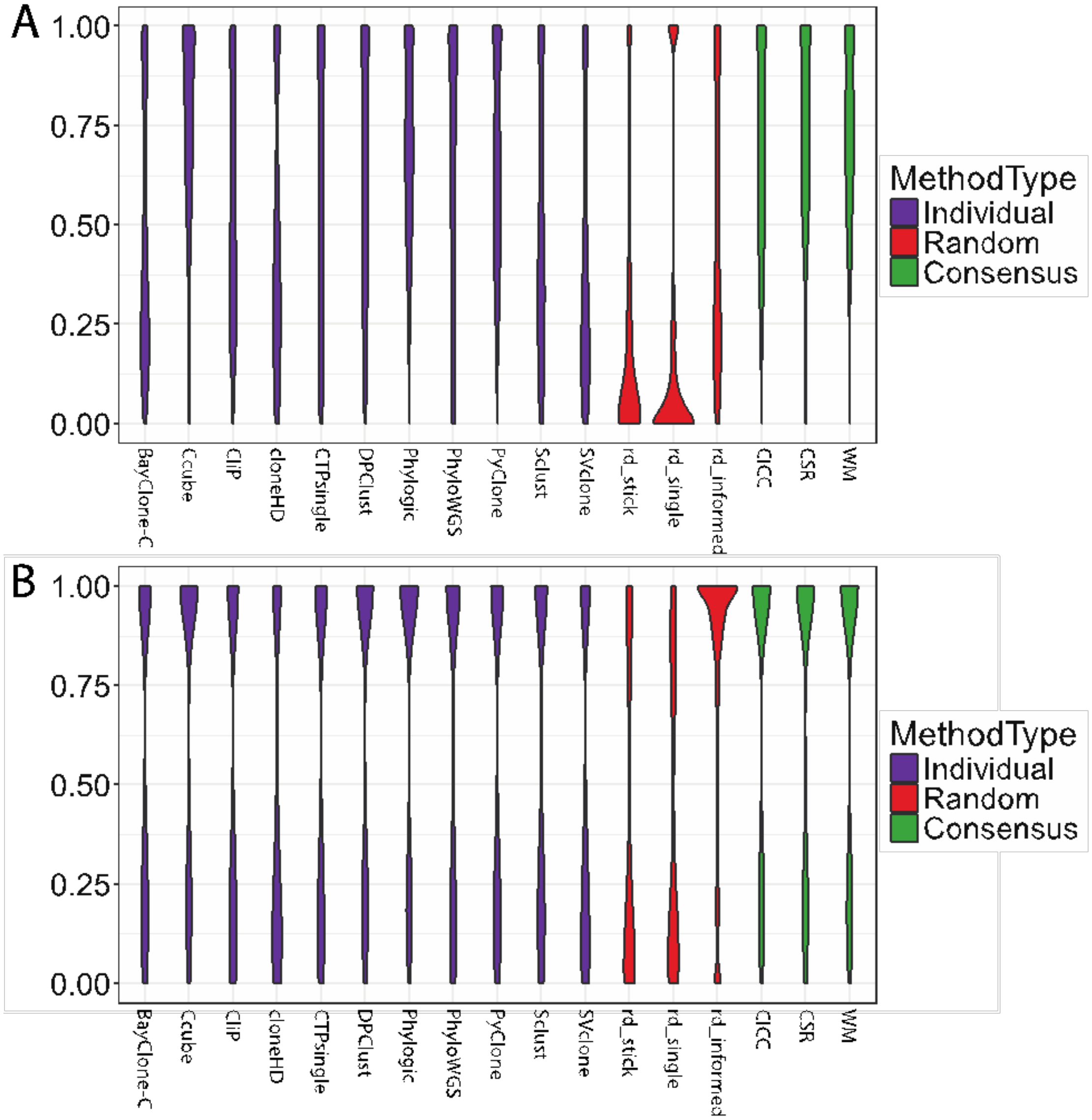

Fig. 26 Summary of subclonal reconstruction on simulation data. General consistency between individual methods and consensus method results: **A)** fraction of clonal mutations and **B)** number of subclonal clusters.

A grid approach to simulations of tumors – SimClone1000

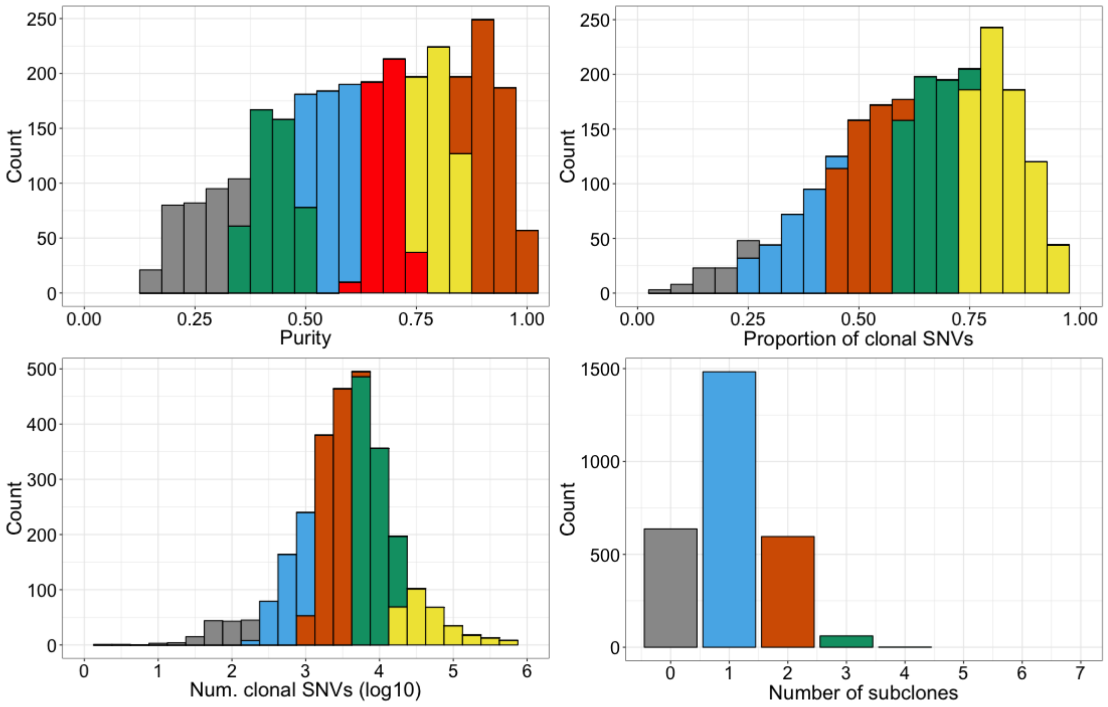

Fig. 27 The simulated data set was created as a grid with four axis. Each axis represents a type of measurement that can be obtained from real data. This figure shows the histogram of these four measurements from the PCAWG data and the colors represent bins along each grid axis. A simulated tumor falls somewhere on the grid, which amounts to a combination of 4 bins (one on each axis). The parameters for this sample are then generated by sampling a single value from each of the 4 bins.

Results of reconstructing the subclonal architecture of tumors

Results by individual methods are consistent with consensus results

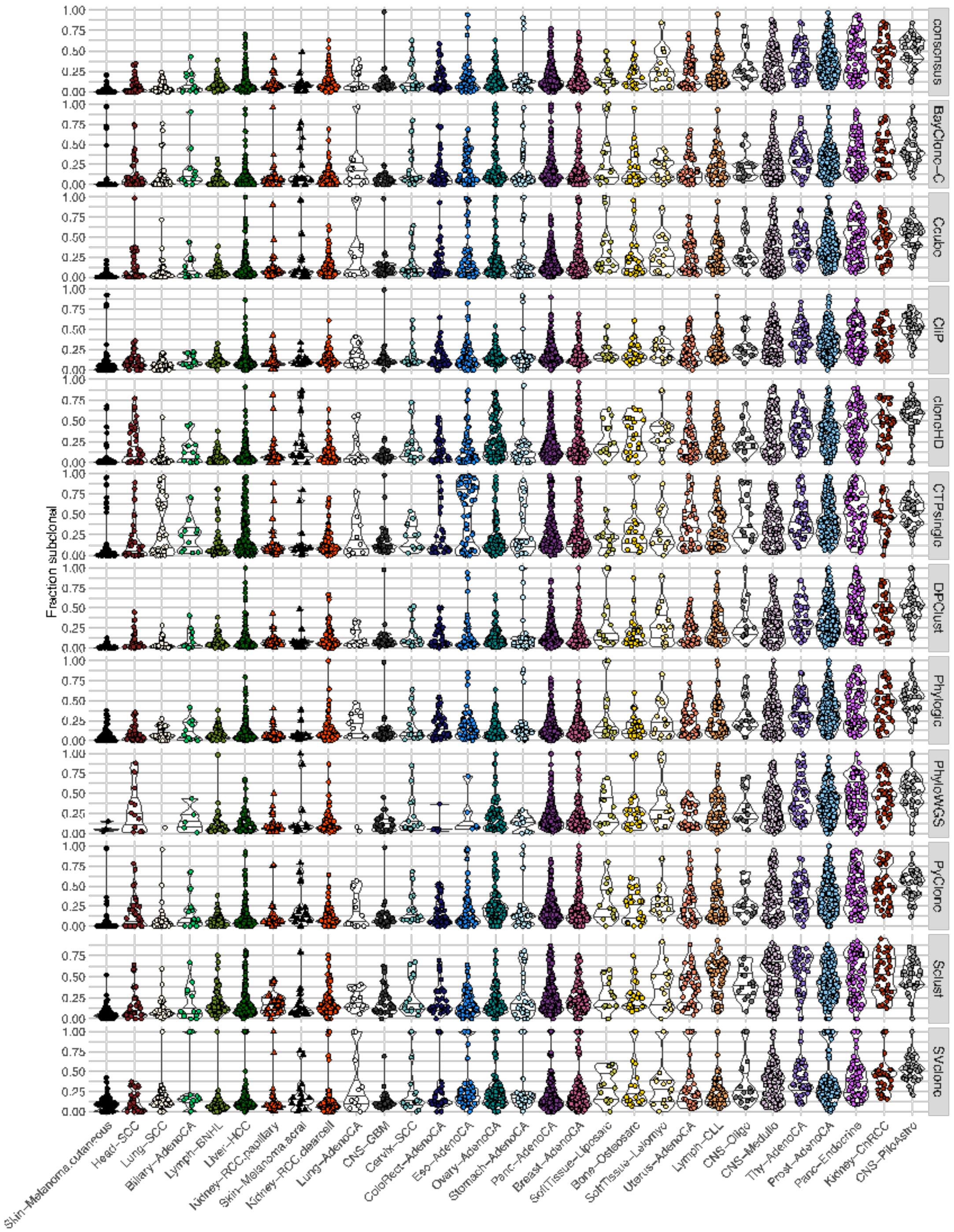

Fig. 28. Fraction of subclonal mutations for all eleven subclonal reconstruction methods and the consensus is shown for 1,705 samples with sufficient power to detect subclones at CCF > 30%. Samples have been limited to those with less than 2% tumor contamination in the matched normal sample and no activity of any of the identified artefact signatures (Alexandrov et al., 2020). Only representative

samples (The ICGC/TCGA Pan-Cancer Analysis of Whole Genomes Consortium, 2020) from multi-sample cases are shown. Cancer types are ordered by median fraction of subclonal mutations in the consensus reconstruction. The distributions of the fraction of subclonal mutations per cancer type, as determined by the individual methods, are very similar to the reported consensus architecture.*

Selection and driver genes

Subclonal driver genes and their unique sequence

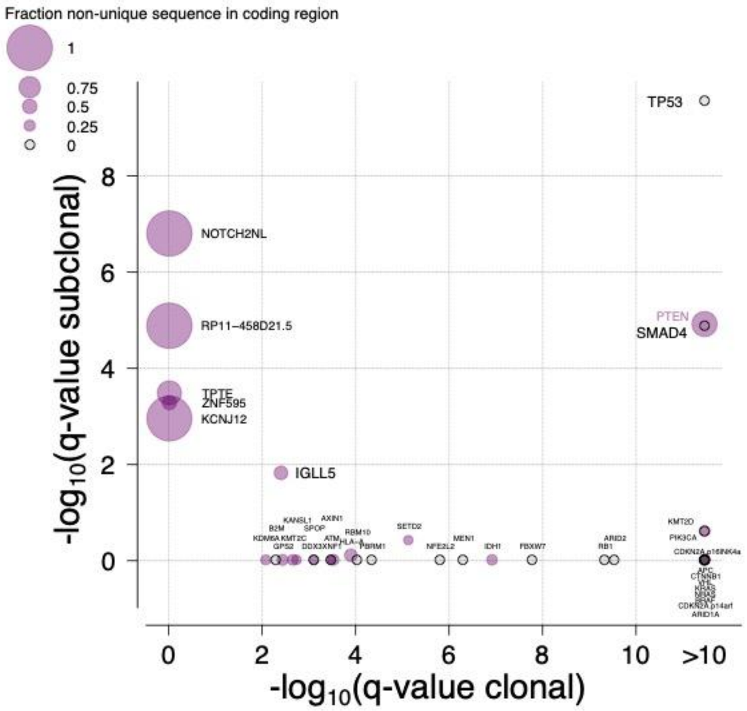

Fig. 29 ***Exclusively subclonal drivers might display lower VAF/CCF due to alignment ambiguities in their coding sequence.*** *Scatter plot of the -log10(q-values) of the dN/dS for clonal (x-axis) vs. subclonal (y-axis) mutations. Each point represents a gene and is colored if part of its sequence presents high similarity with another genomic region in the reference genome. The size is proportional to the fraction of the non-unique sequence in the gene coding region.*

Gene set analysis of subclonally mutated genes

Table 1 Table of the 10 top significant gene sets (q<0.1) in the subclonally mutated genes. The columns in order give: the gene set name, the number of genes in the gene set, the p-value of the hypergeometric test, the FDR q-value.

Gene Set Name	# Genes	p-value	FDR q-value
GO_MACROMOLECULAR_COMPLEX_BINDING	17	7.49E-17	3.89E-14
GO_TRANSCRIPTION_FACTOR_BINDING	13	8.64E-17	3.89E-14
GO_CHROMATIN_BINDING	11	2.34E-14	7.03E-12
GO_ENZYME_BINDING	16	5.79E-14	1.30E-11
GO_REGULATORY_REGION_NUCLEIC_ACID_BINDING	12	7.90E-13	1.42E-10
GO_RNA_POLYMERASE_II_TRANSCRIPTION_FACTOR_BINDING	7	1.96E-12	2.94E-10
GO_PROTEIN_COMPLEX_BINDING	11	8.81E-11	1.13E-08
GO_DOUBLE_STRANDED_DNA_BINDING	10	2.62E-10	2.95E-08
GO_TRANSCRIPTION_FACTOR_ACTIVITY_PROTEIN_BINDING	9	5.99E-10	5.99E-08
GO_RECEPTOR_BINDING	12	7.06E-10	6.36E-08

Tracking signature activities across cancer timelines

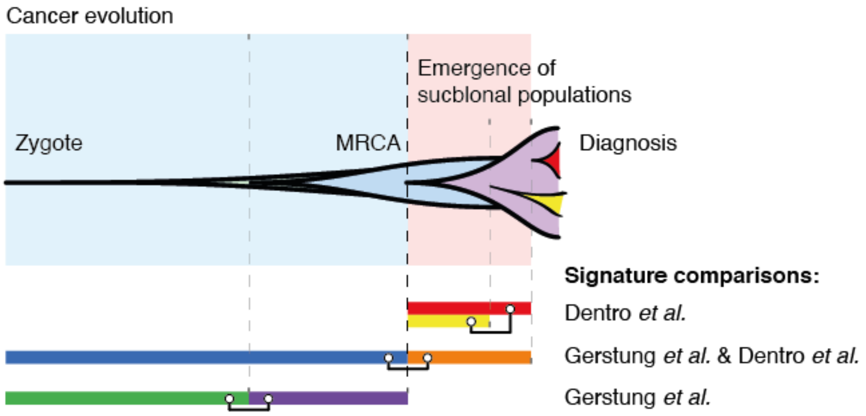

Fig. 30 Timing of mutation signature changes between evolutionary periods. Overview of tumor evolution from zygote to diagnosis, with different measurable "epochs" indicated by colors. Activity changes of mutational processes between these different epochs have been queried both here (Dentro et al.) and by Gerstung et al. (Gerstung et al., 2020) using distinct approaches resulting in differing time resolution.

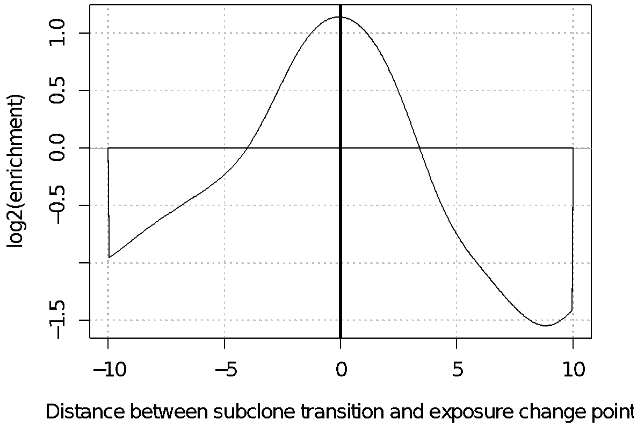

Fig. 31 Changes in signature activities occur near subclone boundaries. Plot displays relative enrichment (y-axis) versus random control of activity change points at given time point offset (x-axis) from subclone boundaries. The smoothing window used when

computing activity trajectories spans three time points, so sub clone boundaries between offsets -3 to 3 are deemed coincident with the activity change points.

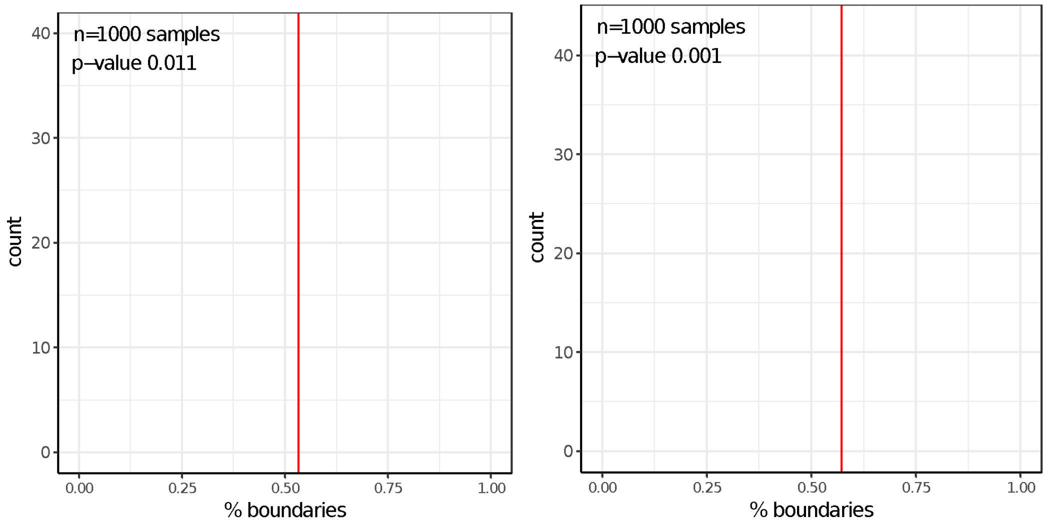

Fig. 32 The distribution of the proportion of boundaries supported by randomly sampled points. The red line shows the proportion of boundaries supported by **real** change-points.

SV analysis and fusion clonality detection

Clonality analysis of recurrent structural variants

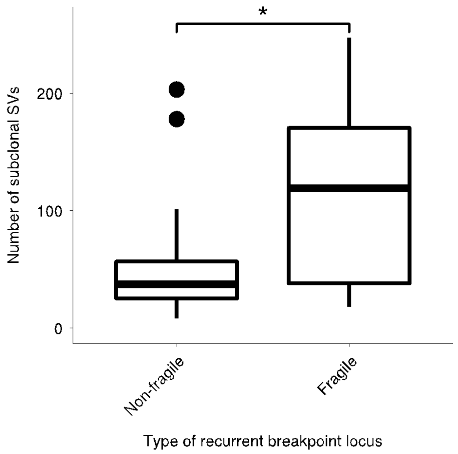

*Fig. 33 Comparison of the number of subclonal SVs observed in SRB loci annotated as fragile or non-fragile. * represents a significant difference (p<0.05) using the Wilcoxon rank sum test.*
